# The multi-omic landscape of transcription factor inactivation in cancer

**DOI:** 10.1186/s13073-016-0342-8

**Published:** 2016-08-25

**Authors:** Andrew E. Teschendorff, Shijie C. Zheng, Andy Feber, Zhen Yang, Stephan Beck, Martin Widschwendter

**Affiliations:** 1 CAS Key Laboratory of Computational Biology, CAS-MPG Partner Institute for Computational Biology, Chinese Academy of Sciences, Shanghai Institute for Biological Sciences, 320 Yue Yang Road, Shanghai, 200031 China; 2 Statistical Cancer Genomics, UCL Cancer Institute, University College London, Paul O’Gorman Building, 72 Huntley Street, London, WC1E 6BT UK; 3 Department of Women’s Cancer, University College London, 74 Huntley Street, London, WC1E 6BT UK; 4 Medical Genomics, UCL Cancer Institute, University College London, Paul O’Gorman Building, 72 Huntley Street, London, WC1E 6BT UK

## Abstract

**Background:**

Hypermethylation of transcription factor promoters bivalently marked in stem cells is a cancer hallmark. However, the biological significance of this observation for carcinogenesis is unclear given that most of these transcription factors are not expressed in any given normal tissue.

**Methods:**

We analysed the dynamics of gene expression between human embryonic stem cells, fetal and adult normal tissue, as well as six different matching cancer types. In addition, we performed an integrative multi-omic analysis of matched DNA methylation, copy number, mutational and transcriptomic data for these six cancer types.

**Results:**

We here demonstrate that bivalently and PRC2 marked transcription factors highly expressed in a normal tissue are more likely to be silenced in the corresponding tumour type compared with non-housekeeping genes that are also highly expressed in the same normal tissue. Integrative multi-omic analysis of matched DNA methylation, copy number, mutational and transcriptomic data for six different matching cancer types reveals that in-*cis* promoter hypermethylation, and not in-*cis* genomic loss or genetic mutation, emerges as the predominant mechanism associated with silencing of these transcription factors in cancer. However, we also observe that some silenced bivalently/PRC2 marked transcription factors are more prone to copy number loss than promoter hypermethylation, pointing towards distinct, mutually exclusive inactivation patterns.

**Conclusions:**

These data provide statistical evidence that inactivation of cell fate-specifying transcription factors in cancer is an important step in carcinogenesis and that it occurs predominantly through a mechanism associated with promoter hypermethylation.

**Electronic supplementary material:**

The online version of this article (doi:10.1186/s13073-016-0342-8) contains supplementary material, which is available to authorized users.

## Background

Transcription factors (TFs) play a central role in development, specifying differentiation and cell fate [1], as well as in reprogramming [2]. Inactivation of TFs that are important for the specification of a tissue type has been proposed as a key mechanism underlying neoplastic transformation of that tissue [3–7]. Biological evidence for this model has recently come from studies showing how genetic mutations in epigenetic regulators such as isocitrate dehydrogenases can result in the inactivation of key transcription factors, promoting cancer [8, 9].

Surprisingly, however, there is a lack of statistical evidence supporting a model in which silencing of transcription factors constitutes a general process underpinning cancer. Arguably, the strongest statistical evidence so far derives from the long-standing observation that bivalently or polycomb repressive complex 2 (PRC2)-marked promoters in human embryonic stem cells (hESCs), which often mark transcription factors that are needed for development and differentiation [10, 11], are significantly more likely to be hypermethylated in cancer [4, 5, 12] and aged normal tissue [13–15] compared with random gene sets. However, even though increased promoter methylation is usually associated with gene silencing, the significance of the observed hypermethylation in cancer is unclear because a large proportion of these bivalently or PRC2-marked TFs are not expressed in the corresponding normal tissue type [16, 17]. Moreover, inactivation of key transcription factors has been associated with other epigenetic alterations such as histone remodelling [8, 9], raising further questions as to the role of the observed DNA hypermethylation in cancer. For instance, epigenetic silencing of *HNF4A* (a key liver-specifying TF) in liver cancer has been linked to loss of promoter H3K4me3 without changes in promoter methylation [8]. Given the large-scale availability of mutational, copy number variation (CNV) and DNA methylation data in primary cancer material, no study has yet systematically explored which mechanism, i.e. mutation, CNV loss, or promoter hypermethylation, is predominantly associated with in-*cis* silencing of transcription factors in cancer.

The purpose of this study, therefore, is to conduct a detailed exploration of the molecular multi-omic landscape of transcription factor inactivation in cancer. We focus our analysis on a subset of bivalently/PRC2-marked transcription factors expressed in a given normal tissue and which are preferentially silenced in the corresponding cancer type. We point out that this is very different from previous studies, which have largely only reported molecular alteration enrichment patterns (mainly DNA methylation) at either the full repertoire of approximately 1500 TFs or the thousands of genes that are bivalently/PRC2-marked in hESCs [4, 5, 12]. The identification of key bivalently/PRC2-marked TFs is achieved by comparing mRNA expression data from hESCs and normal fetal and adult tissues and their corresponding cancer types and studying their patterns of gene expression change across these four phenotypic states. The importance of using normal fetal samples in these types of analyses has recently been highlighted [18], as it allows the confounding effect of age, a major cancer risk factor, to be removed. Having identified the key deregulated TFs in each cancer type, we then perform an integrative multi-omic analysis, encompassing genome-wide mRNA expression, DNA methylation, CNV and somatic mutations for six cancer types, revealing that promoter hypermethylation, and not in-*cis* genomic loss or genetic mutation, is the mechanism that most strongly *associates* with silencing of these transcription factors in cancer.

## Methods

### Definition of initial TF list

We constructed an initial TF gene list as follows. We first used the definition of human TFs, as defined by the Molecular Signatures Database from the Broad Institute (http://software.broadinstitute.org/gsea/msigdb/index.jsp), consisting of a total of 1385 TFs. The most relevant subset of TFs for development and differentiation processes are those which are bivalently or PRC2 marked in hESCs [10, 11]. This resulted in a list of 458 bivalent/PRC2-marked TFs, of which 403 were also present in the Stem Cell Matrix-2 (SCM2) compendium mRNA expression data set.

### The SCM2 compendium data set and identification of TFs expressed in normal tissues

We downloaded the Illumina mRNA expression data of the SCM2 compendium [19, 20]. Expression data were quantile normalized and probes mapping to the same Entrez gene IDs were averaged. This resulted in an expression data set of 17,967 uniquely annotated Entrez gene IDs and 239 samples, including 107 hESC lines, 52 induced pluripotent stem cells and 32 somatic differentiated tissue samples, with the rest of the samples representing human cell lines. Among the 32 somatic differentiated tissue samples, we selected those epithelial tissues for which there were at least two samples and for which we could identify corresponding cancer data sets from The Cancer Genome Atlas (TCGA). In cases where fetal and adult samples were available, we used fetal samples since these are of age zero, thus eliminating age as a potential confounder [18]. These epithelial tissues included bladder (two adult samples), lung (two fetal samples), kidney (two fetal samples), colon (one fetal and one adult sample) and stomach (three fetal samples). However, the stomach samples were not considered further because the top principal component of variation in the corresponding stomach adenocarcinoma (STAD) TCGA data set correlated with an unknown confounding factor, most likely representing cellular heterogeneity. Thus, for each of the four cell types (lung, kidney, colon and bladder), we derived statistics of differential expression for all 17,967 genes compared with the 107 hESC lines using an Bayes model [21] as implemented in the *limma* Bioconductor package [22].

### TCGA data

We downloaded TCGA data (as provided by TCGA website), including all level 3 CNV, RNA-Seq (V2) and Illumina 450k DNA methylation data, in addition to somatic mutational information, for a total of six cancer types, including lung adenoma carcinoma (LUAD) [23], lung squamous cell carcinoma (LSCC) [24], kidney renal cell carcinoma (KIRC) [25], kidney renal papillary carcinoma (KIRP) [26], bladder carcinoma (BLCA) [27], colon adenoma carcinoma (COAD) [28] and stomach adenomacarcinoma (STAD) [29]. Illumina 450k DNA methylation data were further processed using BMIQ to adjust for the type 2 bias [30]. In the case of RNA-Seq level 3 data, genes with zero read counts in all samples or exhibiting no variation across samples were removed. RNA-Seq level 3 data were subsequently regularised using a log2 transformation. Normalized RNA-Seq and DNA methylation data sets were subjected to an additional quality control procedure which used a singular value decomposition to assess the nature of the top components of variation [31]. According to this analysis, the STAD TCGA dataset was not considered further due to the top component of variation not correlating with normal/cancer status, an indicator of substantial confounding variation [31].

In the case of mutational data, somatic mutations were classed as inactivating mutations if they were nonsense, missense or deletions. For a given tumour sample and gene, multiple inactivating mutations in the same gene were treated as one. In the case of CNV data, we used the normalised segment values as provided by the level 3 standard.

### Differential expression and differential DNA methylation analyses

Differential gene expression analysis for the normalized RNA-Seq data between normal and cancer tissue was performed using an empirical Bayes model [21] as implemented in the *limma* Bioconductor package [22]. The numbers of normal and cancer samples were 58 and 471 for LUAD, 45 and 473 for LSCC, 72 and 515 for KIRC, 32 and 289 for KIRP, 17 and 323 for BLCA and 41 and 270 for COAD.

In the case of Illumina 450k DNA methylation data we used a recursive model, validated by us previously [32], to assign a DNA methylation (DNAm) level to each gene. Specifically, this model first assigns the average DNAm value of probes mapping to within 200 bp upstream of the transcription start site. If no 450k probes map to this region, first exon probes are used instead. If there are no first exon 450k probes for a given gene, we use the average over 450k probes mapping to within 1500 bp upstream of the transcription start site . As shown by us previously, the average DNAm of 450k probes in these regions provides the best predictive model of a sample’s gene expression value [32]. The same empirical Bayes model was then used to derive statistics of differential DNA methylation between normal and cancer tissue. The numbers of normal and cancer samples for the differential DNAm analysis were 41 and 275 for LSCC, 32 and 399 for LUAD, 160 and 299 for KIRC, 45 and 196 for KIRP, 19 and 204 for BLCA and 38 and 272 for COAD.

### Definition of control non-housekeeping gene sets

In order to objectively assess whether TFs overexpressed in a normal tissue type relative to hESCs exhibit preferential downregulation in the corresponding cancer type, a comparison with a control set of non-housekeeping genes is needed. This control set of genes was constructed for each TCGA cancer set separately as we needed to select genes with similar expression levels to the TFs in the normal-adjacent samples of TCGA set. Having identified a matching set, we then removed all housekeeping genes using the comprehensive list of 3804 housekeeping genes from Eisenberg and Levanon [33]. Thus, the control set of genes consists of non-housekeeping genes expressed at the same level in normal-adjacent tissue as the given TFs.

### Integrative matched tumour analyses

In order to identify the tumours where a given tissue-specific TF is underexpressed, we derived a *Z*-score for each tumour and TF by comparing its TF expression level with the mean and standard deviation of expression as evaluated over all corresponding normal tissue samples. Specifically, if *t* labels the TF and *μ*_*t*_ and *σ*_*t*_ label the mean and standard deviation in expression of this TF over the normal tissue samples, then the *Z*-score of TF *t* in sample *s* is defined by *Z*_*ts*_ = (*X*_*ts*_ − *μ*_*t*_)/*σ*_*t*_. We deemed a TF to be underexpressed in sample *s* if the corresponding *Z*-score was less than −2, corresponding to a *P* value of ~0.05. For the tumours exhibiting underexpression of the TF, we then defined a genomic loss if the segment value corresponding to the TF locus had a value less than −0.35 (we estimated a conservative threshold of one-copy gain/loss to be at around ±0.35). For tumours exhibiting underexpression of the TF, we also considered the promoter of the TF to be significantly hypermethylated if the difference in DNA methylation between the tumour and the average of the normal samples was larger than 0.3. This estimate is justified from scatterplots of promoter DNAm versus log2[RNA-Seq counts] for all genes in normal samples, which shows that promoter DNAm increases of 0.3 or higher are much more likely to be associated with gene silencing. In the case of DNAm, an alternative approach could have been to define an analogous *Z*-score of DNAm change in relation to the normal tissue. However, this could generate large statistics without necessarily a big change in absolute DNAm levels; given that the purpose was to see if the DNAm change could account for the change in gene expression, we focused on using absolute differences in DNAm levels.

For the integrative analyses where the matched nature of the samples was used, analysis was restricted to normal and cancer samples with matched DNAm, CNV and mRNA expression data. The numbers of normal and cancer samples for these matched analyses were 8 and 273 for LSCC, 20 and 390 for LUAD, 24 and 292 for KIRC, 21 and 195 for KIRP, 13 and 194 for BLCA and 19 and 253 for COAD.

## Results

### Identification of transcription factors important for tissue differentiation

**Fig. 1:**
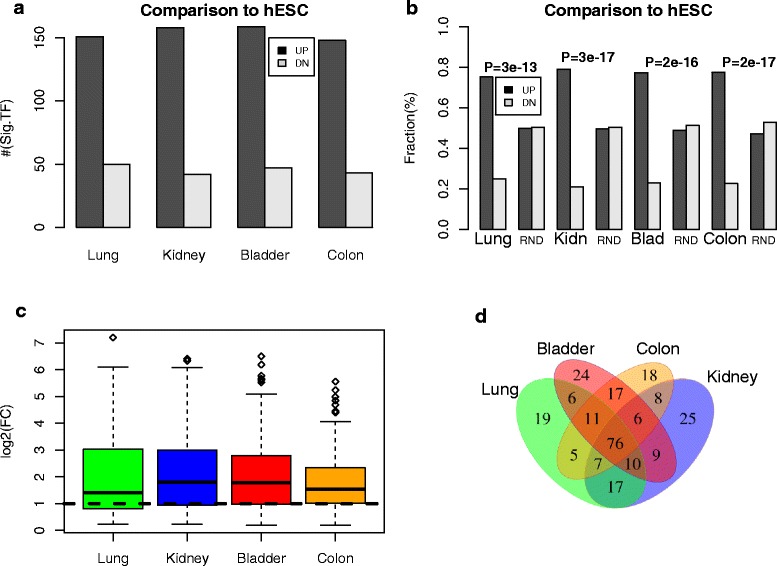
Identification of transcription factors that are important for differentiation. **a** Relative numbers of significantly upregulated (*UP*) and downregulated (*DN*) transcription factors (*TF*) in specific normal tissues relative to human embryonic stem cells (*hESC*). In the case of lung, kidney and colon, fetal tissue was used to ensure that the comparison is not confounded by age effects. **b** As **a** but now expressing the relative numbers of differentially expressed TFs as fractions and comparing these fractions with those of 1000 randomly selected genes (*RND*). *P* values are from a one-tailed Fisher’s exact test, demonstrating that most differentially expressed bivalently marked transcription factors in hESCs become upregulated upon differentiation. **c** Distribution of log2 fold changes (*log2(FC)*) for the significantly upregulated TFs in each tissue type, demonstrating that most upregulated TFs exhibit at least twofold changes in expression. **d** Upregulated TFs, identifying common and “tissue-specific” TFs

We posited that TFs with important roles in differentiation and cancer could be identified by analyzing their dynamic expression changes between four main cellular states: the hESC state, a partially differentiated normal fetal state, an adult normal differentiated state and the undifferentiated cancer state. Indeed, as already shown by others in the context of development [1], focusing on dynamic changes in gene expression can successfully identify key TFs. Thus, we initially aimed to identify TFs which become overexpressed in a number of normal tissue types, relative to the hESC ground state, using data from the Stem Cell Matrix-2 (SCM2) compendium [19, 20] (“Methods”). An advantage of using the SCM2 data is the availability of mRNA expression data generated with the same array platform for both hESCs and somatic primary cells for a number of different tissue types, including both fetal and adult states to avoid confounding by age (“Methods”). We restricted the analysis to somatic tissue types for which there were at least two independent samples in the SCM2 compendium and for which there was corresponding high-quality tissue data from TCGA. In total, we identified four tissue types for which matching data in SCM2 and TCGA were available: this included lung, kidney, bladder and colon. Comparison of mRNA expression levels between hESCs (a total of 107 hESC samples derived from both male and females and from a wide range of different passages) and the foetal/adult normal samples from lung, kidney, bladder and colon were performed, focusing on a set of 403 bivalently [10] or H3K27me3 (PRC2) [11] marked TFs in hESCs (“Methods”; Additional file 1: Table S1), since it is well known that their poised promoters in the hESC state mark TFs which are needed for differentiation [10, 11]. We observed that approximately 200 (i.e. 50 %) of these 403 TFs exhibited significant differential expression relative to the hESC state, a result which was largely independent of tissue type (Fig. 1a). Among the significantly differentially expressed TFs, around 150 (i.e over 70 %) were overexpressed in the differentiated tissue, supporting their role in differentiation (Fig. 1a, b; Additional file 1: Tables S2–S5). We verified that the overwhelming majority of these significantly overexpressed TFs exhibited fold changes larger than two (Fig. 1c), further supporting their significance. In total, 76 overexpressed TFs were common to all four tissue types, with 19, 25, 24 and 18 being overexpressed in only lung, kidney, bladder and colon, respectively (Fig. 1d).

### Bivalent/PRC2-marked TFs expressed in a tissue type are preferentially silenced in the corresponding cancer type

**Fig. 2:**
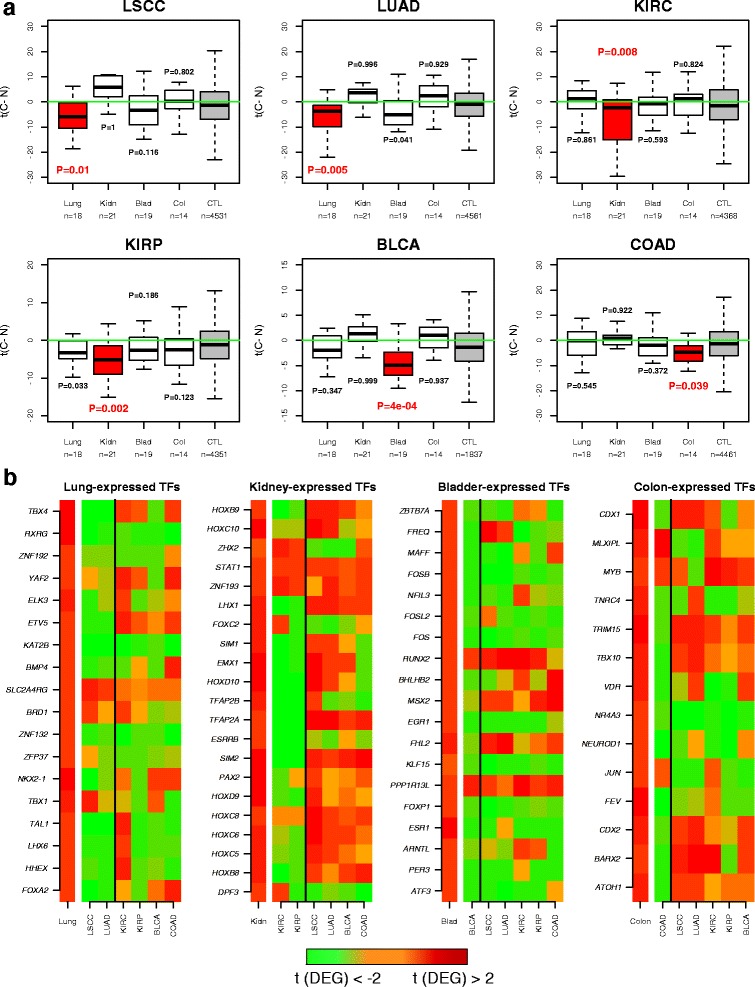
Transcription factors expressed in normal tissue are preferentially silenced in the corresponding cancer type. **a** Boxplots of t-statistics of differential mRNA expression between cancer and normal tissue (*y-axis*, *t(C − N)*) for four sets of “tissue-specific” TFs and a control set of genes (*CTL*) across six different cancer types, as indicated. *LSCC* lung squamous cell carcinoma, *LUAD* lung adenoma carcinoma, *KIRC* kidney renal clear cell carcinoma, *KIRP* kidney renal papillary carcinoma, *BLCA* bladder carcinoma, *COAD* colon adenoma carcinoma. The five sets of genes being compared are the TFs expressed in the relevant normal tissue (*red box*), the TFs expressed in other normal tissue types (*white boxes*) and a set of control (*CTL*, *grey box*) non-housekeeping genes which are expressed at a similar level to the TFs expressed in that same normal tissue. *P* values are from a one-tailed Wilcoxon-rank sum test comparing the t-statistics of each group of TFs with the control (*CTL*) gene set. We note that negative t-statistics means lower expression in cancer compared with normal. **b** Heatmaps depicting the dynamics of gene expression changes of the tissue-specific TFs expressed in the normal tissue. t-statistics of differential expression (*t(DEG)*), are shown between hESCs and normal tissue (the *left-most colour heatmap* in each panel) and between normal tissue and various cancer types (the *right heatmap* in each panel), as indicated. We note that the heatmap to the very left in each panel is always *red*, indicating the overexpression of these TFs in foetal/adult normal tissue compared with hESCs. The heatmap representing the t-statistics of differential expression between normal tissue and the corresponding cancer types are shown to the *left* of the *vertical black line*, whereas those for the other unrelated cancer types are shown to the *right*. There is generally more green (i.e. underexpression) in the cancer types matching the tissue types compared with the other cancer types, in agreement with the data shown in **a**

We hypothesized that TFs which are important for differentiation of a tissue type, and which are, therefore, expressed in that tissue type, may be under selection pressure to undergo silencing in the corresponding cancer type. To formally test this, we collected RNA-Seq data from TCGA for two types of lung cancer (LSCC and LUAD), two types of kidney cancer (KIRC and KIRP), BLCA and COAD. In order to draw a statistically valid conclusion in each normal–cancer TCGA dataset, we need to compare the statistics of differential expression of *mutually exclusive* sets of TFs. Hence, we first focused on the previously identified 19 lung-, 25 kidney-, 24 bladder- and 18 colon-specific TFs, most of which (18, 21, 19 and 14, respectively) were also highly expressed in the respective normal tissue samples from TCGA. In order to assess the biological and statistical significance, the comparison of these sets of TFs was made to a common control set of genes (CTL) expressed at the same level in the normal tissue as the given TFs and which excluded any of 3804 well-established housekeeping genes [33] (Additional file 1: Figure S1). We observed that the great majority of the identified TFs were significantly downregulated in the corresponding cancer type, with the identified TFs more likely to be downregulated in the corresponding cancer type compared with the control set of genes (Fig. 2a; Additional file 1: Tables S6–S9). Thus, the silencing of these TFs in cancer is not merely determined by their relatively high expression levels in the normal tissue since a control set of non-housekeeping genes expressed at the same level in normal tissue (Additional file 1: Figure S1) did not show the same level of downregulation in cancer (Fig. 2a). As expected, the promoters of the silenced TFs were significantly more likely to map to a CpG island owing to the fact that we initially restricted the analysis to bivalently and PRC2-marked TFs (Additional file 1: Table S10).

Next, we decided to relax the definition of tissue-specific TFs to allow any TF expressed in a given normal tissue regardless of its expression level in other normal tissue types. This more inclusive definition recognizes that cell and tissue types are arranged in a hierarchical developmental tree, as it is well known that TFs important for specification of one tissue type are also important for specification of other tissues. As a concrete example, *FOXA1* (*HNF4A*) is a transcription factor important for the specification of the intestine and stomach [34, 35] as well as liver [36] and silencing of *HNF4A* leads to liver cancer [8]. Similarly, GATA factors such as *GATA4* play key roles in the development of the gastrointestinal tract [37–39] as well as in the development of the heart [40], pancreas [41] and liver [42], and so these factors could play tumour-suppressor roles in many different cancer types [39, 43]. Hence, TFs expressed in multiple normal tissue types can be as important to the development of a specific cancer type than TFs which are expressed only in the corresponding normal tissue type. Thus, on biological grounds, we re-assessed the previous result, now considering all TFs expressed in a normal tissue regardless of their expression levels in the other normal tissues types. In spite of the fact that these TF sets are largely overlapping, we still observed that the strongest underexpression was in the corresponding cancer type and that it was highly significant when compared with a control set of non-housekeeping genes expressed at a similar level in the same normal tissue (Additional file 1: Figures S3 and S4).

Among the silenced TFs were many well known differentiation factors (Fig. 2b). For instance, in lung we found *FOXA2* [44], *TBX4* [45] and *BMP4* [46], and although the role of *LHX6* in lung development is less well defined, it has previously been implicated as a tumour suppressor in lung cancer [47]. Similarly, in kidney we observed many TFs implicated in kidney development, including HOX family genes [48], *ESRRB*/*ESRRG* [49], *PAX2* and *LHX1* [50, 51]. In the case of bladder cancer, TFs which have been previously implicated in urothelial cell differentiation, such as *RARA* and *KLF4* [52], were observed to be upregulated in bladder tissue compared with hESCs (Additional file 1: Table S4) and also subsequently silenced in bladder cancer (Additional file 1: Figure S2), although they were also observed to be upregulated in kidney or lung tissue (Additional file 1: Tables S2 and S3). In the case of colon cancer, silenced TFs included well known intestinal differentiation factors such as *CDX1* [53, 54], *CDX2* [55, 56] and *NEUROD1* [57, 58]. Thus, our approach successfully identifies TFs silenced in cancer and which have been previously implicated in the differentiation of the corresponding tissue types.

### Promoter hypermethylation, and not CNV loss or mutation, associates most strongly with silencing of bivalent/PRC2-marked TFs in cancer

**Fig. 3:**
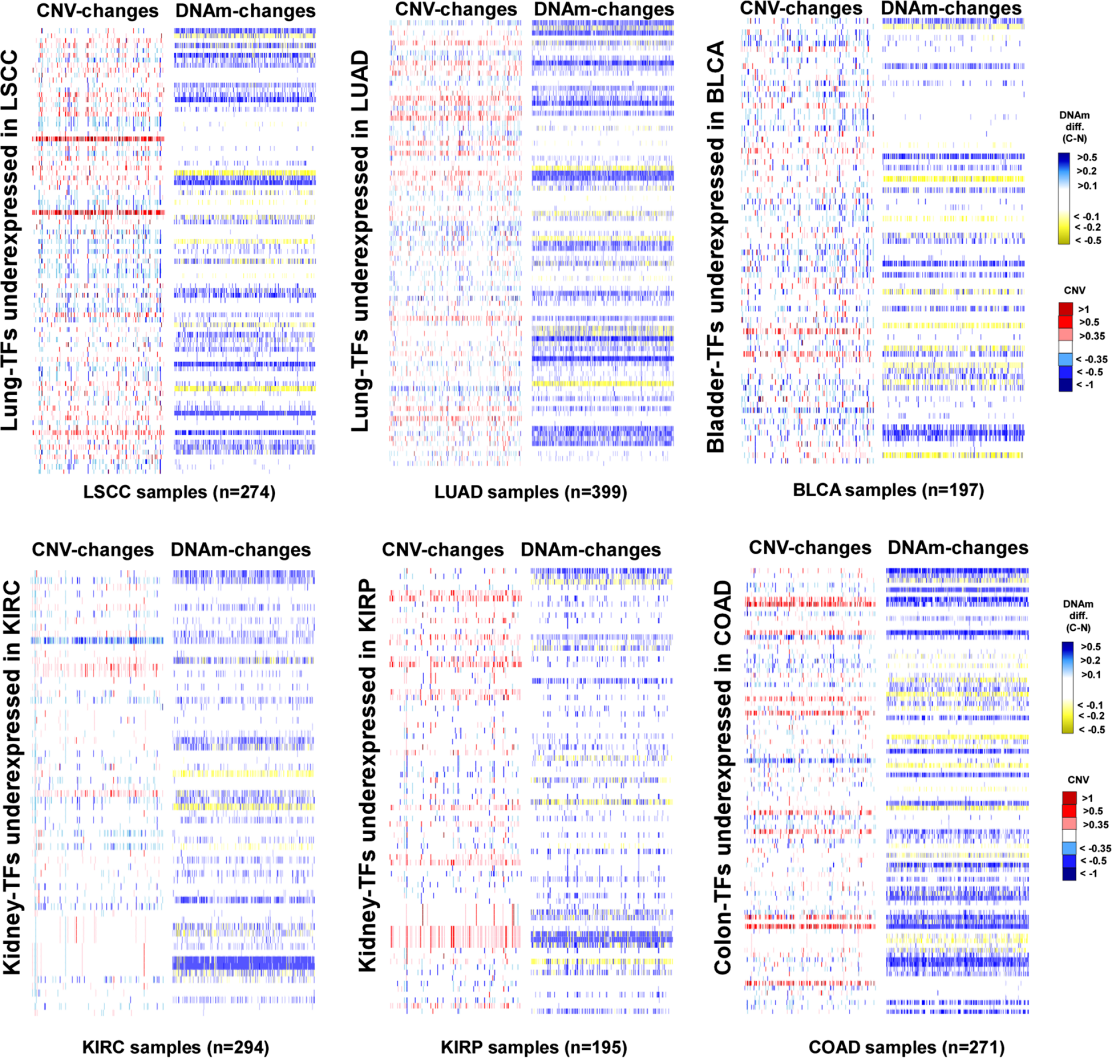
Landscape of CNV and promoter methylation of TFs that are silenced in cancer. Heatmaps of copy number and promoter methylation changes in six different cancer types: LSCC, LUAD, KIRC, KIRP, BLCA and COAD. In each case TFs highly expressed in the corresponding normal tissue type and which are underexpressed in cancer have been arranged along rows, using the same order for DNAm and CNV. Each column in the heatmap labels a tumour sample. For all CNV heatmaps, the colours represent the segment values assigned to the corresponding genes, as indicated. In the case of DNAm, the colours indicate the difference in beta (DNAm) value at the promoter between the cancer sample and the average of all normal tissue samples

We next asked which type of molecular alteration associates most strongly with silencing of bivalently/PRC2-marked TFs in cancer. For this analysis, we considered all TFs overexpressed in a given normal tissue type (compared with hESCs) and underexpressed in cancer (compared with its respective normal tissue), without the requirement that they be overexpressed in only one normal tissue type. We obtained CNV, somatic mutation as well as DNAm data for all genes and for all cancer types considered previously (“Methods”). Depicting the copy number and DNAm changes of these silenced TFs between cancers and their corresponding normal samples revealed a striking difference between DNAm and CNV (Fig. 3; Additional file 1: Figures S5–S10). Whereas at the genomic copy number level we did not observe a preference for these TFs to undergo copy number loss, at the level of DNA methylation there was a clear skew towards increased promoter DNAm (Fig. 3; Additional file 1: Figures S5–S10).

**Fig. 4:**
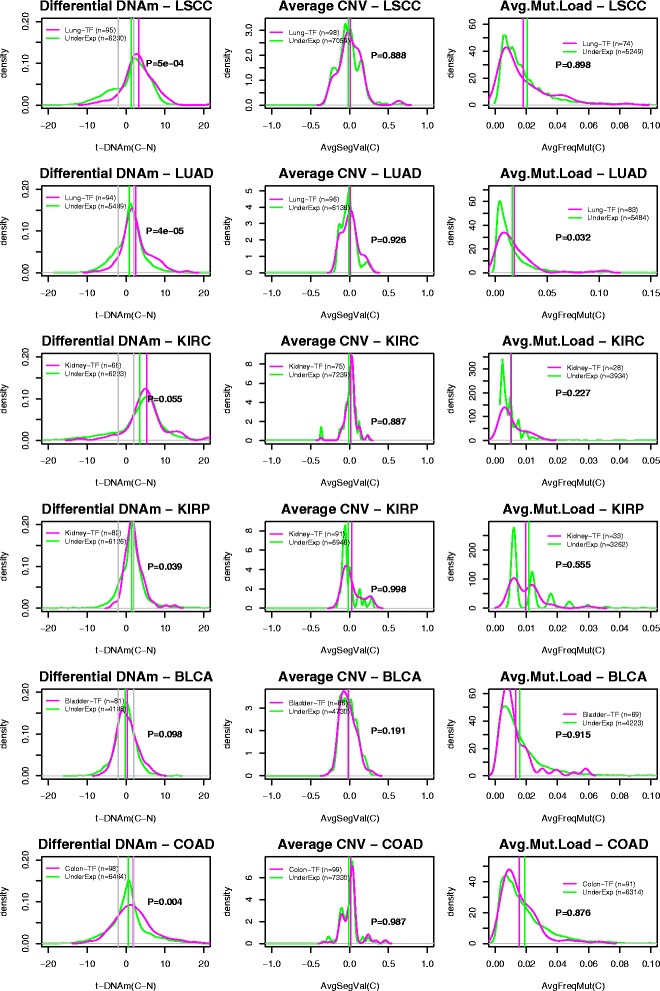
Transcription factors expressed in normal tissue and silenced in cancer predominantly exhibit promoter hypermethylation and not genomic loss or inactivating mutation. *Left panels*: density plots of t-statistics of differential DNAm between cancer and normal tissue (*x-axis*, *t(C − N)*) of the tissue-specific cancer-silenced TFs (*magenta lines*) compared with the corresponding density distribution of all genes underexpressed in cancer (*green lines*). Density plots are shown for six cancer types: LSCC, LUAD, KIRC, KIRP, BLCA and COAD. *P* values are from a Wilcoxon rank sum test. The *vertical magenta* and *green lines* denote the average levels. The *grey vertical lines* in the DNAm plot indicate *P* = 0.05. *Middle panels*: as above but for the average CNV segment values of the TFs (*magenta lines*) and all underexpressed genes (*green lines*). *Right panels*: as above but for the frequency of inactivating mutation of the TFs (*magenta lines*) and all underexpressed genes (*green lines*)

In order to assess the statistical and biological significance of these observations, we next compared the degree of molecular alteration of the silenced TFs with that of all genes underexpressed in the given cancer type, as well as to a randomly chosen set of genes, a procedure which adjusts for the differential sensitivity of the different molecular assays. We observed that average genomic loss levels of the silenced TFs were generally not significantly higher than that of underexpressed genes or that of a randomly chosen set of genes (Fig. 4; Additional file 1: Figure S11). Likewise, the average frequency of inactivating mutations of these TFs across cancers was generally not higher compared with underexpressed genes or randomly selected genes (Fig. 4; Additional file 1: Figure S11). In contrast, differential promoter methylation statistics of the silenced TFs were generally significantly higher compared with those of underexpressed or randomly chosen genes (Fig. 4; Additional file 1: Figure S11). In general, for each cancer type there were more TFs and tumours with significant positive differential methylation statistics than the corresponding expected number had the genes been drawn from the set of all cancer underexpressed genes (Additional file 1: Figure S12). This result was also evident if significance in a tumour is defined by a TF exhibiting a promoter DNAm increase of at least 30 % compared with the average over normal samples (Additional file 1: Figure S13). Using a meta-analysis over all cancer types, it was only for the case of promoter hypermethylation that we observed a significantly higher level of alteration at the silenced TFs compared with all underexpressed genes (Table 1; *P* < 10^−8^ for promoter hypermethylation, *P* = 0.98 for CNV loss and *P* = 0.47 for mutation, combined Fisher test). We note that if we compared *all* underexpressed genes in a given cancer type to a randomly selected set of genes, then all molecular categories were significant, consistent with the view that all molecular events, be it promoter hypermethylation, CNV loss or inactivating mutation, are associated with underexpression in cancer (Additional file 1: Figure S14). In summary, the data shown in Fig. 4 and Table 1 suggest that promoter hypermethylation is the more likely mechanism associated with in-*cis* TF silencing in cancer.

**Table 1:** Silenced TFs in cancer undergo preferential promoter hypermethylation in comparison with all cancer underexpressed genes

	nTF	nU	*P*(DNAm)	*P*(CNV)	*P*(Mutation)
LSCC	95	7697	0.0005	0.888	0.898
LUAD	94	6673	0.00004	0.926	0.032
KIRC	68	7814	0.055	0.887	0.227
KIRP	82	7465	0.039	0.998	0.555
BLCA	81	5097	0.098	0.191	0.915
COAD	98	7934	0.004	0.987	0.876
Combined Fisher test			<1e-8	0.984	0.471

The first six rows label the TCGA cancer type. Columns label the number of TFs expressed in the normal tissue and underexpressed in cancer (*nTF*), the number of all genes underexpressed in cancer (*nU*), the *P* value from a Wilcoxon rank sum test assessing whether promoter hypermethylation at the TFs is more frequent than for all underexpressed genes with DNAm information (*DNAm*), the *P* value from a Wilcoxon rank sum test assessing whether CNV loss of the TFs is more frequent than for all underexpressed genes with CNV information (*CNV*) and the *P* value from a Wilcoxon rank sum test assessing whether inactivating mutation of the TFs is more frequent than for all underexpressed genes with mutational information (*Mutation*). The last row lists *P* values of a meta-analysis over all cancer types using a combined Fisher test

**Fig. 5:**
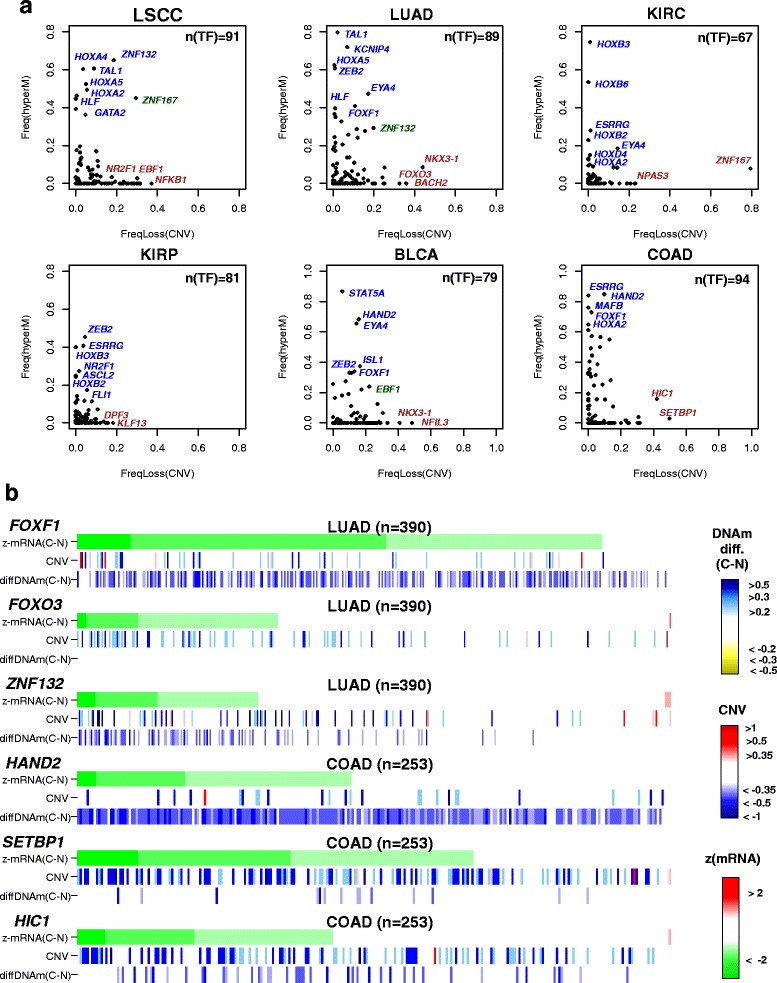
Cancer-silenced TFs exhibiting different propensities to undergo promoter DNA methylation or genomic loss in cancer. **a** Scatterplots of the frequency of genomic loss (*x-axis*) against promoter hypermethylation (*y-axis*) in cancer, as estimated over tumours exhibiting underexpression of the given TF. Each *data point* in the scatterplots represents one silenced TF. Some of the TFs exhibiting more propensity to undergo promoter DNAm than CNV loss are shown in *blue*, some TFs exhibiting less propensity to undergo promoter DNAm than CNV loss are shown in *brown*, and in *green* we highlight some TFs exhibiting both frequent CNV loss and promoter hypermethylation. **b** Heatmap representations of mRNA expression change (z-statistics of mRNA expression change), CNV and DNAm change (difference in beta-value between cancer and all normals) for a number of silenced TFs exhibiting different propensities for promoter hypermethylation and CNV loss in two different cancer types (LUAD and COAD), as indicated. Tumour samples are sorted in decreasing order of underexpression in cancer

Next, we decided to extend the previous analysis to the single-sample level in order to investigate the detailed pattern of promoter methylation and CNV within individual tumours. We first considered for each TF in each cancer type those tumours which exhibited significant underexpression relative to the respective normal tissue (“Methods”). For each TF and across all tumours exhibiting underexpression of this TF, we then counted the fraction of tumours exhibiting genomic loss of the TF, as well as the fraction of tumours exhibiting hypermethylation of the TF’s promoter (“Methods”). In general, this revealed that promoter hypermethylation events could account for a higher fraction of cancers exhibiting underexpression of the corresponding TF compared with genomic loss (Fig. 5a). For instance, in LSCC we observed four TFs (*HOXA4*, *HOXA5*, *TAL1*, *ZNF132*) undergoing promoter hypermethylation in at least 50 % of the LSCC tumour samples where these TFs were underexpressed. In contrast, no TF was observed to undergo CNV loss at a frequency of over 50 % in the same cancers (Fig. 5a). A similar observation was evident for LUAD (Fig. 5a). In the case of KIRP we observed six TFs exhibiting promoter hypermethylation at over 20 % of the tumours with underexpression of the TF, in contrast to no TF exhibiting CNV loss at that frequency or higher (Fig. 5a). This pattern of more frequent promoter hypermethylation than CNV loss was also evident for BLCA and COAD (Fig. 5a).

### Some silenced bivalent/PRC2-marked TFs exhibit patterns of mutual exclusivity between promoter hypermethylation and CNV loss

Interestingly, we observed that many TFs exhibiting a higher frequency of CNV loss in cancer did not show appreciable promoter DNAm increases in any of the tumour samples, suggesting that some TFs are more intrinsically prone to genomic loss (Fig. 5a). Indeed, broadly speaking, there were three types of silenced TFs in each cancer type (Fig. 5b): those predominantly exhibiting promoter hypermethylation but with relatively few CNV losses (e.g. *FOXF1* in LUAD, *HAND2* in COAD), those exhibiting frequent CNV loss but not many DNAm changes (e.g. *NR2F1* in LSCC, *FOXO3* in LUAD, *SETBP1* in COAD) and a third class of TFs which exhibited both CNV loss and promoter hypermethylation (e.g. *ZNF132* in LUAD, *HIC1* in COAD).

To investigate if there is any evidence for mutual exclusivity between promoter hypermethylation and CNV loss, we next compared the frequency of TF promoter hypermethylation between the top and lowest tertiles of TFs ranked by CNV loss frequency. This revealed a higher frequency of hypermethylation for those TFs undergoing the least CNV losses (Additional file 1: Figure S15a; combined Fisher test *P* = 0.002), consistent with the observed “L” type shapes of the scatterplots (Fig. 5a). The reverse analysis, comparing the frequency of CNV loss between the top and lowest tertiles defined according to the frequency of hypermethylation, also revealed a consistent pattern of mutual exclusivity (Additional file 1: Figure S15b; combined Fisher test *P* = 0.004).

Focusing on TFs undergoing both CNV loss and promoter hypermethylation (at least 1 % frequency for both types of alteration) revealed only a few (*EBF1* in LSCC, *LYL1* in LUAD, *ZNF287* in BLCA and *HIC1* in COAD) which did so in a mutually exclusive fashion, in the sense of exhibiting higher levels of hypermethylation in tumours with no CNV loss of the given TF, compared with tumours with CNV loss, although this was only evident if the previous threshold for calling significant promoter hypermethylation (i.e. 0.3) was relaxed to a value of 0.1 (Additional file 1: Figure S16).

### Bivalent/PRC2-marked TFs silenced in multiple cancer types are more likely to share aberrant promoter hypermethylation

Next, we asked if the mechanism associated with silenced TFs is similar between cancer types. For this analysis, we focused on TFs that were commonly silenced across cancer types. As expected, LSCC and LUAD shared a strong overlap of 80 TFs (~88 %) silenced in both cancer types, whilst the smallest overlap was between BLCA and KIRC (18 TFs). Frequencies of promoter hypermethylation of commonly silenced TFs were highly correlated between every pair of cancer types (average R^2^ value was 0.39; Additional file 1: Figure S17). In contrast, correlations were significantly lower in the case of CNV loss (average R^2^ value was 0.23, Wilcoxon rank sum paired test *P* = 0.005; Additional file 1: Figure S18). This suggests that TFs silenced in multiple cancer types are more likely to be associated with promoter DNA hypermethylation than with in-*cis* CNV loss.

## Discussion

Although impairment of differentiation is a well known cancer hallmark, only a few concrete examples of TF inactivation have been shown to block differentiation and predispose to epithelial cancer [8, 9]. Since the experimental identification of TFs necessary for tissue specification is cumbersome, we here took an in silico approach, comparing mRNA expression levels of a relevant subset of TFs (bivalently and PRC2-marked) between hESCs and normal foetal/adult tissue in order to identify TFs which become strongly overexpressed upon differentiation. We hypothesized that if blocks in differentiation constitute a key process contributing to carcinogenesis, these highly expressed TFs would be frequently silenced in cancer and they would do so preferentially in comparison with other non-housekeeping genes which are highly expressed in the same tissue. Using six different cancer types, we were able to confirm that TFs overexpressed in a normal tissue type relative to a hESC ground state are preferentially silenced in the corresponding tumour type. These TFs likely represent tumour suppressors. Our second main contribution is the demonstration that silencing of these TFs is associated mainly with promoter hypermethylation and not with in-*cis* genomic loss or mutation. Importantly, for many TFs, promoter hypermethylation could account for the largest fractions of tumours exhibiting underexpression of that TF. Indeed, whereas CNV loss and inactivation mutations are known to affect tumour suppressors, the frequencies of these events across tumours of a given cancer type are generally quite low, making it difficult to identify novel cancer driver genes [59]. In contrast, promoter hypermethylation at specific TFs is a much more frequent event, supporting a role for epigenetic-mediated silencing in the suppression of key tumour suppressors [60]. However, we also observed silenced TFs which were only prone to CNV loss with no observed promoter hypermethylation across tumours. In addition, we also identified a few examples of silenced TFs exhibiting both CNV loss and promoter hypermethylation in a mutually exclusive fashion.

While these novel insights support the view that promoter hypermethylation of lineage-specifying TFs could be a key step in carcinogenesis, it is equally important to point out limitations in our analysis. First of all, it is important to stress that the observed correlations between promoter DNAm and underexpression are only associative. Demonstrating that the observed promoter hypermethylation causes TF underexpression is beyond the scope of this study. Moreover, we can’t exclude the possibility that inactivation of an upstream TF, through genomic loss or mutation, underlies the loss of binding and hence increased DNAm at the promoters of the observed TFs. Indeed, several studies have shown how hypermethylation at both promoters and distal regulatory elements such as enhancers can result from deletion of specific TFs [61]. Also, the important role of DNAm alterations at super-enhancers and associated DNAm and mRNA expression changes at linked gene promoters in cancer has recently been noted [62]. Thus, our data can’t distinguish between a causative model, in which promoter hypermethylation causes the observed underexpression of the TFs, from an effects model, in which the observed hypermethylation and silencing is the consequence of an upstream TF inactivation event, be this a CNV loss, inactivating mutation, promoter methylation or increased methylation at an enhancer. The associative statistical analysis presented here suggests, however, that, *probabilistically*, promoter hypermethylation of a TF is a more likely mechanism than CNV loss or an inactivating mutation.

A second limitation of our analysis is that we did not consider the role of non-coding RNAs, in particular that of microRNAs (miRNAs). In common with TFs, miRNAs play an important role in development and cellular differentiation, with many playing a tumour-suppressive role in cancer [63, 64]. Moreover, it has recently been noted that bivalently marked miRNA promoters are also frequently hypermethylated in cancer, with many of these also exhibiting underexpression [65]. It will be interesting, therefore, to explore if miRNAs highly expressed in a given tissue type also exhibit preferential downregulation in the corresponding cancer type and whether, for this particular subset of downregulated miRNAs, promoter hypermethylation is also the main associative mechanism. Likewise, in this study we did not consider the important role of histone modifications, which we know are altered in cancer and which could also result in epigenetic silencing of key TFs, as observed, for instance, in the case of *HNF4A* in liver cancer, where the reduced expression has been attributed to a loss of H3K4me3 [8, 66]. Unfortunately, histone modification data for the matched TCGA samples considered here are not available. In future, however, it will be important to include ChIP-Seq profiles for all major regulatory histone marks in these comparative analyses.

A third caveat in our analysis is that the inferred underexpression of TFs in cancer was done by comparison with a normal reference defined by normal tissue that is found adjacent to the tumour specimen. This normal-adjacent tissue may already contain age-associated epigenetic field defects [67], which may reduce the sensitivity to detect silencing events in cancer. For instance, GATA4 is a well known differentiation factor for a number of different tissue types, including colon tissue [39]. Although we did observe GATA4 to be overexpressed in foetal colon tissue compared with hESCs, its level of mRNA expression in the normal colon tissue adjacent to colorectal cancer samples was surprisingly low, which is why we did not see further underexpression of this TF in colon cancer. A potential explanation for this is that GATA4 is already gradually silenced in aged colon tissue as a result of age-associated promoter hypermethylation [13], with the aggravated hypermethylation in cancer not causing any further change in gene expression. Direct comparison with a purified age-matched sample representing the cell of origin could overcome some of these limitations. A related caveat in our analysis is cellular heterogeneity, as it is possible that the cell of origin of the cancer is underrepresented in the normal tissue, confounding the differential expression analysis, although this is less likely to be the case for normal tissue found adjacent to the cancer.

Another limitation is the restriction to four tissue types (lung, kidney, bladder and colon). This restriction merely reflects the availability of mRNA expression data in the original SCM2 compendium which simultaneously profiled hESCs and primary differentiated cells for a number of different tissue types. Given that study-specific batch effects are notorious in gene expression data [68], the requirement that expression profiles from hESCs and differentiated tissue come from the same study is critical. Analysis of a more comprehensive compendium of hESC and differentiated primary samples using RNA-Seq data will be needed to assess whether the findings reported here generalize to other tissue types. However, in spite of only analyzing four normal tissues and six cancer types, our results are highly statistically significant when interpreted in the context of a meta-analysis (see e.g. Table 1).

Finally, we stress that most of the analyses presented here were performed on TFs expressed in a normal tissue type, regardless of their expression levels in other normal tissues. Although this entails a much more liberal definition of “tissue specificity”, it is also the most biologically meaningful one to consider. For instance, as remarked earlier, *HNF4A* is a TF which is needed for liver specification, silencing of it leading to liver cancer [8], yet it is also expressed in other tissue types such as kidney and stomach [35]. Hence, TFs expressed in multiple normal tissue types can be as important to the development of a specific cancer-type than TFs which are expressed only in the corresponding normal tissue type. In line with this, we have seen that a considerable number of TFs are overexpressed in many different tissue types and also seen to be silenced in common between cancer types. For instance, between lung, kidney, bladder and colon tissue, ten TFs (*CASZ1*, *NR3C2*, *THRA*, *SETBP1*, *SMARCA2*, *MEIS2*, *NFIC*, *PURA*, *KLF13*, *TCF21*) were commonly overexpressed in all these tissues compared with hESCs and also commonly silenced in LSCC, LUAD, KIRC, KIRP, BLCA and COAD compared with their respective normal tissues. This list includes known tumour suppressors such as the nuclear receptor *NR3C2* [69], the helix-loop-helix transcription factor *TCF21* [70], and *SMARCA2* (also known as *BRM*), a member of the SNF/SWI chromatin remodelling complex [71–73]. Interestingly, however, the list also includes *SETBP1*, a TF which has been reported to be oncogenic in myeloid neoplasms [74, 75], highlighting the need to explore a potential tumour suppressive role of this TF in the context of epithelial cancer.

## Conclusions

The data presented here support the view that bivalently and PRC2-marked TFs expressed in a given normal tissue are more likely to undergo silencing in the corresponding cancer type compared with other non-housekeeping genes that are highly expressed in the same normal tissue. This suggests that putative differentiation blocks arising as a result of their inactivation are strongly selected for during carcinogenesis. Importantly, our data suggest that the silencing of these TFs in cancer is predominantly associated with promoter hypermethylation.

## Additional file

Additional file 1: This document contains all Supplementary Tables and all Supplementary Figures, plus their associated legends/captions. (PDF 3658 kb)

## Abbreviations

BLCA: Bladder carcinoma
CNV: Copy number variation
COAD: Colon adenoma carcinoma
DNAm: DNA methylation
hESC: Human embryonic stem cell
KIRC: Kidney renal cell carcinoma
KIRP: Kidney renal papillary carcinoma
LSCC: Lung squamous cell carcinoma
LUAD: Lung adenoma carcinoma
miRNA: MicroRNA
PRC2: Polycomb repressive complex 2
SCM2: Stem Cell Matrix-2
STAD: Stomach adenocarcinoma
TCGA: The Cancer Genome Atlas
TF: Transcription factor
