# Supplementary material for: The multi-omic landscape of transcription factor inactivation in cancer
Source: Genome Med. 2016 Aug 25;8(1):89. doi: 10.1186/s13073-016-0342-8 (PMC4997779; doi:10.1186/s13073-016-0342-8)
Supplement: Additional file 1: — This document contains all Supplementary Tables and all Supplementary Figures, plus their associated legends/captions. (PDF 3658 kb) [file 13073_2016_342_MOESM1_ESM.pdf]

Additional File 1

for

The multi-omic landscape of transcription factor inactivation in cancer

Andrew E. Teschendorff [1,2,3,*] , Shijie C. Zheng [1] , Andy Feber [4], Zhen Yang [1] , Stephan Beck [4] and Martin Widschwendter [3]

*Corresponding author: Andrew E. Teschendorff- a.teschendorff@ucl.ac.uk

(1) CAS Key Laboratory of Computational Biology, CAS-MPG Partner Institute for Computational Biology, Chinese Academy of Sciences, Shanghai Institute for Biological Sciences, 320 Yue Yang Road, Shanghai 200031, China. (2) Statistical Cancer Genomics, Paul O'Gorman Building, UCL Cancer Institute, University College London, 72 Huntley Street, London WC1E 6BT, United Kingdom. (3) Department of Women's Cancer, University College London, 74 Huntley Street, London WC1E 6BT, United Kingdom. (4) Medical Genomics, Paul O'Gorman Building, UCL Cancer Institute, University College London, 72 Huntley Street, London WC1E 6BT, United Kingdom.

SUPPLEMENTARY FIGURES:

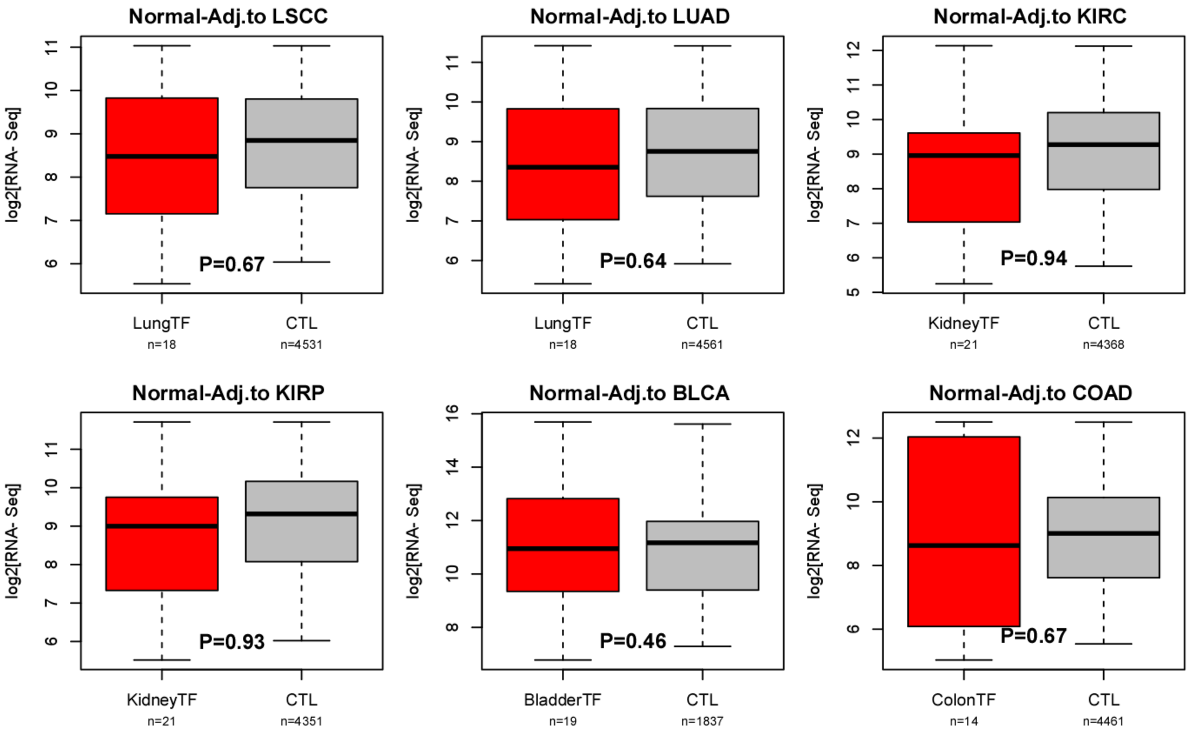

Figure S1: Definition of control set of genes. Boxplots comparing the log2-normalized gene expression levels of transcription factors (TFs, red boxes) overexpressed in only one of the 4 normal tissue types (lung-LungTF, kidney-KidneyTF, bladder-BladderTF and colon-ColonTF) in the normal-adjacent tissue of TCGA cancer types (LSCC, LUAD, KIRC, KIRP, BLCA and COAD) to the corresponding expression levels of a control set of genes (CTL, grey boxes), defined as non-housekeeping genes which exhibit the same level of gene expression in the normal-adjacent tissue than the identified TFs. P-values are from a one-tailed Wilcoxon rank sum test to check that the TFs do not exhibit higher levels of expression than the control genes.

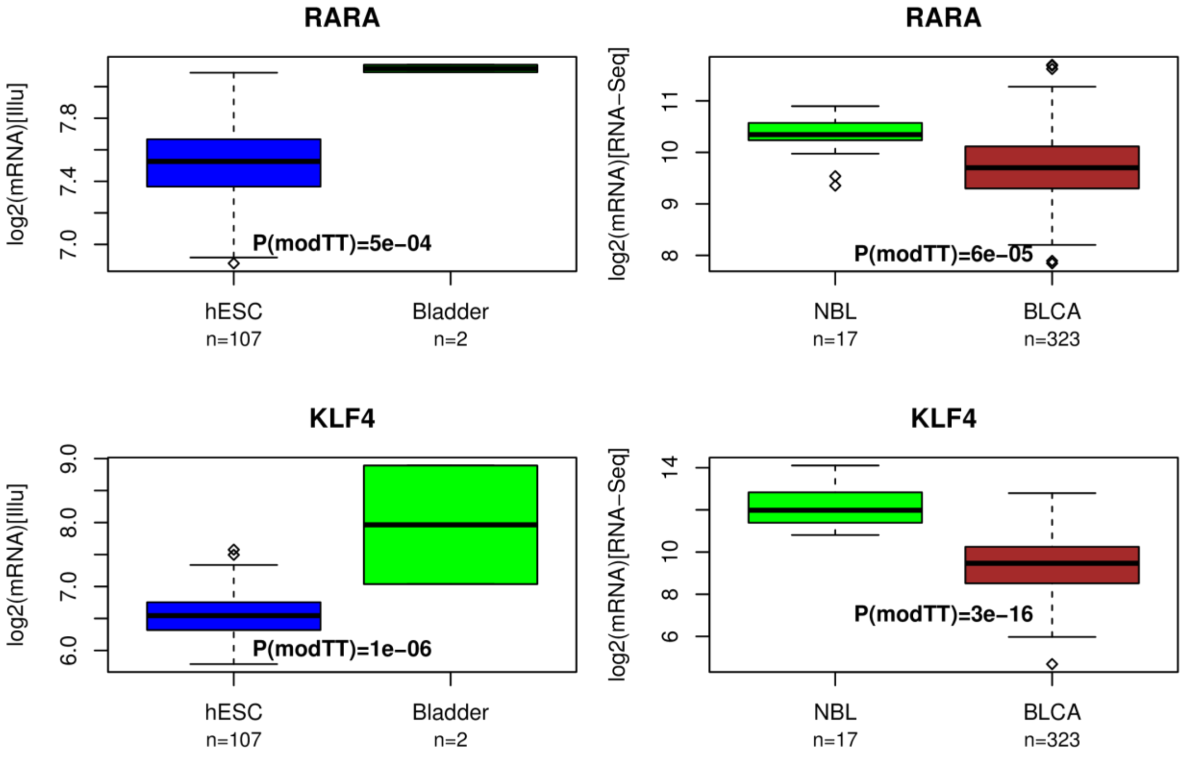

Figure S2: Examples of two known TFs implicated in urothelial cell differentiation. Shown are boxplots of the gene expression changes in hESCs compared to adult bladder tissue (left panels), and in adult normal bladder (NBL) compared to bladder cancer (BLCA). The number of samples in each category is indicated below the label. The P-values are from moderated t-tests as evaluated using the limma R-package. Observe how these two TFs (RARA and KLF4) are overexpressed in adult bladder tissue compared to hESCs, but then become significantly underexpressed in bladder cancer. Gene expression data for the left panels were generated on Illumina beadarrays, whereas data in the right panels were generated using RNA-Seq (TCGA level-3 data).

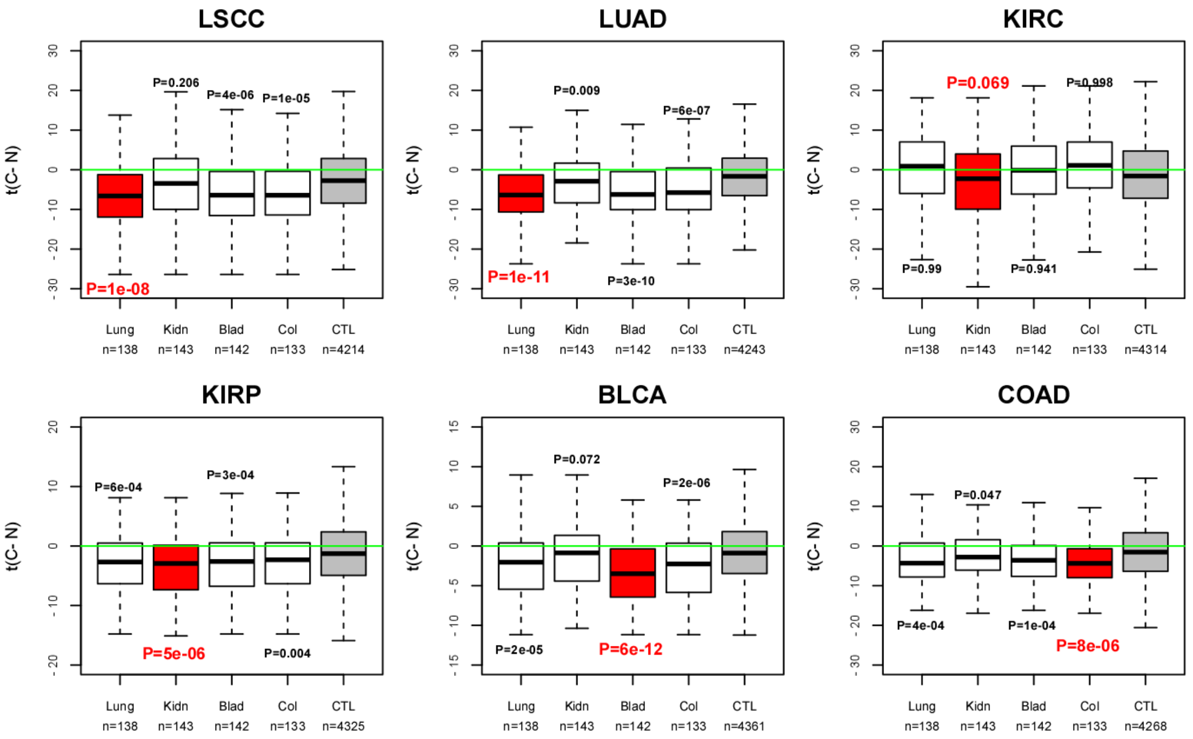

Figure S3: Transcription factors expressed in normal tissue are preferentially silenced in the corresponding cancer-type. Boxplots of t-statistics of differential mRNA expression between cancer and normal tissue for 5 sets of genes, across six different cancer types, as indicated. LSCC=lung squamous cell carcinoma, LUAD=lung adenoma carcinoma, KIRC=kidney renal carcinoma, KIRP=kidney renal papillary carcinoma, BLCA=bladder carcinoma and COAD=colon adenoma carcinoma. The 5 sets of genes are the transcription factors expressed in the relevant normal tissue (red box) (without restriction to being only expressed in that normal tissue type**),** the transcription factors expressed in the other normal tissue types (white boxes) and a set of control (CTL, grey box) non-housekeeping genes which are expressed at a similar level to the TFs expressed in that same normal tissue. We note that in this figure (as opposed to Fig.2A), there is overlap between the different TF groups. P-values are from a one-tailed Wilcoxon-rank sum test comparing the t-statistics of each group of TFs to the control (CTL) gene set.

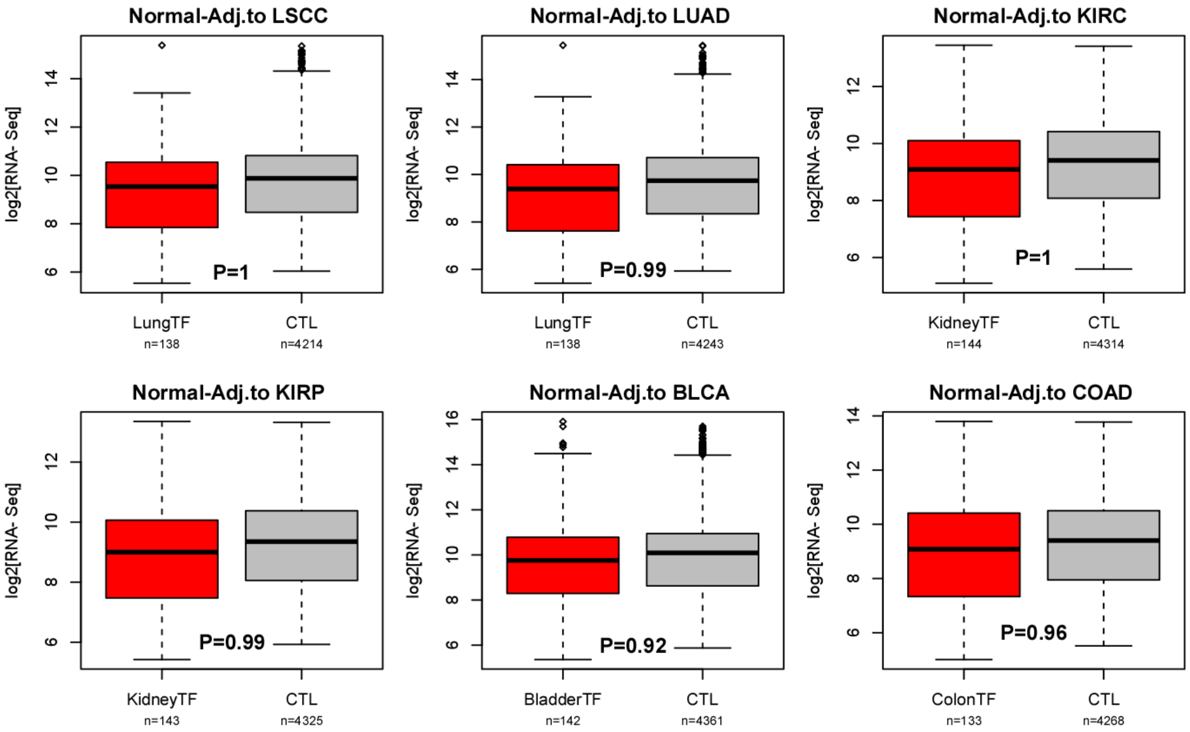

Figure S4: Definition of control set of genes. Boxplots comparing the log2-normalized gene expression levels of transcription factors (TFs, red boxes) overexpressed in each of the 4 normal tissue types (lung-LungTF, kidney-KidneyTF, bladder-BladderTF and colon-ColonTF) compared to hESCs, to the corresponding expression levels of a control set of genes (CTL, grey boxes), defined as non-housekeeping genes which exhibit the same level of gene expression in the normal-adjacent tissue than the identified TFs. P-values are from a one-tailed Wilcoxon rank sum test to check that the TFs do not exhibit higher levels of expression than the control genes.

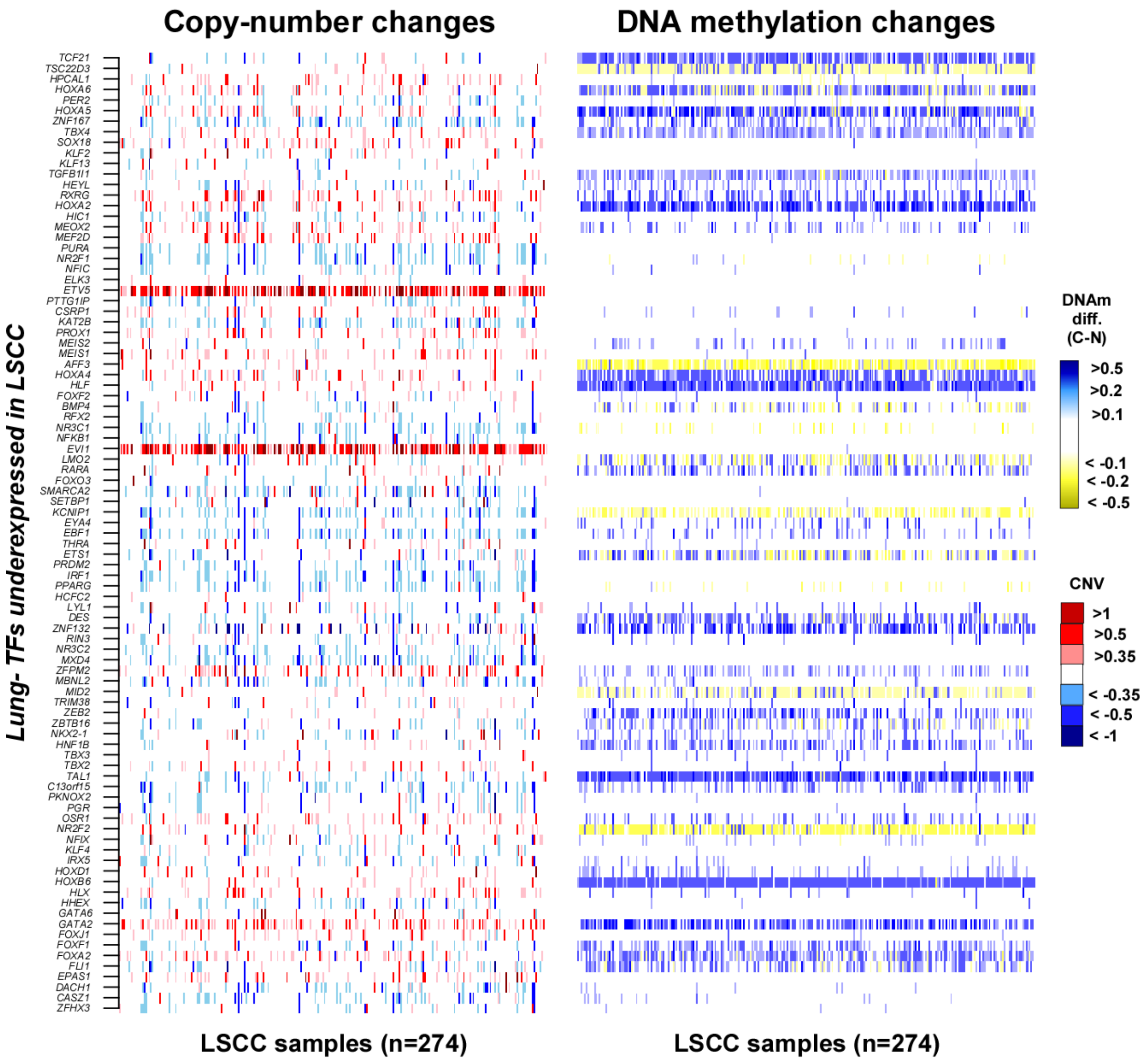

Figure S5: Heatmaps of copy-number and DNA methylation changes in lung squamous cell carcinomas (LSCC) for the lung-expressed transcription factors which are downregulated in LSCC. In the case of copy-number, the colors represent the segment values assigned to the corresponding genes, as indicated. In the case of DNA methylation, the colors indicate the difference in beta (DNAm) value between cancer and the average of all normal lung samples.

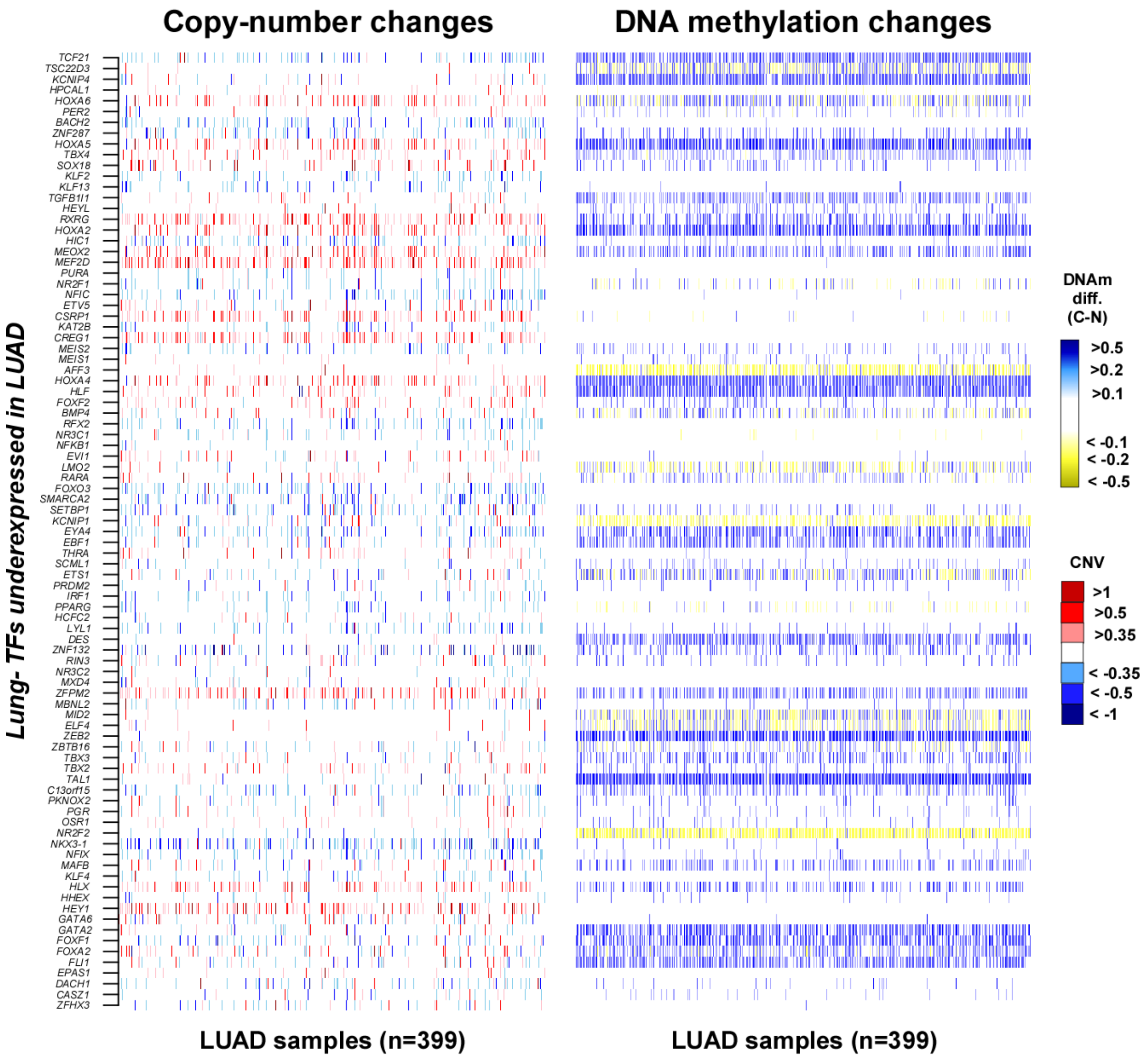

Figure S6: Heatmaps of copy-number and DNA methylation changes in lung adenocarcinomas (LUAD) for the lung-expressed transcription factors which are downregulated in LUAD. In the case of copy-number, the colors represent the segment values assigned to the corresponding genes, as indicated. In the case of DNA methylation, the colors indicate the difference in beta (DNAm) value between cancer and the average of all normal lung samples.

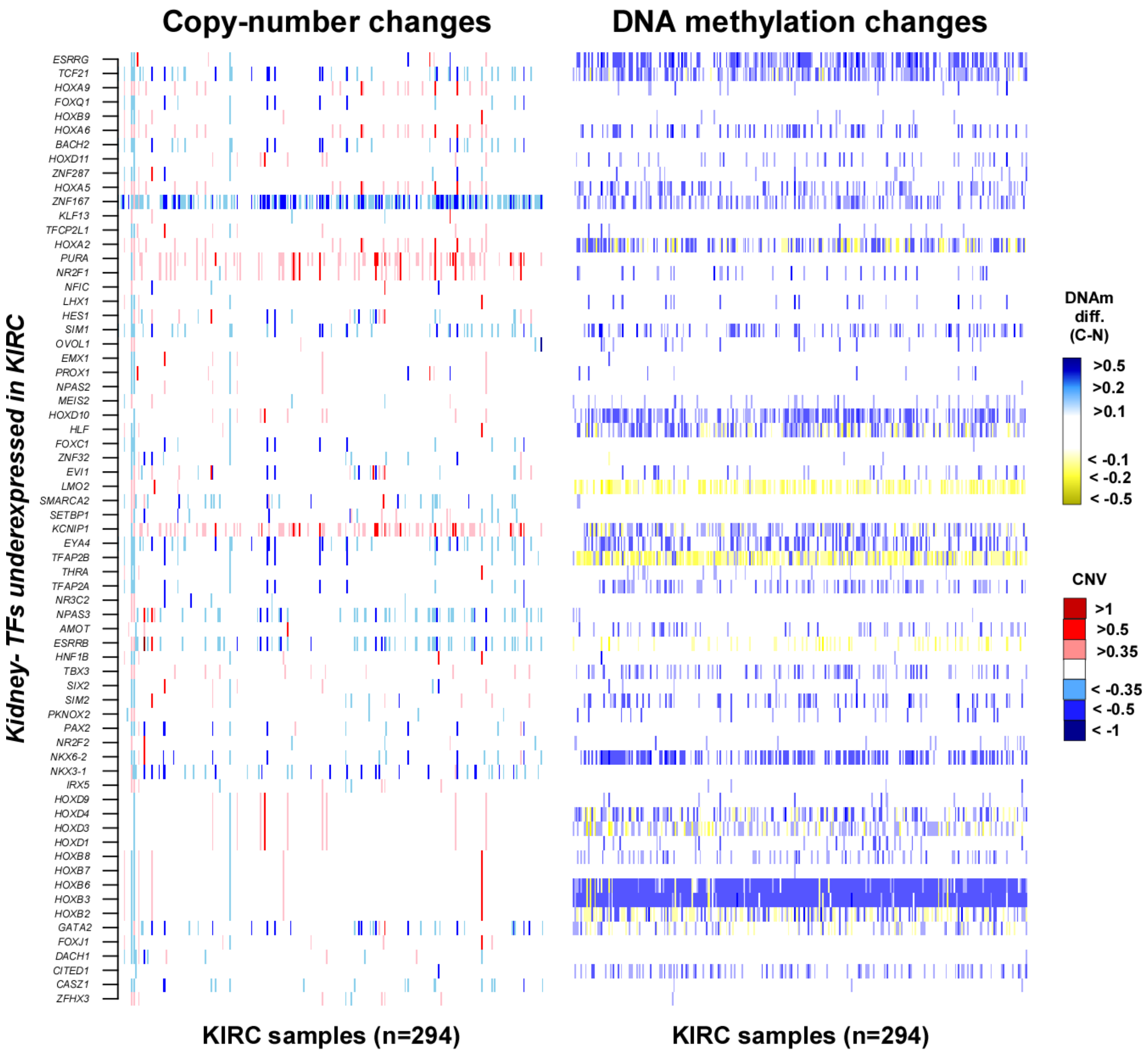

Figure S7: Heatmaps of copy-number and DNA methylation changes in kidney renal cell carcinoma (KIRC) for the kidney-expressed transcription factors which are downregulated in KIRC. In the case of copy-number, the colors represent the segment values assigned to the corresponding genes, as indicated. In the case of DNA methylation, the colors indicate the difference in beta (DNAm) value between cancer and the average of all normal kidney samples.

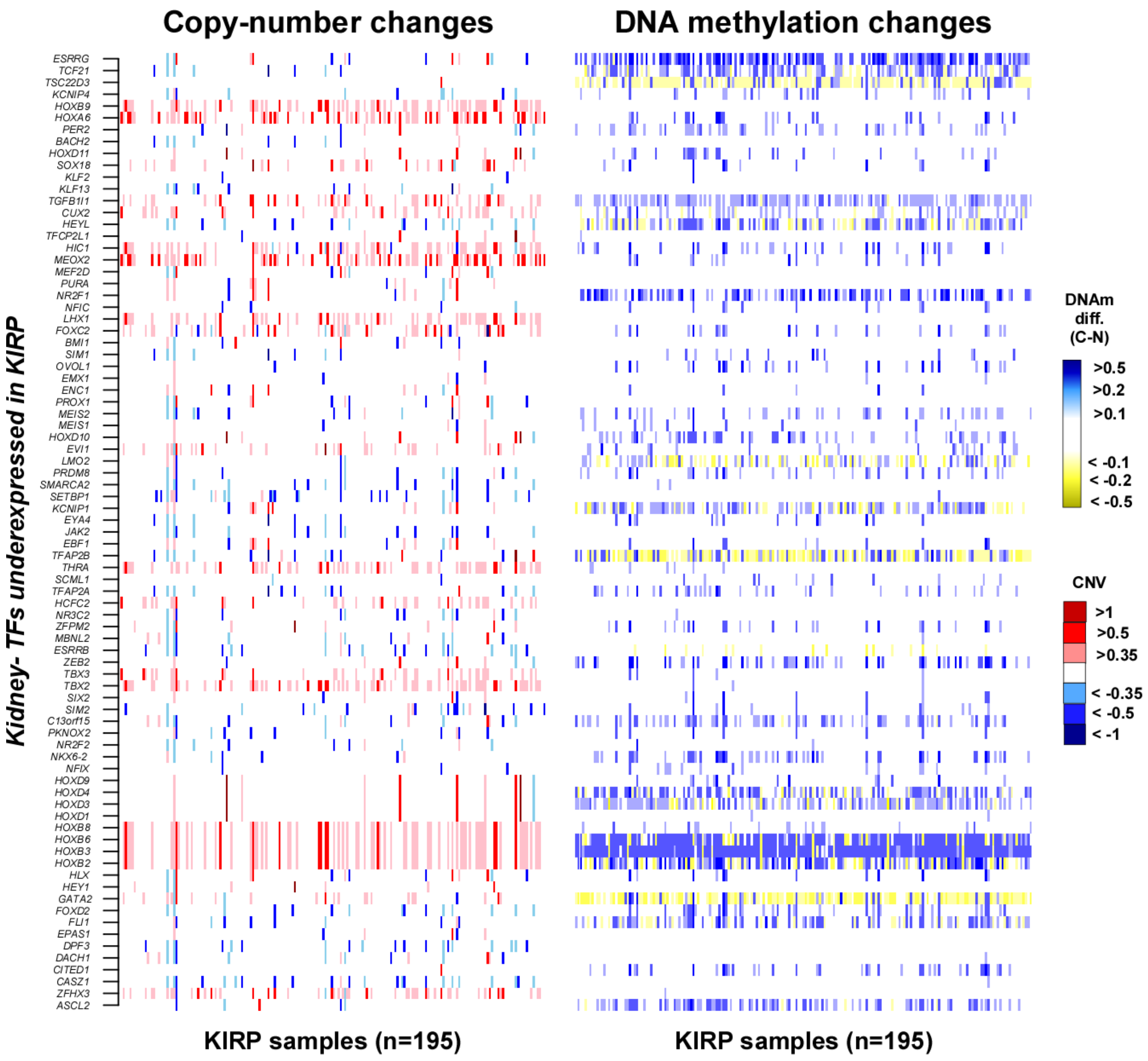

Figure S8: Heatmaps of copy-number and DNA methylation changes in kidney renal papillary cell carcinoma (KIRP) for the kidney-expressed transcription factors which are downregulated in KIRP. In the case of copy-number, the colors represent the segment values assigned to the corresponding genes, as indicated. In the case of DNA methylation, the colors indicate the difference in beta (DNAm) value between cancer and the average of all normal kidney samples.

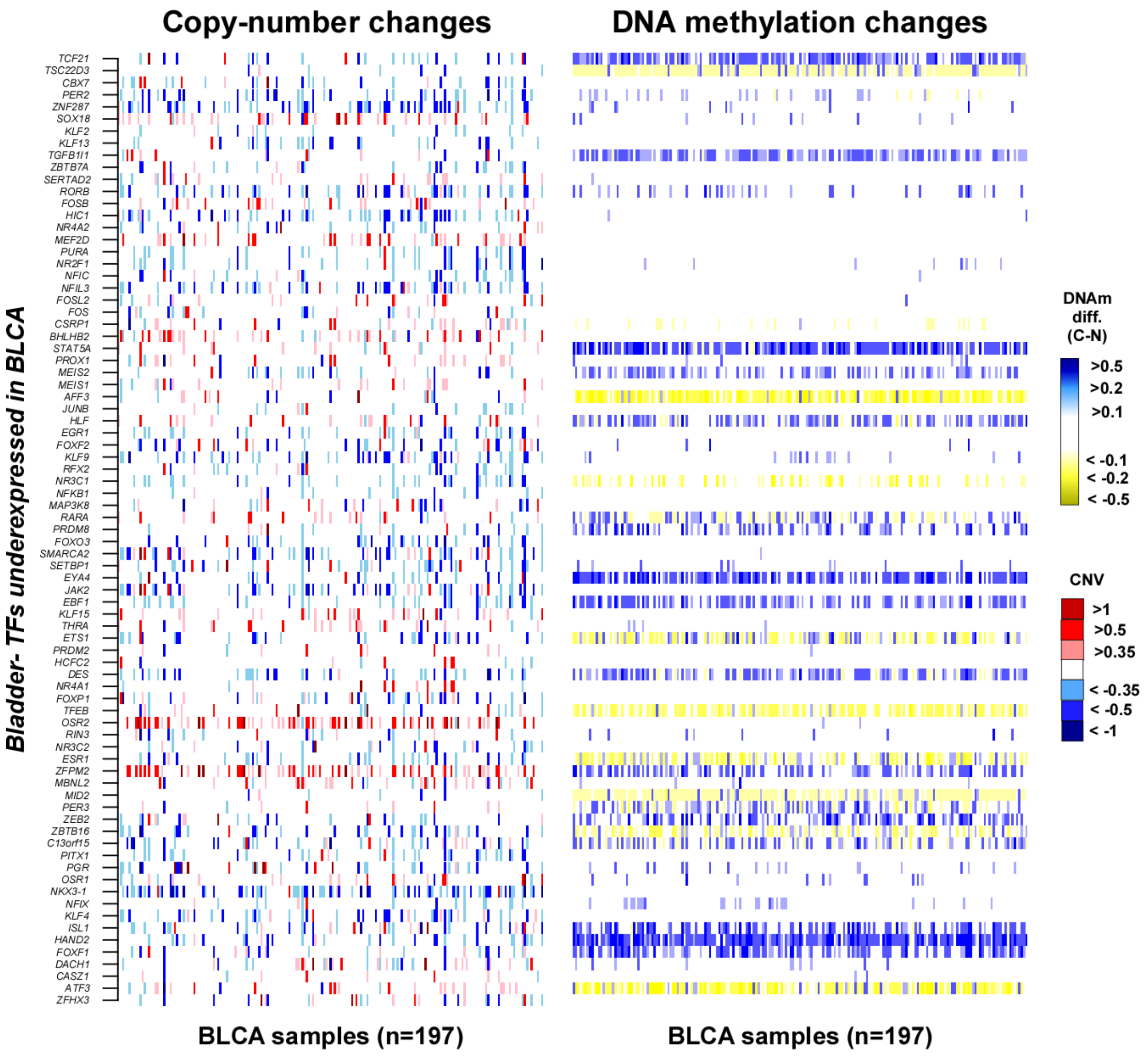

Figure S9: Heatmaps of copy-number and DNA methylation changes in bladder carcinoma (BLCA) for the bladder-expressed transcription factors which are downregulated in BLCA. In the case of copy-number, the colors represent the segment values assigned to the corresponding genes, as indicated. In the case of DNA methylation, the colors indicate the difference in beta (DNAm) value between cancer and the average of all normal bladder samples.

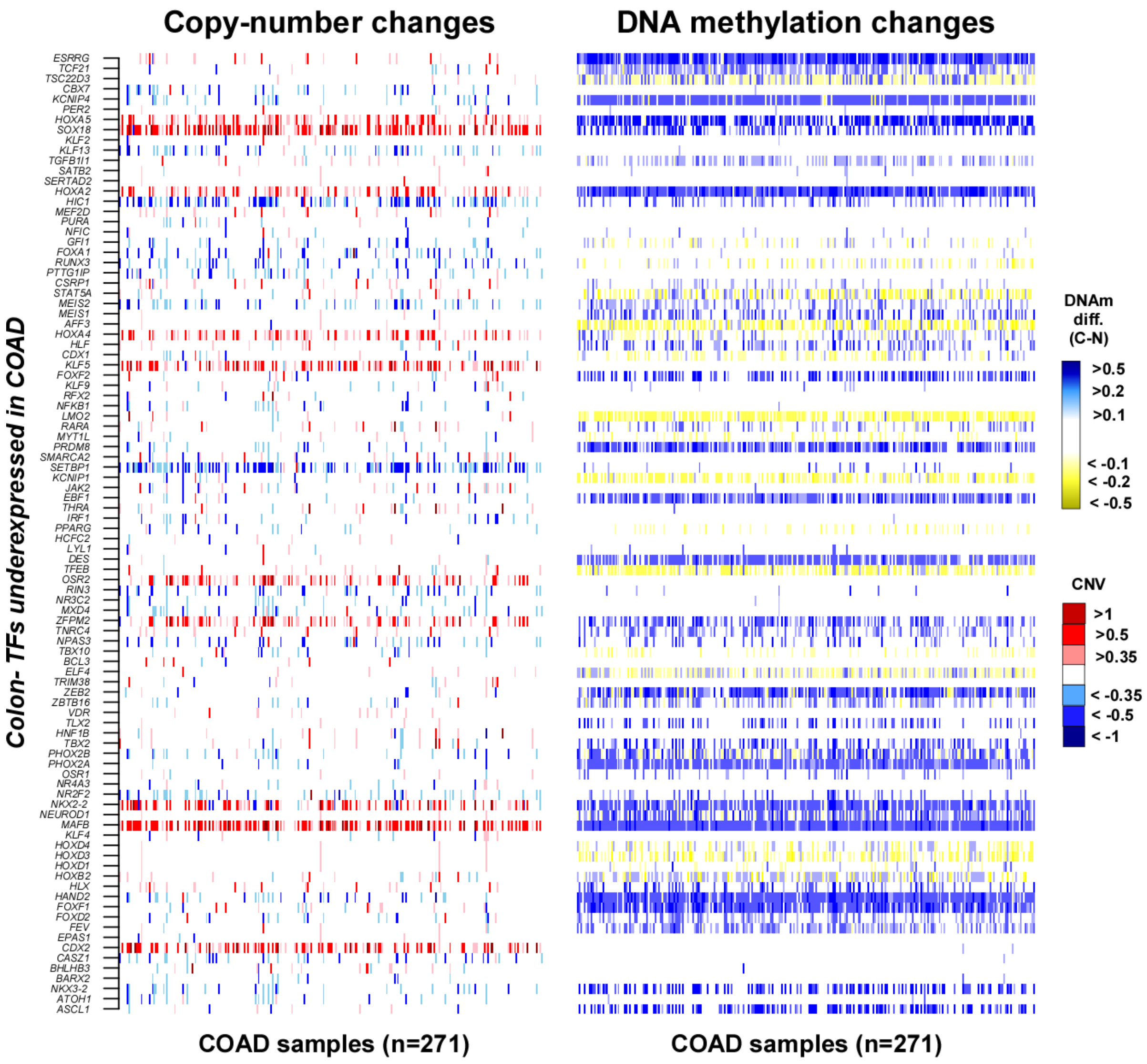

Figure S10: Heatmaps of copy-number and DNA methylation changes in colon adenoma carcinoma (COAD) for the colon-expressed transcription factors which are downregulated in COAD. In the case of copy-number, the colors represent the segment values assigned to the corresponding genes, as indicated. In the case of DNA methylation, the colors indicate the difference in beta (DNAm) value between cancer and the average of all normal colon samples.

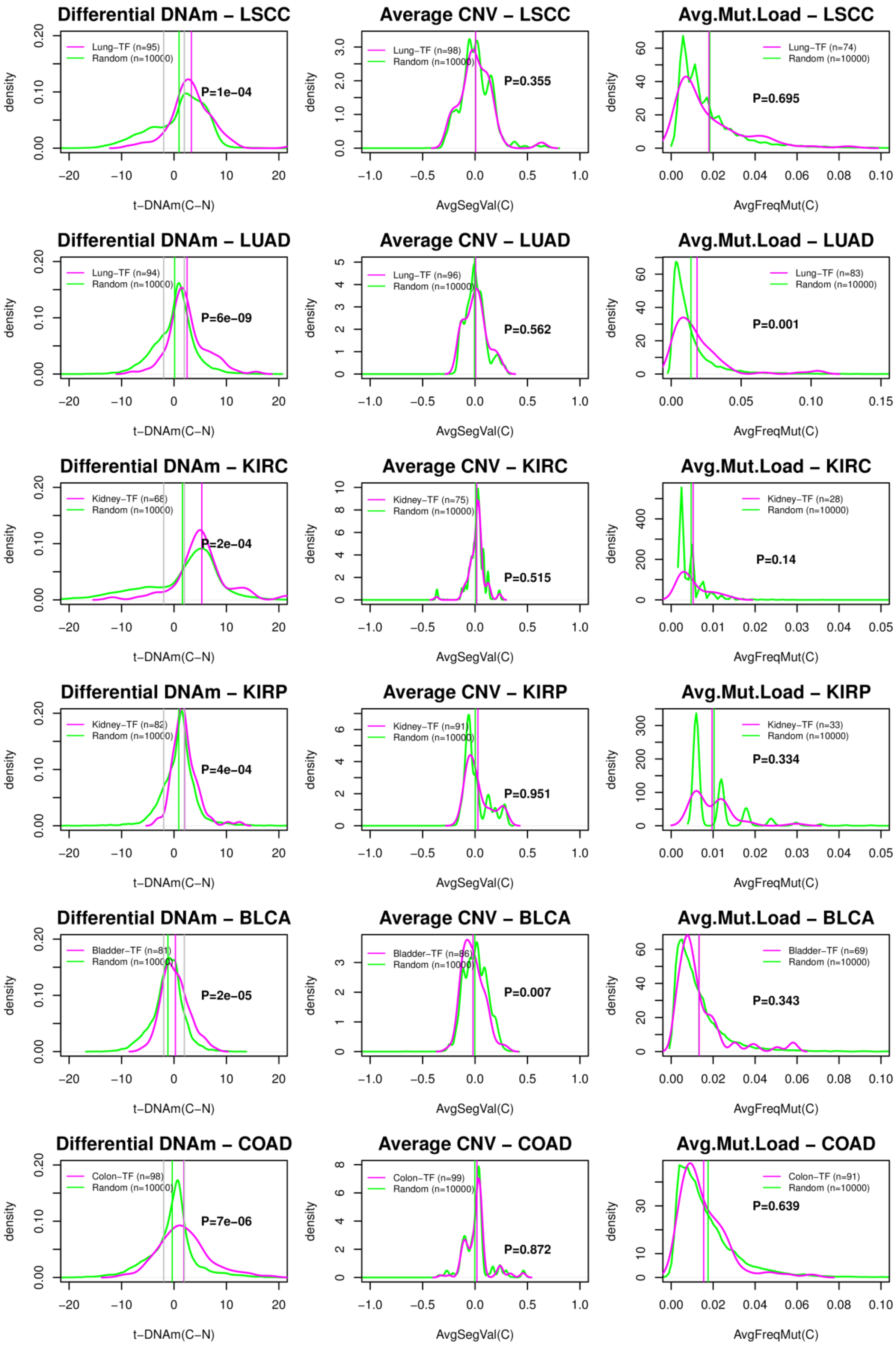

Differential DNAm – LSCC
Lung–TF (n=95)
Random (n=10000)
P=1e–04
t–DNAm(C–N)
density
Average CNV – LSCC
Lung–TF (n=98)
Random (n=10000)
P=0.355
AvgSegVal(C)
density
Avg.Mut.Load – LSCC
Lung–TF (n=74)
Random (n=10000)
P=0.695
AvgFreqMut(C)
density
Differential DNAm – LUAD
Lung–TF (n=94)
Random (n=10000)
P=6e–09
t–DNAm(C–N)
Average CNV – LUAD
Lung–TF (n=96)
Random (n=10000)
P=0.562
AvgSegVal(C)
Avg.Mut.Load – LUAD
Lung–TF (n=83)
Random (n=10000)
P=0.001
AvgFreqMut(C)
Differential DNAm – KIRC
Kidney–TF (n=68)
Random (n=10000)
P=2e–04
t–DNAm(C–N)
Average CNV – KIRC
Kidney–TF (n=75)
Random (n=10000)
P=0.515
AvgSegVal(C)
Avg.Mut.Load – KIRC
Kidney–TF (n=28)
Random (n=10000)
P=0.14
AvgFreqMut(C)
Differential DNAm – KIRP
Kidney–TF (n=82)
Random (n=10000)
P=4e–04
t–DNAm(C–N)
Average CNV – KIRP
Kidney–TF (n=91)
Random (n=10000)
P=0.951
AvgSegVal(C)
Avg.Mut.Load – KIRP
Kidney–TF (n=33)
Random (n=10000)
P=0.334
AvgFreqMut(C)
Differential DNAm – BLCA
Bladder–TF (n=81)
Random (n=10000)
P=2e–05
t–DNAm(C–N)
Average CNV – BLCA
Bladder–TF (n=86)
Random (n=10000)
P=0.007
AvgSegVal(C)
Avg.Mut.Load – BLCA
Bladder–TF (n=69)
Random (n=10000)
P=0.343
AvgFreqMut(C)
Differential DNAm – COAD
Colon–TF (n=98)
Random (n=10000)
P=7e–06
t–DNAm(C–N)
Average CNV – COAD
Colon–TF (n=99)
Random (n=10000)
P=0.872
AvgSegVal(C)
Avg.Mut.Load – COAD
Colon–TF (n=91)
Random (n=10000)
P=0.639
AvgFreqMut(C)

Figure S11: Left panels: density plots of t-statistics of differential DNA methylation between cancer and normal tissue [t(C-N)], of the cancer silenced TFs (magenta curves) compared to the corresponding density distribution of 10000 randomly selected genes (green curves). Density plots are shown for six cancer types LSCC, LUAD, KIRC, KIRP, BLCA and COAD. P-values are from a one-tailed Wilcoxon rank sum test. The vertical magenta and green lines denote the average levels. The grey vertical lines in the DNA methylation plot indicate the lines P=0.05. **Middle Panels:** as left panels, but for the average CNV segment values of the silenced TFs (magenta). **Right panels:** as left panels, but for the frequency of inactivating mutation of the silenced TFs.

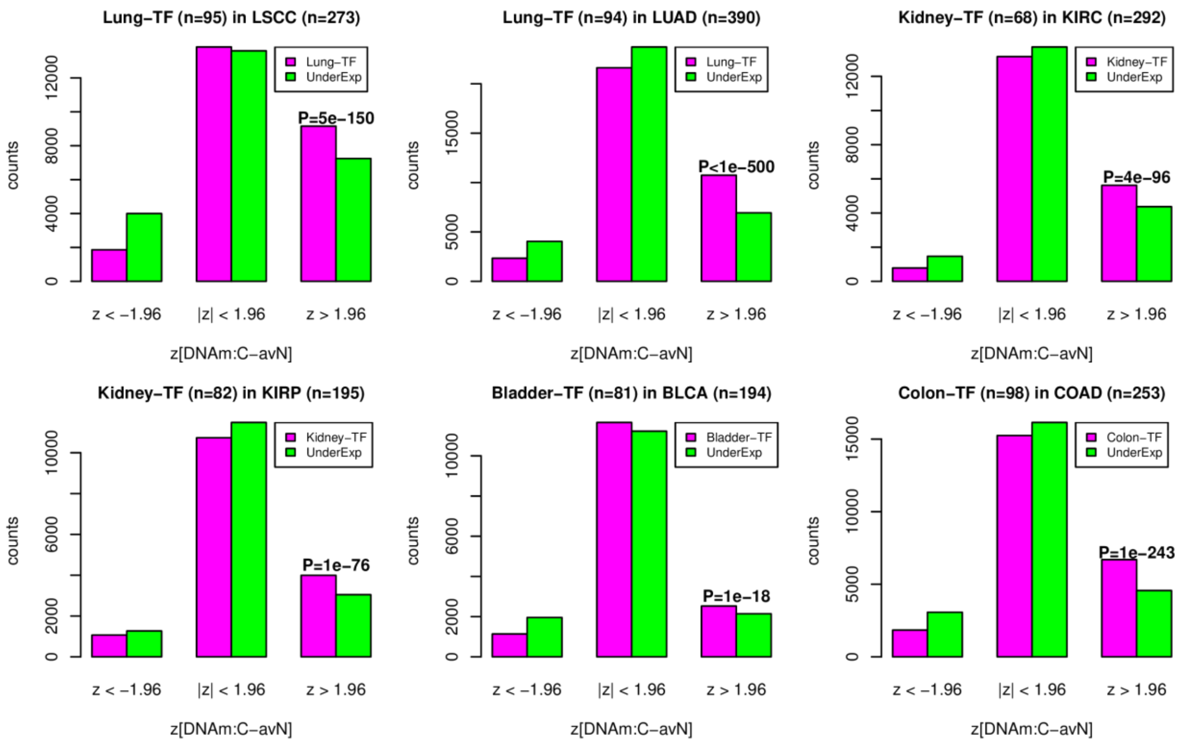

Figure S12: Barplots listing for each TCGA cancer type, the number of silenced TFs and tumours (y-axis, magenta-colored bars) exhibiting DNA methylation z-statistics of a particular magnitude, and compared to the corresponding expected numbers had this number of genes been drawn from the set of all cancer-underexpressed genes (y-axis, green colored bars). The z-statistic for a gene in a given tumour was computed as the difference between the DNAm level in the tumour and the average DNAm across the normals, divided by the standard deviation in DNAm across the normals. z-statistics were divided into 3 groups, based on (i) being smaller than -1.96 (corresponding to significant (P<0.05) hypomethylation in the tumour compared to normal, (ii) not being significant (P>0.05, |z| < 1.96), and (iii)

exhibiting significant hypermethylation in the tumour compared to normals (z > 1.96, P<0.05). The expected counts for the set of all cancer-underexpressed genes was obtained by computing the frequency of TF-tumour pairs in each z-statistic category and multiplying that frequency with the number of data points in the silenced-TF list. The P-value shown in each panel is derived from a Binomial-test, comparing the observed count for the silenced TF set to the expected count based on the set of all cancer-underexpressed genes.

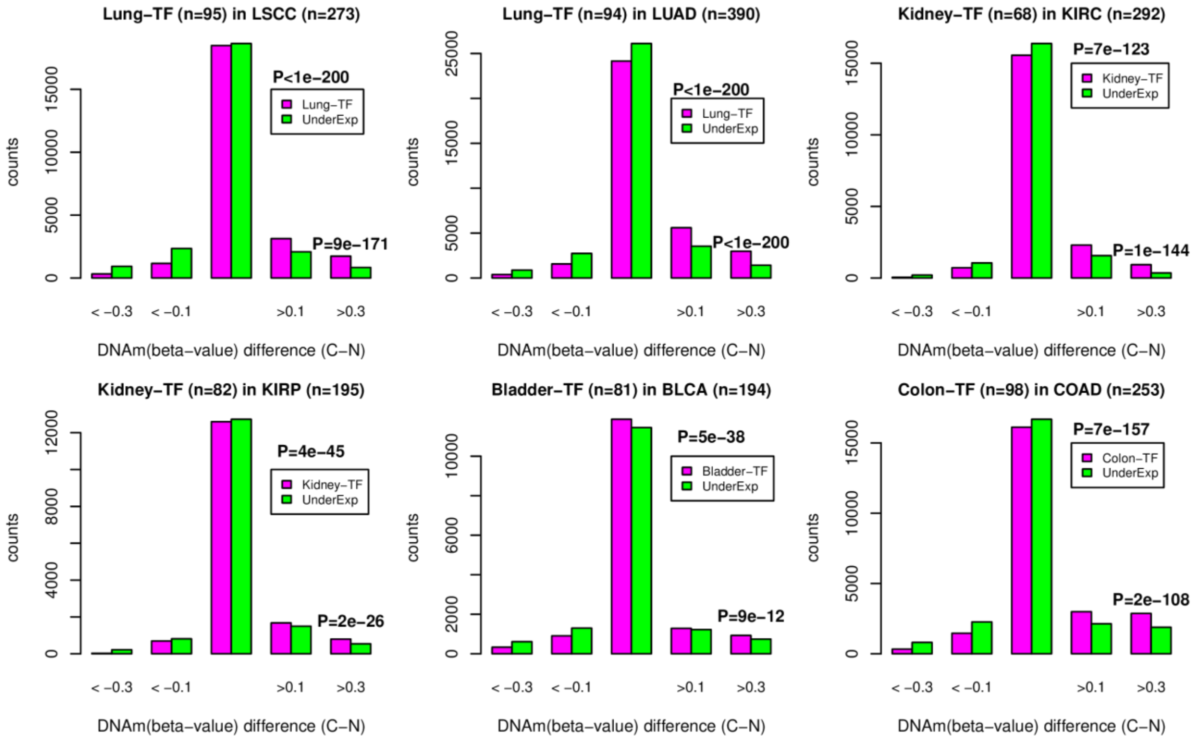

Figure S13: Barplots listing for each TCGA cancer type, the number of silenced TFs and tumours (y-axis, magenta-colored bars) exhibiting DNA methylation differences of a particular magnitude, and compared to the corresponding expected numbers had this number of genes been drawn from the set of all cancer-underexpressed genes (y-axis, green colored bars). Differences in DNAm were estimated for each tumour compared to the average over the normals and stratified into 5 groups: (i) $\Delta\beta < -0.3$, (ii) $\Delta\beta < -0.1$, (iii) $|\Delta\beta| < 0.1$, (iv) $\Delta\beta > 0.1$ and (v) $\Delta\beta > 0.3$, with positive values indicating hypermethylation in cancer. The expected counts for the set of all cancer-underexpressed genes was obtained by computing the frequency of TF-tumour pairs in each $\Delta\beta$ category and multiplying that frequency with the number of data points in the silenced-TF list. Two P-values are shown in each panel: the one above the legend derives from a Chi-Square test comparing the counts of the two sets of

genes across the five categories. The P-value shown for category “$\Delta\beta > 0.3$” is derived from a Binomial-test, comparing the observed count for the silenced TF set to the expected count based on the set of all cancer-underexpressed genes.

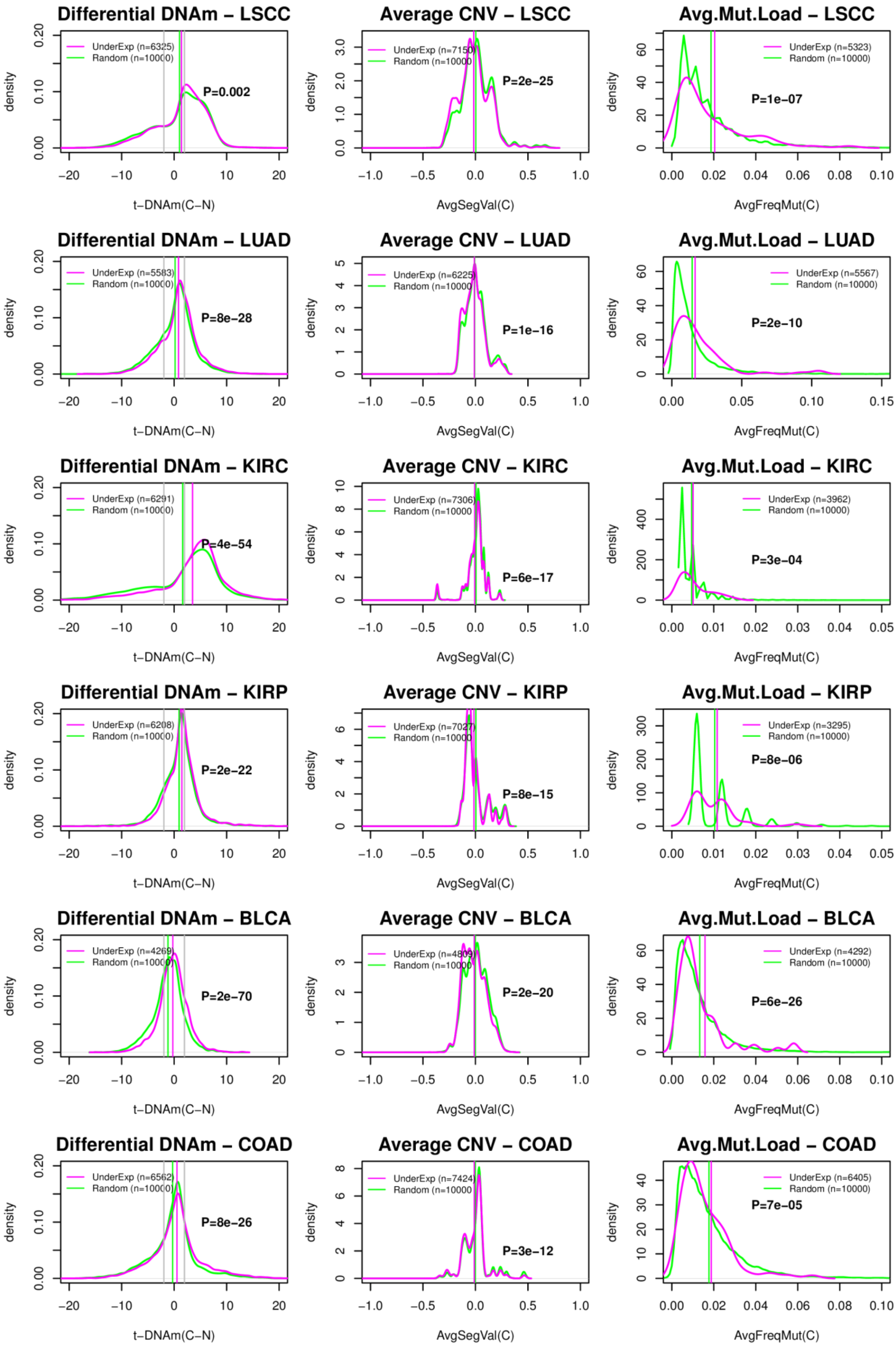
Differential DNAm – LSCC
UnderExp (n=6325)
Random (n=10000)
P=0.002
t–DNAm(C–N)
density
Average CNV – LSCC
UnderExp (n=7150)
Random (n=10000)
P=2e–25
AvgSegVal(C)
Avg.Mut.Load – LSCC
UnderExp (n=5323)
Random (n=10000)
P=1e–07
AvgFreqMut(C)
Differential DNAm – LUAD
UnderExp (n=5583)
Random (n=10000)
P=8e–28
Average CNV – LUAD
UnderExp (n=6225)
Random (n=10000)
P=1e–16
Avg.Mut.Load – LUAD
UnderExp (n=5567)
Random (n=10000)
P=2e–10
Differential DNAm – KIRC
UnderExp (n=6291)
Random (n=10000)
P=4e–54
Average CNV – KIRC
UnderExp (n=7306)
Random (n=10000)
P=6e–17
Avg.Mut.Load – KIRC
UnderExp (n=3962)
Random (n=10000)
P=3e–04
Differential DNAm – KIRP
UnderExp (n=6208)
Random (n=10000)
P=2e–22
Average CNV – KIRP
UnderExp (n=7027)
Random (n=10000)
P=8e–15
Avg.Mut.Load – KIRP
UnderExp (n=3295)
Random (n=10000)
P=8e–06
Differential DNAm – BLCA
UnderExp (n=4269)
Random (n=10000)
P=2e–70
Average CNV – BLCA
UnderExp (n=4809)
Random (n=10000)
P=2e–20
Avg.Mut.Load – BLCA
UnderExp (n=4292)
Random (n=10000)
P=6e–26
Differential DNAm – COAD
UnderExp (n=6562)
Random (n=10000)
P=8e–26
Average CNV – COAD
UnderExp (n=7424)
Random (n=10000)
P=3e–12
Avg.Mut.Load – COAD
UnderExp (n=6405)
Random (n=10000)
P=7e–05

Figure S14: Left panels: density plots of t-statistics of differential DNA methylation between cancer and normal tissue [t(C-N)] of all genes under-expressed in cancer (magenta curves) compared to the corresponding density distribution of 10000 randomly selected genes. Density plots are shown for six cancer types LSCC, LUAD, KIRC, KIRP, BLCA and COAD. P-values are from a one-tailed Wilcoxon rank sum test. The vertical magenta and green lines denote the average levels. The grey vertical lines in the DNA methylation plot indicate the lines P=0.05. **Middle Panels:** as left panels, but for the average CNV segment values. **Right panels:** as left panels, but for the frequency of inactivating mutation.

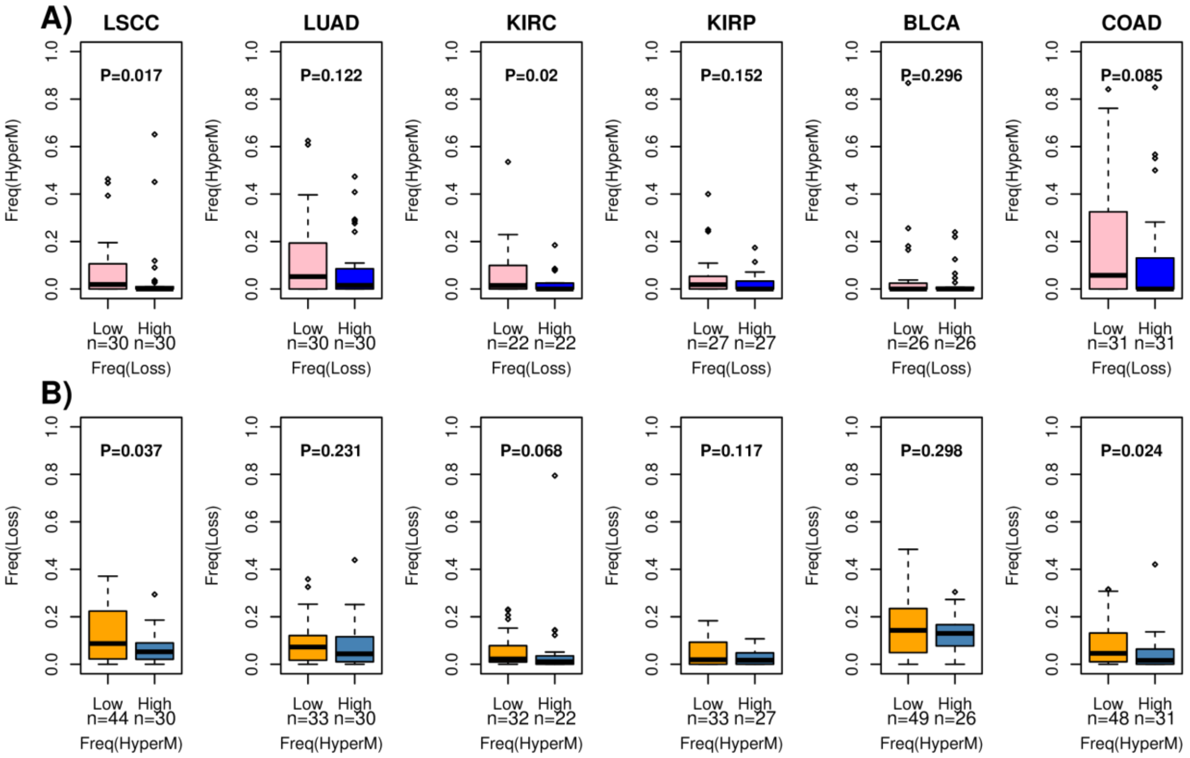

Figure S15: A) Boxplots comparing the frequency of promoter hypermethylation of TFs (y-axis) that are in the lowest frequency tertile of TFs undergoing CNV loss ("Low") to those in the highest frequency tertile ("High") (x-axis). Number of TFs in each group is given underneath each box. For each TF, frequencies of alteration are estimated only over tumors exhibiting underexpression of the TF, and all TFs considered are expressed in the relevant normal tissue as well as being underexpressed in the corresponding cancer-type (LSCC, LUAD, KIRC, KIRP, BLCA and COAD). P-values are from a one-tailed Wilcox-rank sum test. Combined Fisher test P-value over all 6 cancer types is 0.002. **B)** As A), but with the roles of CNV loss and promoter hypermethylation reversed. We note that in the case of promoter hypermethylation, the "Low" class contains all (and only) TFs with a zero

frequency of hypermethylation across all tumours (using a stringent delta-beta threshold of > +/- 0.3 to call a promoter hypermethylated in cancer compared to normal), which is why numbers are not matched to the "High" category. Combined Fisher test P-value over all 6 cancer types is 0.004.

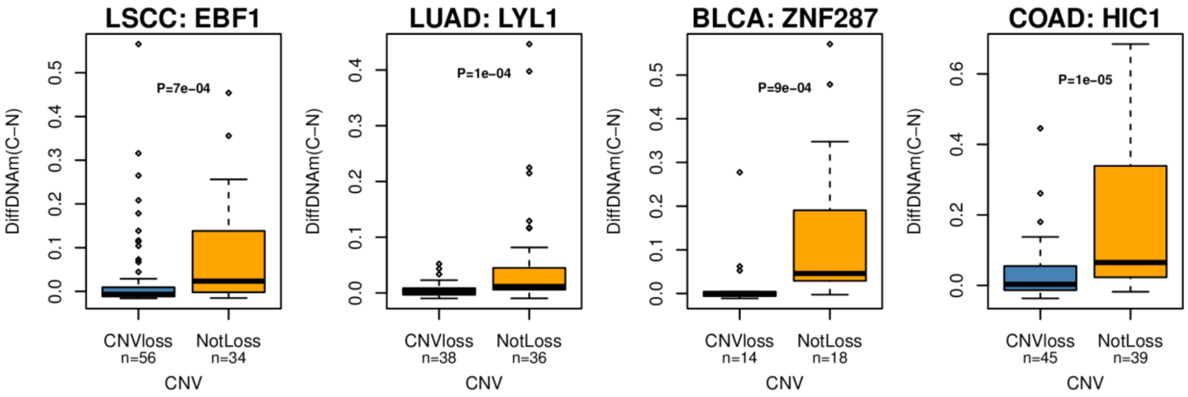

Figure S16: Some of the very few examples of cancer silenced TFs undergoing at least 10% frequency of both promoter hypermethylation and CNV loss, and for which there is evidence of mutual exclusivity in the sense of an observed higher promoter hypermethylation among tumours not exhibiting a CNV loss. P-values are from a Wilcoxon-rank sum test. Numbers of tumours exhibiting underexpression of the given TF in the given cancer type and which have (CNVloss) or don't have a CNV loss (NotLoss) at that locus are given below each boxplot.

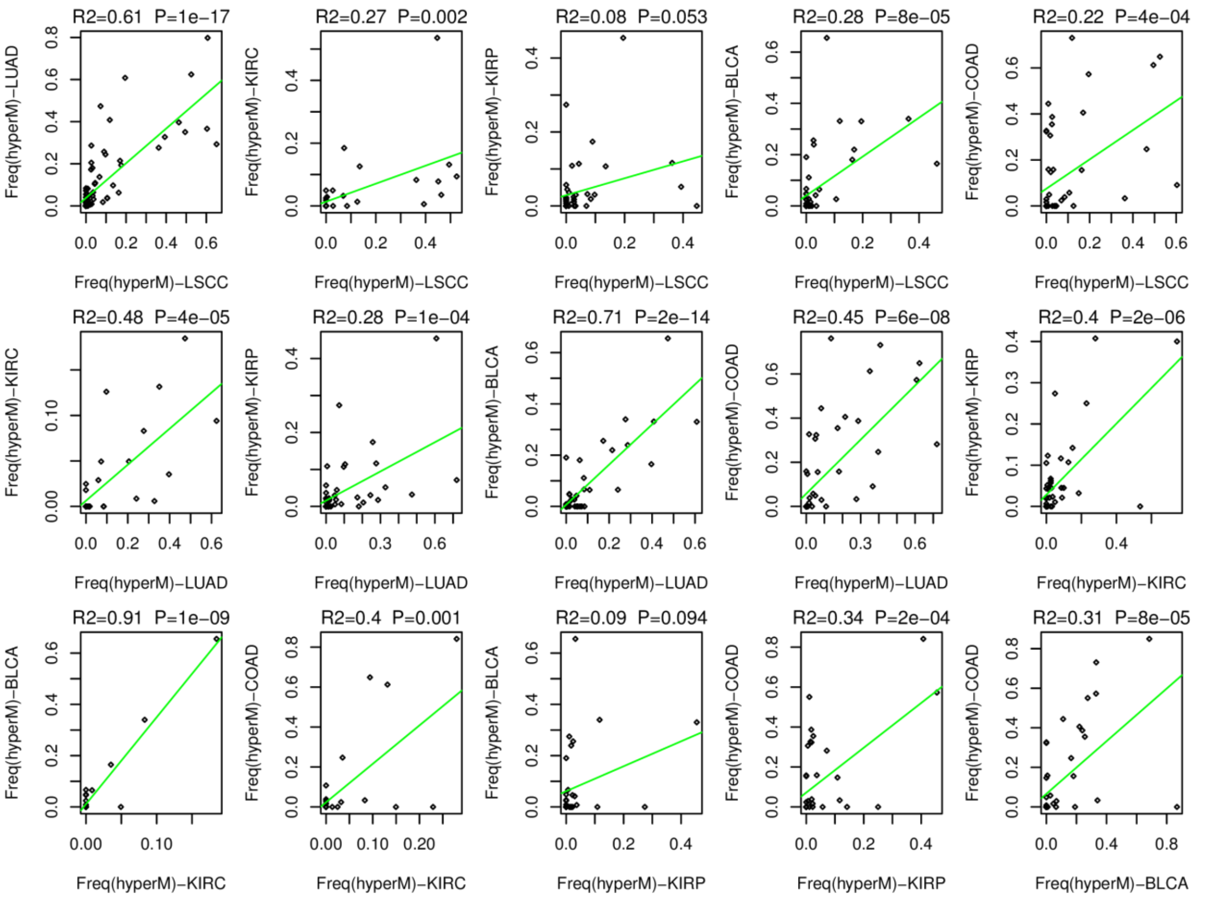

Figure S17: Scatterplots of the frequency of promoter DNA hypermethylation in cancer compared to normal, between every pair of cancer types, for all TFs commonly silenced in the two cancer types. Green line represents the linear least squares estimate and the R^2 (R2) and P-value given is from this linear regression.

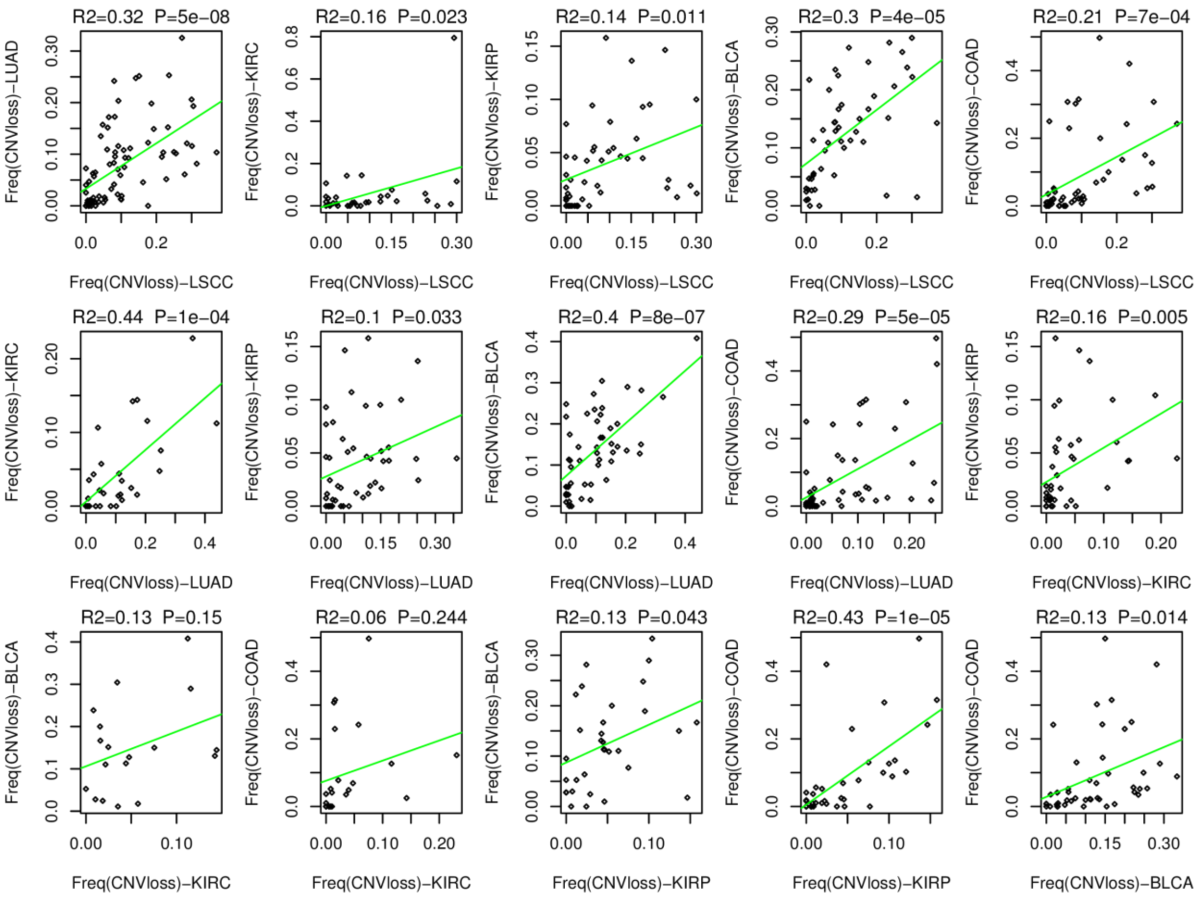

Figure S18: Scatterplots of the frequency of genomic (CNV) loss in cancer, between every pair of cancer types, for all TFs commonly silenced in the two cancer types. Green line represents a linear least squares estimate and the R^2 (R2) and P-value given is from this linear regression.

SUPPLEMENTARY TABLES:

Bivalent and PRC2 marked TFs in hESCs present in SCM2 expression data set	
**Entrez Gene ID**	**Gene Symbol**
429	ASCL1
430	ASCL2
463	ZFHX3

467	ATF3
474	ATOH1
579	NKX3-2
56033	BARX1
8538	BARX2
79365	BHLHB3
646	BNC1
54897	CASZ1
57332	CBX8
1045	CDX2
4435	CITED1
1602	DACH1
1746	DLX2
8110	DPF3
1960	EGR3
1961	EGR4
2019	EN1
2020	EN2
2034	EPAS1
54738	FEV
2313	FLI1
3170	FOXA2
2306	FOXD2
27022	FOXD3
2304	FOXE1
2294	FOXF1
2290	FOXG1
2302	FOXJ1
668	FOXL2
2624	GATA2
2625	GATA3
2626	GATA4
2627	GATA6
2637	GBX2
9464	HAND2
54626	HES2
23462	HEY1
3087	HHEX
3142	HLX
3211	HOXB1
3212	HOXB2
3213	HOXB3
3216	HOXB6
3217	HOXB7
3218	HOXB8
3227	HOXC11
3221	HOXC4
3222	HOXC5
3223	HOXC6
3224	HOXC8
3231	HOXD1
3238	HOXD12
3239	HOXD13
3232	HOXD3

3233	HOXD4
3235	HOXD9
3299	HSF4
50805	IRX4
10265	IRX5
3670	ISL1
3725	JUN
9314	KLF4
10660	LBX1
9355	LHX2
64211	LHX5
26468	LHX6
9935	MAFB
9242	MSC
4487	MSX1
4618	MYF6
4654	MYOD1
4760	NEUROD1
4761	NEUROD2
63973	NEUROG2
4784	NFIX
4821	NKX2-2
26257	NKX2-8
4824	NKX3-1
84504	NKX6-2
4861	NPAS1
7026	NR2F2
8013	NR4A3
10215	OLIG2
3175	ONECUT1
9480	ONECUT2
130497	OSR1
5076	PAX2
5080	PAX6
5081	PAX7
7849	PAX8
5083	PAX9
5241	PGR
401	PHOX2A
8929	PHOX2B
5307	PITX1
5308	PITX2
5309	PITX3
63876	PKNOX2
5453	POU3F1
5457	POU4F1
5458	POU4F2
30062	RAX
28984	C13orf15
6474	SHOX2
6493	SIM2
6495	SIX1
10736	SIX2
6496	SIX3

4990	SIX6
55509	BATF3
8403	SOX14
64321	SOX17
6886	TAL1
10716	TBR1
6899	TBX1
6909	TBX2
30009	TBX21
6926	TBX3
6928	HNF1B
7080	NKX2-1
3196	TLX2
114088	TRIM9
25806	VAX2
7421	VDR
7490	WT1
7704	ZBTB16
9839	ZEB2
7545	ZIC1
84107	ZIC4
55079	FEZF2
22806	IKZF3
11317	RBPJL
8863	PER3
63976	PRDM16
633	BGN
26038	CHD5
5452	POU2F2
27033	ZBTB32
10475	TRIM38
7593	MZF1
2000	ELF4
3208	HPCA
2103	ESRRB
4661	MYT1
6939	TCF15
602	BCL3
347853	TBX10
11043	MID2
89870	TRIM15
154796	AMOT
3200	HOXA3
3206	HOXA10
64067	NPAS3
2334	AFF2
11189	TNRC4
4793	NFKBIB
7539	ZFP37
406	ARNTL
2100	ESR2
10150	MBNL2
23414	ZFPM2
367	AR

10608	MXD4
2099	ESR1
51176	LEF1
9330	GTF3C3
9112	MTA1
4306	NR3C2
4602	MYB
79890	RIN3
55734	ZFP64
3229	HOXC13
55663	ZNF446
83463	MXD3
7691	ZNF132
116039	OSR2
6935	ZEB1
1396	CRIP1
7942	TFEB
7553	ZNF7
23241	PACS2
27086	FOXP1
8216	LZTR1
10848	PPP1R13L
54796	BNC2
79776	ZFHX4
7101	NR2E1
3164	NR4A1
1674	DES
2078	ERG
30819	KCNIP2
54760	PCSK4
3663	IRF5
1820	ARID3A
56270	WDR45L
51042	ZNF593
4066	LYL1
4791	NFKB2
6015	RING1
80758	PRR7
54583	EGLN1
29915	HCFC2
5468	PPARG
23493	HEY2
3659	IRF1
4610	MYCL1
51085	MLXIPL
2139	EYA2
7799	PRDM2
8535	CBX4
2118	ETV4
2972	BRF1
2113	ETS1
7020	TFAP2A
6322	SCML1
23774	BRD1

8019	BRD3
2516	NR5A1
4601	MXI1
7067	THRA
1959	EGR2
28999	KLF15
7021	TFAP2B
1879	EBF1
3717	JAK2
2070	EYA4
6596	HLTF
30820	KCNIP1
56731	SLC2A4RG
9219	MTA2
1870	E2F2
26040	SETBP1
1050	CEBPA
23030	JMJD2B
1523	CUX1
6595	SMARCA2
2309	FOXO3
1750	DLX6
56978	PRDM8
23040	MYT1L
5914	RARA
4005	LMO2
7005	TEAD3
23119	HIC2
2122	EVI1
1397	CRIP2
1326	MAP3K8
861	RUNX1
3662	IRF4
4790	NFKB1
2908	NR3C1
6662	SOX9
326	AIRE
7291	TWIST1
190	NR0B1
5990	RFX2
3762	KCNJ5
1107	CHD3
7580	ZNF32
652	BMP4
687	KLF9
1628	DBP
2273	FHL1
2274	FHL2
2295	FOXF2
2296	FOXC1
688	KLF5
1044	CDX1
1958	EGR1
3131	HLF

3201	HOXA4
3215	HOXB5
3236	HOXD10
3642	INSM1
3726	JUNB
3899	AFF3
4004	LMO1
4097	MAFG
4208	MEF2C
4211	MEIS1
4212	MEIS2
4488	MSX2
4862	NPAS2
5328	PLAU
5629	PROX1
6618	SNAPC2
6722	SRF
6776	STAT5A
6839	SUV39H1
6862	T
6932	TCF7
7022	TFAP2C
8433	UTF1
8462	KLF11
8507	ENC1
8553	BHLHB2
8804	CREG1
8850	KAT2B
8928	FOXH1
1465	CSRP1
2016	EMX1
2018	EMX2
8328	GFI1B
754	PTTG1IP
860	RUNX2
864	RUNX3
1482	NKX2-5
2119	ETV5
3169	FOXA1
5017	OVOL1
9421	HAND1
6478	SIAH2
6492	SIM1
648	BMI1
1046	CDX4
1749	DLX5
2004	ELK3
2303	FOXC2
2353	FOS
2355	FOSL2
2672	GFI1
3149	HMGB3
4783	NFIL3
3207	HOXA11

3280	HES1
3975	LHX1
4617	MYF5
4782	NFIC
5454	POU3F2
6658	SOX3
7025	NR2F1
10138	YAF2
5813	PURA
4092	SMAD7
4209	MEF2D
4223	MEOX2
4772	NFATC1
4929	NR4A2
7745	ZNF192
7746	ZNF193
3090	HIC1
10661	KLF1
2354	FOSB
3199	HOXA2
5396	PRRX1
6096	RORB
6258	RXRG
6663	SOX10
7391	USF1
6772	STAT1
26574	AATF
23764	MAFF
23413	FREQ
27287	VENTX
7775	ZNF232
29842	TFCP2L1
8022	LHX3
26508	HEYL
9757	MLL4
9792	SERTAD2
22882	ZHX2
23237	ARC
23314	SATB2
23316	CUX2
51341	ZBTB7A
7041	TGFB1I1
51621	KLF13
51111	SUV420H1
51127	TRIM17
10365	KLF2
51450	PRRX2
3226	HOXC10
55922	NKRF
54345	SOX18
9496	TBX4
55888	ZNF167
3202	HOXA5
7148	TNXB

57336	ZNF287
3237	HOXD11
58495	OVOL2
59336	PRDM13
9572	NR1D1
60468	BACH2
8864	PER2
3203	HOXA6
3219	HOXB9
79943	ZNF696
94234	FOXQ1
3241	HPCAL1
80333	KCNIP4
92822	ZNF276
3205	HOXA9
23492	CBX7
7275	TUB
9586	CREB5
1831	TSC22D3
6943	TCF21
2104	ESRRG
571	BACH1

Table S1: This table lists all transcription factors (TFs), as defined by the Molecular Signatures Database (*www.broadinstitute.org/gsea/msigb*), which are also bivalently or PRC2 marked in hESCs [1,2], and which are present in the Stem Cell Matrix-2 (SCM2) Compendium expression data set [3,4].

Entrez GID	Symbol	t	P	P-adj	AvExpESC	AvExpTIS	logFC
429	ASCL1	8.98	9E-15	1E-13	5.95	9.94	3.98
430	ASCL2	2.05	0.042	0.042	6.54	7.12	0.58
463	ZFHX3	7.48	2E-11	2E-10	6.82	10.88	4.06
467	ATF3	-5.15	0.000001	0.000004	7.57	5.9	-1.67
579	NKX3-2	4.15	0.00007	0.0002	5.85	8.92	3.07
54897	CASZ1	4.59	0.00001	0.00004	6.69	7.75	1.06
57332	CBX8	4.17	0.00006	0.0002	5.97	6.58	0.61
1602	DACH1	6.34	5E-09	0.00000003	6.2	8.42	2.22
1960	EGR3	-3.13	0.002	0.004	6.34	5.64	-0.7
2034	EPAS1	4.91	0.000003	0.00001	7.64	12.18	4.54
2313	FLI1	3.17	0.002	0.003	5.3	5.86	0.56
3170	FOXA2	3.63	0.0004	0.0009	7.13	11.82	4.69
2306	FOXD2	2.87	0.005	0.007	5.41	5.89	0.48
27022	FOXD3	-4.52	0.00002	0.00005	6.73	5.33	-1.4
2294	FOXF1	12.1	6E-22	2E-20	5.76	9.58	3.81
2302	FOXJ1	2.42	0.017	0.02	5.69	6.29	0.59
2624	GATA2	2.94	0.004	0.006	5.54	6.37	0.83
2627	GATA6	4.38	0.00003	0.00008	5.62	6.55	0.92
23462	HEY1	2.28	0.025	0.027	6.98	8.03	1.05
3087	HHEX	3.58	0.0005	0.001	6.46	8.25	1.79

3142	HLX	10.61	2E-18	3E-17	5.95	8.8	2.85
3211	HOXB1	-3.09	0.003	0.004	6.75	6.05	-0.71
3212	HOXB2	4.22	0.00005	0.0001	6.89	10.92	4.03
3213	HOXB3	14.37	5E-27	2E-25	5.37	8.4	3.03
3216	HOXB6	2.37	0.019	0.022	6.71	7.31	0.59
3217	HOXB7	6.31	6E-09	0.00000004	5.87	9.36	3.48
3227	HOXC11	-2.15	0.033	0.034	6.86	6.09	-0.77
3221	HOXC4	8.1	8E-13	9E-12	5.65	7.63	1.98
3231	HOXD1	11.76	4E-21	8E-20	5.5	7.89	2.39
50805	IRX4	-2.6	0.01	0.014	6.4	5.31	-1.1
10265	IRX5	8.82	2E-14	2E-13	6.04	9.55	3.51
9314	KLF4	2.21	0.029	0.031	6.56	7.13	0.57
26468	LHX6	3.91	0.0002	0.0004	7.25	8.25	1
9935	MAFB	4.51	0.00002	0.00005	7.34	9.91	2.57
4784	NFIX	5.52	0.0000002	0.000001	6.95	12.24	5.29
4821	NKX2-2	8.31	3E-13	3E-12	5.49	6.52	1.04
4824	NKX3-1	2.16	0.033	0.034	6.04	6.68	0.64
84504	NKX6-2	-2.74	0.007	0.01	5.64	5.1	-0.54
4861	NPAS1	-7.21	7E-11	6E-10	8.87	6.2	-2.67
7026	NR2F2	6.98	2E-10	0.000000002	6.3	9.32	3.02
9480	ONECUT2	-2.88	0.005	0.007	5.8	5.42	-0.38
130497	OSR1	10.14	2E-17	3E-16	5.89	11.4	5.52
5076	PAX2	-3.07	0.003	0.004	5.39	5.04	-0.36
5081	PAX7	-2.34	0.021	0.024	8.3	7.78	-0.53
7849	PAX8	2.35	0.021	0.024	5.47	5.71	0.24
5241	PGR	5.62	0.0000001	0.0000007	5.29	6	0.71
5308	PITX2	-2.31	0.023	0.025	6.31	5.3	-1.01
63876	PKNOX2	4.48	0.00002	0.00005	6.52	8.43	1.92
5453	POU3F1	-2.7	0.008	0.011	6.74	5.41	-1.33
28984	C13orf15	8.25	4E-13	4E-12	7.27	13.09	5.82
6886	TAL1	3.21	0.002	0.003	5.65	6.2	0.55
6899	TBX1	6.43	3E-09	0.00000002	5.36	6.14	0.77
6909	TBX2	9.81	1E-16	2E-15	5.73	11.37	5.64
6926	TBX3	5.54	0.0000002	0.0000009	6.22	7.92	1.71
6928	HNF1B	5.73	0.00000009	0.0000004	5.55	6.92	1.37
7080	NKX2-1	63.09	3E-88	7E-85	5.24	9.73	4.49
7704	ZBTB16	3.43	0.0009	0.002	5.46	6.26	0.8
9839	ZEB2	7.77	4E-12	4E-11	6.36	10.44	4.07
63976	PRDM16	14.79	7E-28	3E-26	5.35	8.39	3.04
633	BGN	2.86	0.005	0.007	10.44	14.18	3.75
26038	CHD5	-2.61	0.01	0.013	6.86	5.47	-1.4
10475	TRIM38	4.62	0.00001	0.00003	5.98	7.32	1.35
7593	MZF1	2.6	0.011	0.014	7.64	8.03	0.39
2000	ELF4	2.18	0.032	0.033	7.81	8.4	0.59
11043	MID2	3.13	0.002	0.004	6.52	7.51	0.99
89870	TRIM15	-3.26	0.001	0.003	5.51	5.11	-0.4
3200	HOXA3	18.23	5E-35	3E-33	5.29	7.3	2.02
3206	HOXA10	-2.24	0.027	0.029	5.39	5.1	-0.29
2334	AFF2	2.5	0.014	0.017	6.32	7.04	0.73
4793	NFKBIB	-5.79	0.00000007	0.0000003	6.78	5.83	-0.95
7539	ZFP37	2.33	0.022	0.025	8.2	9.02	0.82
10150	MBNL2	7.95	2E-12	2E-11	6.06	8.85	2.78
23414	ZFPM2	10	4E-17	7E-16	5.5	8.05	2.56
10608	MXD4	8.26	4E-13	4E-12	10.15	12.25	2.1

51176	LEF1	3.06	0.003	0.004	7.14	9.74	2.6
9330	GTF3C3	-3.03	0.003	0.005	10.18	9.5	-0.68
4306	NR3C2	8.04	1E-12	1E-11	6.38	7.8	1.42
79890	RIN3	4.03	0.0001	0.0003	5.33	5.94	0.61
55734	ZFP64	-2.17	0.032	0.033	6.92	6.23	-0.69
55663	ZNF446	4.29	0.00004	0.0001	7.35	8.76	1.41
83463	MXD3	7.82	3E-12	3E-11	7.19	9.12	1.94
7691	ZNF132	2.01	0.047	0.045	5.79	6.21	0.42
6935	ZEB1	3.7	0.0003	0.0007	5.59	6.64	1.05
1396	CRIP1	6.43	3E-09	0.00000002	7.53	11.06	3.53
8216	LZTR1	4.5	0.00002	0.00005	9.97	11.02	1.05
10848	PPP1R13L	-2.73	0.007	0.01	7.4	6.47	-0.93
1674	DES	4.65	0.000009	0.00003	5.49	6.2	0.71
54760	PCSK4	-2.15	0.034	0.035	6.31	5.84	-0.48
1820	ARID3A	-2.79	0.006	0.009	11.23	10.2	-1.03
56270	WDR45L	-3.32	0.001	0.002	9.95	9.05	-0.91
51042	ZNF593	-3.12	0.002	0.004	10.03	9.34	-0.69
4066	LYL1	9.06	5E-15	8E-14	6.89	10.17	3.28
6015	RING1	5.85	0.00000005	0.0000003	10.87	12.24	1.37
80758	PRR7	-4.55	0.00001	0.00004	7.08	5.92	-1.16
29915	HCFC2	4.99	0.000002	0.000008	5.89	6.86	0.98
5468	PPARG	3.93	0.0002	0.0003	5.5	6.11	0.61
23493	HEY2	-6.54	2E-09	0.00000001	10.53	6.54	-3.99
3659	IRF1	4.43	0.00002	0.00006	7.73	9.16	1.43
51085	MLXIPL	-2.44	0.016	0.019	6.48	5.71	-0.77
2139	EYA2	2.99	0.003	0.005	6.95	8.99	2.04
7799	PRDM2	2.49	0.014	0.017	5.76	6.08	0.32
8535	CBX4	3.78	0.0003	0.0006	6.37	7.6	1.23
2118	ETV4	-3.93	0.0002	0.0004	8.64	6.86	-1.78
2972	BRF1	2.39	0.018	0.021	6.46	7.14	0.69
2113	ETS1	2.17	0.032	0.033	10.47	11.64	1.18
6322	SCML1	3.52	0.0006	0.001	6.46	7.39	0.93
23774	BRD1	2.51	0.014	0.017	7.61	8.32	0.71
7067	THRA	7.2	8E-11	6E-10	6.3	8.29	1.99
1959	EGR2	-2.3	0.023	0.026	7.6	5.98	-1.62
1879	EBF1	9.04	6E-15	8E-14	5.81	8.21	2.41
2070	EYA4	14.32	7E-27	3E-25	5.36	6.43	1.07
30820	KCNIP1	6.06	0.00000002	0.0000001	5.6	6.95	1.35
56731	SLC2A4RG	3.57	0.0005	0.001	7.36	8.23	0.87
1870	E2F2	2.3	0.023	0.026	8.85	10.4	1.55
26040	SETBP1	4.19	0.00006	0.0001	7.78	9.88	2.1
1050	CEBPA	3.9	0.0002	0.0004	6.85	9.13	2.28
1523	CUX1	2.17	0.032	0.034	6.77	7.1	0.33
6595	SMARCA2	6.36	5E-09	0.00000003	8.99	11.32	2.33
2309	FOXO3	4.57	0.00001	0.00004	10.35	11.71	1.36
5914	RARA	4.56	0.00001	0.00004	7.51	8.28	0.77
4005	LMO2	8.77	3E-14	3E-13	6.66	9.26	2.6
23119	HIC2	-3.51	0.0006	0.001	10.78	9.71	-1.07
2122	EVI1	16.51	2E-31	8E-30	5.97	11.51	5.54
1397	CRIP2	7.42	3E-11	2E-10	7.09	11.42	4.33
861	RUNX1	2.38	0.019	0.022	5.55	6.18	0.63
4790	NFKB1	6.9	4E-10	0.000000003	9.27	12.05	2.77
2908	NR3C1	4.54	0.00001	0.00004	5.89	6.52	0.63
6662	SOX9	6.24	8E-09	0.00000005	7.34	10.73	3.39

7291	TWIST1	-2.11	0.037	0.037	8.52	7.19	-1.33
5990	RFX2	5.47	0.0000003	0.000001	5.52	6.62	1.11
7580	ZNF32	4.57	0.00001	0.00004	7.92	8.73	0.81
652	BMP4	2.6	0.011	0.014	6.57	7.71	1.14
1628	DBP	-2.71	0.008	0.01	6.57	5.62	-0.95
2273	FHL1	2.9	0.004	0.007	8.01	9.33	1.32
2274	FHL2	-6.87	4E-10	0.000000003	9.05	7.77	-1.27
2295	FOXF2	21.57	3E-41	2E-39	5.63	10.21	4.58
1958	EGR1	-2.22	0.029	0.031	11.13	9.95	-1.19
3131	HLF	3.11	0.002	0.004	5.46	5.88	0.42
3201	HOXA4	40.18	1E-67	1E-64	5.25	9.5	4.25
3215	HOXB5	5.34	0.0000005	0.000002	7.04	12.8	5.76
3899	AFF3	6.1	0.00000002	0.00000009	6.23	9.31	3.08
4097	MAFG	-4.15	0.00006	0.0002	8	6.82	-1.17
4208	MEF2C	16.29	4E-31	2E-29	5.41	7.98	2.57
4211	MEIS1	8.68	4E-14	5E-13	6.31	11.08	4.77
4212	MEIS2	4.01	0.0001	0.0003	6.26	7.89	1.63
4862	NPAS2	6.32	6E-09	0.00000003	5.89	6.92	1.03
5328	PLAU	-4.28	0.00004	0.0001	9.58	6.27	-3.31
5629	PROX1	6.5	2E-09	0.00000002	5.53	6.79	1.26
7022	TFAP2C	-7.03	2E-10	0.000000001	9.82	6.92	-2.91
8433	UTF1	-3.38	0.001	0.002	7.38	5.35	-2.03
8507	ENC1	4.66	0.000009	0.00003	8.41	11.14	2.73
8553	BHLHB2	-2.43	0.017	0.02	9.21	7.27	-1.94
8804	CREG1	5.35	0.0000005	0.000002	9.17	10.23	1.06
8850	KAT2B	2.2	0.03	0.032	5.39	5.62	0.23
8928	FOXH1	-2.15	0.034	0.035	6.94	6.48	-0.47
1465	CSRP1	2.51	0.014	0.017	8.63	11.76	3.13
754	PTTG1IP	2.43	0.017	0.02	11.37	11.84	0.47
2119	ETV5	3.77	0.0003	0.0006	8.19	10.22	2.03
3169	FOXA1	6.77	7E-10	0.000000005	6.26	11.41	5.15
648	BMI1	6.35	5E-09	0.00000003	8.6	10.89	2.3
1749	DLX5	2.78	0.006	0.009	6.3	9.35	3.05
2004	ELK3	5.05	0.000002	0.000007	5.72	6.75	1.03
2672	GFI1	2.99	0.003	0.005	5.35	5.71	0.36
3149	HMGB3	-7.05	2E-10	0.000000001	11.33	9.43	-1.9
3280	HES1	3.01	0.003	0.005	7.32	8.96	1.64
4782	NFIC	6.36	5E-09	0.00000003	5.62	6.59	0.97
6658	SOX3	-7.15	1E-10	8E-10	8.94	5.4	-3.55
7025	NR2F1	8.9	1E-14	2E-13	5.81	10.67	4.85
10138	YAF2	2.47	0.015	0.018	6.86	7.49	0.64
5813	PURA	3.03	0.003	0.005	6.42	7.17	0.76
4209	MEF2D	3.75	0.0003	0.0006	8.4	9.32	0.92
4223	MEOX2	35.06	2E-61	6E-59	5.23	9.03	3.81
7745	ZNF192	2.26	0.026	0.028	5.78	6.26	0.48
3090	HIC1	4.88	0.000004	0.00001	5.97	6.91	0.94
2354	FOSB	-2.1	0.038	0.038	9.57	6.84	-2.73
3199	HOXA2	11.99	1E-21	3E-20	5.72	9.98	4.25
6258	RXRG	14.68	1E-27	4E-26	5.33	6.55	1.22
6663	SOX10	-2.45	0.016	0.019	6.36	5.59	-0.77
26574	AATF	-2.94	0.004	0.006	9.86	9.04	-0.82
27287	VENTX	-6.54	2E-09	0.00000001	8.44	5.78	-2.66
26508	HEYL	7.11	1E-10	0.000000001	7.37	10.97	3.6
9792	SERTAD2	4.62	0.00001	0.00003	9.92	11.43	1.51

23237	ARC	-2.14	0.035	0.036	6.25	5.47	-0.78
23316	CUX2	-7.21	7E-11	6E-10	7.4	5.35	-2.05
7041	TGFB1I1	5.23	0.0000008	0.000003	6.45	7.99	1.53
51621	KLF13	3.93	0.0001	0.0003	9.3	10.13	0.83
51111	SUV420H1	-3.1	0.002	0.004	8.1	7.63	-0.47
10365	KLF2	5.46	0.0000003	0.000001	7.4	11.29	3.89
51450	PRRX2	-3.3	0.001	0.002	8.98	7.7	-1.28
54345	SOX18	7.86	3E-12	3E-11	6.99	12.03	5.03
9496	TBX4	29.28	9E-54	2E-51	5.19	9.12	3.94
55888	ZNF167	3.25	0.002	0.003	6.79	7.6	0.81
3202	HOXA5	9.98	4E-17	7E-16	6.44	13.65	7.21
57336	ZNF287	3.38	0.001	0.002	5.88	6.88	1
60468	BACH2	2.35	0.021	0.024	6.44	7.17	0.72
8864	PER2	4.07	0.00009	0.0002	8.82	9.93	1.11
3203	HOXA6	7.54	1E-11	1E-10	6.24	8.09	1.85
3241	HPCAL1	4.24	0.00005	0.0001	7.44	8.56	1.12
80333	KCNIP4	7.61	1E-11	9E-11	5.49	6.23	0.74
1831	TSC22D3	5.01	0.000002	0.000008	6.46	7.65	1.19
6943	TCF21	21.96	5E-42	5E-40	5.61	11.71	6.1

Table S2: This table lists all the TFs from Table S1, which are significantly differentially expressed between 107 hESC samples and 2 fetal lung samples from the SCM2 expression compendium. We give the Entrez gene ID, the symbol, the regularised t-statistic, its P-value, its adjusted P-value, the average expression level in hESC and tissue and the log fold change.

**Entrez GID**	**Symbol**	**t**	**P**	**P-adj**	**AvExpESC**	**AvExpTIS**	**logFC**
430	ASCL2	9.22	2E-15	3E-14	6.54	9.16	2.62
463	ZFHX3	5.84	0.00000005	0.0000003	6.82	9.99	3.17
467	ATF3	-5.14	0.000001	0.000005	7.57	5.9	-1.67
54897	CASZ1	2.95	0.004	0.006	6.69	7.37	0.68
57332	CBX8	4.56	0.00001	0.00004	5.97	6.64	0.67
4435	CITED1	7.61	1E-11	9E-11	5.77	8.1	2.33
1602	DACH1	8.15	6E-13	7E-12	6.2	9.05	2.85
8110	DPF3	3.53	0.0006	0.001	5.71	6.25	0.54
1960	EGR3	-2.88	0.005	0.007	6.34	5.7	-0.64
2034	EPAS1	3.32	0.001	0.002	7.64	10.71	3.07
2313	FLI1	3.21	0.002	0.003	5.3	5.87	0.57
2306	FOXD2	32.54	3E-58	9E-56	5.41	10.88	5.47
27022	FOXD3	-4.38	0.00003	0.00008	6.73	5.37	-1.36
2302	FOXJ1	2.76	0.007	0.009	5.69	6.37	0.68
2624	GATA2	7.16	1E-10	8E-10	5.54	7.56	2.02
2625	GATA3	7.89	2E-12	2E-11	5.83	9.83	4
23462	HEY1	4.42	0.00002	0.00007	6.98	9.02	2.05
3142	HLX	3.94	0.0001	0.0003	5.95	7.01	1.06
3211	HOXB1	-2.74	0.007	0.01	6.75	6.13	-0.63
3212	HOXB2	4.53	0.00001	0.00004	6.89	11.22	4.33
3213	HOXB3	14.1	2E-26	6E-25	5.37	8.37	3
3216	HOXB6	4.43	0.00002	0.00006	6.71	7.82	1.11
3217	HOXB7	9.64	3E-16	4E-15	5.87	11.19	5.32
3218	HOXB8	6.43	0.000000003	0.00000002	6.17	11.33	5.16
3221	HOXC4	17.86	3E-34	2E-32	5.65	10.01	4.36
3222	HOXC5	8.27	3E-13	4E-12	5.36	6.28	0.92
3223	HOXC6	5.67	0.0000001	0.0000006	6.33	8.41	2.09

3224	HOXC8	3.42	0.0009	0.002	6.31	9.05	2.74
3231	HOXD1	26.67	8E-50	1E-47	5.5	10.74	5.23
3232	HOXD3	12.13	5E-22	1E-20	6.63	9.03	2.39
3233	HOXD4	14.75	8E-28	3E-26	5.62	7.85	2.23
3235	HOXD9	21.68	2E-41	1E-39	5.24	7.49	2.25
3299	HSF4	-2.28	0.025	0.026	5.35	5.15	-0.2
10265	IRX5	4.1	0.00008	0.0002	6.04	7.67	1.64
9935	MAFB	8.63	5E-14	6E-13	7.34	12.26	4.92
4784	NFIX	3.58	0.0005	0.001	6.95	10.38	3.43
4821	NKX2-2	-2.43	0.017	0.02	5.49	5.18	-0.3
4824	NKX3-1	3.5	0.0007	0.001	6.04	7.06	1.02
84504	NKX6-2	3.23	0.002	0.003	5.64	6.27	0.63
4861	NPAS1	-6.07	0.00000002	0.0000001	8.87	6.62	-2.25
7026	NR2F2	9.03	7E-15	8E-14	6.3	10.21	3.91
9480	ONECUT2	-2.55	0.012	0.015	5.8	5.47	-0.34
130497	OSR1	7.41	3E-11	2E-10	5.89	9.92	4.04
5076	PAX2	24.42	3E-46	4E-44	5.39	8.24	2.85
5081	PAX7	-3.17	0.002	0.003	8.3	7.59	-0.72
7849	PAX8	30.92	4E-56	1E-53	5.47	8.61	3.13
8929	PHOX2B	6.29	0.000000007	0.00000004	5.65	7	1.34
63876	PKNOX2	3.13	0.002	0.004	6.52	7.85	1.34
28984	C13orf15	4.32	0.00003	0.00009	7.27	10.32	3.05
6493	SIM2	10.88	4E-19	7E-18	5.42	6.83	1.41
6495	SIX1	2.62	0.01	0.013	5.33	5.65	0.32
10736	SIX2	16	2E-30	7E-29	5.28	7.11	1.82
55509	BATF3	-2.28	0.024	0.026	7.05	6.15	-0.9
6909	TBX2	7.36	3E-11	3E-10	5.73	9.96	4.22
6926	TBX3	4.5	0.00002	0.00005	6.22	7.6	1.39
6928	HNF1B	12.23	3E-22	7E-21	5.55	8.47	2.92
7080	NKX2-1	-2.87	0.005	0.007	5.24	5.04	-0.2
7490	WT1	45.77	2E-73	2E-70	5.66	10.29	4.62
9839	ZEB2	6.04	0.00000002	0.0000001	6.36	9.53	3.17
63976	PRDM16	12.38	1E-22	3E-21	5.35	7.91	2.56
633	BGN	2.7	0.008	0.011	10.44	13.98	3.55
26038	CHD5	-2.32	0.022	0.024	6.86	5.62	-1.24
5452	POU2F2	-2.69	0.008	0.011	5.4	4.92	-0.48
10475	TRIM38	4.23	0.00005	0.0001	5.98	7.21	1.23
7593	MZF1	4.6	0.00001	0.00004	7.64	8.34	0.69
2103	ESRRB	2.02	0.045	0.043	5.76	6.06	0.29
154796	AMOT	4.79	0.000005	0.00002	7.99	10.36	2.37
3200	HOXA3	25.81	2E-48	2E-46	5.29	8.17	2.89
3206	HOXA10	25.79	2E-48	2E-46	5.39	8.82	3.43
64067	NPAS3	9.12	4E-15	5E-14	5.45	6.46	1.01
2334	AFF2	3.22	0.002	0.003	6.32	7.25	0.93
4793	NFKBIB	-5.06	0.000002	0.000006	6.78	5.95	-0.83
10150	MBNL2	6.55	0.000000002	0.00000001	6.06	8.36	2.3
23414	ZFPM2	7.15	1E-10	8E-10	5.5	7.33	1.83
10608	MXD4	7.57	1E-11	1E-10	10.15	12.08	1.92
51176	LEF1	3.92	0.0002	0.0004	7.14	10.47	3.33
9330	GTF3C3	-3.07	0.003	0.004	10.18	9.5	-0.69
4306	NR3C2	7.48	2E-11	2E-10	6.38	7.71	1.33
55663	ZNF446	3.84	0.0002	0.0005	7.35	8.61	1.26
83463	MXD3	4.74	0.000006	0.00002	7.19	8.36	1.18
116039	OSR2	9.99	4E-17	6E-16	5.39	6.44	1.05

6935	ZEB1	3.39	0.001	0.002	5.59	6.55	0.96
1396	CRIP1	3.92	0.0002	0.0004	7.53	9.68	2.15
8216	LZTR1	4.37	0.00003	0.00008	9.97	10.99	1.03
10848	PPP1R13L	-4.13	0.00007	0.0002	7.4	5.99	-1.41
54760	PCSK4	-2	0.048	0.045	6.31	5.87	-0.44
3663	IRF5	4.97	0.000002	0.000009	5.41	5.85	0.44
56270	WDR45L	-2.88	0.005	0.007	9.95	9.17	-0.79
51042	ZNF593	-3.68	0.0004	0.0008	10.03	9.22	-0.81
4066	LYL1	7.46	2E-11	2E-10	6.89	9.58	2.7
6015	RING1	4.94	0.000003	0.00001	10.87	12.03	1.15
80758	PRR7	-4.56	0.00001	0.00004	7.08	5.91	-1.16
29915	HCFC2	5.12	0.000001	0.000005	5.89	6.89	1
5468	PPARG	13.18	2E-24	6E-23	5.5	7.56	2.06
23493	HEY2	-4.32	0.00003	0.00009	10.53	7.9	-2.64
3659	IRF1	3.32	0.001	0.002	7.73	8.8	1.07
8535	CBX4	2.36	0.02	0.023	6.37	7.14	0.77
2118	ETV4	-2.21	0.029	0.03	8.64	7.64	-1
7020	TFAP2A	2.27	0.025	0.027	6.6	7.64	1.04
6322	SCML1	3.52	0.0006	0.001	6.46	7.39	0.92
7067	THRA	5.41	0.0000004	0.000002	6.3	7.8	1.5
1959	EGR2	-2.09	0.039	0.038	7.6	6.13	-1.47
7021	TFAP2B	5.91	0.00000004	0.0000002	5.41	6.47	1.06
1879	EBF1	13.97	4E-26	1E-24	5.81	9.53	3.72
3717	JAK2	2.4	0.018	0.021	6.39	6.97	0.57
2070	EYA4	14.05	3E-26	8E-25	5.36	6.44	1.07
30820	KCNIP1	10.57	2E-18	3E-17	5.6	7.96	2.36
1870	E2F2	2.12	0.036	0.036	8.85	10.28	1.43
26040	SETBP1	5.18	0.000001	0.000004	7.78	10.37	2.59
1050	CEBPA	2.47	0.015	0.018	6.85	8.29	1.44
1523	CUX1	3.19	0.002	0.003	6.77	7.26	0.49
6595	SMARCA2	5.39	0.0000004	0.000002	8.99	10.96	1.97
2309	FOXO3	3.15	0.002	0.003	10.35	11.29	0.94
56978	PRDM8	3.14	0.002	0.003	6.01	7.01	1
5914	RARA	2.95	0.004	0.006	7.51	8.01	0.5
4005	LMO2	6.36	0.000000005	0.00000003	6.66	8.58	1.92
23119	HIC2	-2.15	0.034	0.034	10.78	10.12	-0.66
2122	EVI1	18.07	1E-34	6E-33	5.97	12.04	6.08
1397	CRIP2	6.69	0.000000001	0.000000007	7.09	10.99	3.9
4790	NFKB1	6.04	0.00000002	0.0000001	9.27	11.71	2.43
2908	NR3C1	3.12	0.002	0.004	5.89	6.32	0.44
6662	SOX9	3.25	0.002	0.003	7.34	9.11	1.77
5990	RFX2	2.3	0.023	0.025	5.52	5.98	0.47
7580	ZNF32	3.53	0.0006	0.001	7.92	8.55	0.63
1628	DBP	-2.16	0.033	0.033	6.57	5.82	-0.76
2296	FOXC1	6.46	0.000000003	0.00000002	7.05	13.39	6.34
3131	HLF	2.64	0.009	0.012	5.46	5.82	0.36
3201	HOXA4	26.75	6E-50	9E-48	5.25	8.07	2.82
3215	HOXB5	4.87	0.000004	0.00001	7.04	12.29	5.25
3236	HOXD10	33.76	7E-60	3E-57	5.32	9.49	4.18
3899	AFF3	6.79	6E-10	0.000000004	6.23	9.67	3.44
4097	MAFG	-4.16	0.00006	0.0002	8	6.82	-1.18
4208	MEF2C	11.31	4E-20	8E-19	5.41	7.21	1.81
4211	MEIS1	8.05	1E-12	1E-11	6.31	10.73	4.42
4212	MEIS2	7.18	9E-11	7E-10	6.26	9.18	2.93

4862	NPAS2	3.97	0.0001	0.0003	5.89	6.53	0.64
5629	PROX1	5.93	0.00000004	0.0000002	5.53	6.7	1.17
6839	SUV39H1	-2.25	0.027	0.028	10.22	9.57	-0.65
7022	TFAP2C	-9.23	2E-15	3E-14	9.82	6	-3.82
8433	UTF1	-3.8	0.0002	0.0005	7.38	5.09	-2.28
8507	ENC1	3.84	0.0002	0.0005	8.41	10.66	2.26
8804	CREG1	6.75	7E-10	0.000000005	9.17	10.51	1.33
8928	FOXH1	-2.48	0.015	0.018	6.94	6.41	-0.53
2016	EMX1	13.55	3E-25	9E-24	5.48	7.92	2.45
2018	EMX2	16.86	3E-32	1E-30	5.81	10.26	4.45
754	PTTG1IP	4.16	0.00006	0.0002	11.37	12.17	0.8
3169	FOXA1	3.53	0.0006	0.001	6.26	8.95	2.69
5017	OVOL1	3.73	0.0003	0.0006	5.52	6.23	0.72
6492	SIM1	5.29	0.0000006	0.000003	5.57	6.58	1.01
648	BMI1	7.08	1E-10	0.000000001	8.6	11.16	2.56
2303	FOXC2	5.09	0.000001	0.000006	6.35	7.98	1.63
3149	HMGB3	-6.54	0.000000002	0.00000001	11.33	9.58	-1.75
3207	HOXA11	14.52	3E-27	8E-26	5.31	7.56	2.26
3280	HES1	2.92	0.004	0.006	7.32	8.91	1.59
3975	LHX1	6.79	6E-10	0.000000004	6.22	9.15	2.93
4782	NFIC	3.18	0.002	0.003	5.62	6.11	0.49
6658	SOX3	-7.44	2E-11	2E-10	8.94	5.25	-3.69
7025	NR2F1	10.26	1E-17	2E-16	5.81	11.41	5.6
5813	PURA	3.15	0.002	0.003	6.42	7.21	0.79
4209	MEF2D	2.83	0.006	0.008	8.4	9.09	0.7
4223	MEOX2	4.07	0.00009	0.0002	5.23	5.67	0.44
4772	NFATC1	-2.03	0.044	0.042	6.77	6.03	-0.74
7746	ZNF193	3.01	0.003	0.005	8.62	9.54	0.91
3090	HIC1	4.69	0.000008	0.00003	5.97	6.89	0.92
3199	HOXA2	8.43	1E-13	2E-12	5.72	8.71	2.99
6663	SOX10	-2.53	0.013	0.016	6.36	5.57	-0.79
7391	USF1	-2.18	0.031	0.032	7.79	7.14	-0.64
6772	STAT1	2.87	0.005	0.007	9.29	9.94	0.65
26574	AATF	-2.19	0.03	0.031	9.86	9.25	-0.61
27287	VENTX	-6.57	0.000000002	0.00000001	8.44	5.76	-2.68
29842	TFCP2L1	7.27	6E-11	5E-10	6.69	9.4	2.71
8022	LHX3	-2.1	0.038	0.037	5.48	5.2	-0.28
26508	HEYL	6.51	0.000000002	0.00000001	7.37	10.66	3.3
9792	SERTAD2	4.62	0.00001	0.00003	9.92	11.43	1.51
22882	ZHX2	1.99	0.049	0.046	8.09	8.83	0.74
23237	ARC	-2.48	0.015	0.018	6.25	5.34	-0.91
23314	SATB2	2.44	0.016	0.019	7.63	8.72	1.09
23316	CUX2	2.36	0.02	0.022	7.4	8.07	0.67
7041	TGFB1I1	3.73	0.0003	0.0007	6.45	7.55	1.09
51621	KLF13	2.76	0.007	0.009	9.3	9.89	0.58
51111	SUV420H1	-4.45	0.00002	0.00006	8.1	7.42	-0.68
10365	KLF2	5.22	0.0000009	0.000003	7.4	11.12	3.72
51450	PRRX2	-3.88	0.0002	0.0004	8.98	7.48	-1.5
3226	HOXC10	12.38	1E-22	3E-21	5.44	6.99	1.55
54345	SOX18	6.85	4E-10	0.000000003	6.99	11.38	4.38
55888	ZNF167	2.64	0.009	0.012	6.79	7.45	0.66
3202	HOXA5	8.88	1E-14	2E-13	6.44	12.85	6.42
57336	ZNF287	2.97	0.004	0.005	5.88	6.76	0.88
3237	HOXD11	17.95	2E-34	1E-32	5.79	9.82	4.03

58495	OVOL2	-7.47	2E-11	2E-10	10.16	7.27	-2.89
60468	BACH2	2.15	0.033	0.034	6.44	7.11	0.67
8864	PER2	3.19	0.002	0.003	8.82	9.69	0.87
3203	HOXA6	11.88	2E-21	4E-20	6.24	9.22	2.98
3219	HOXB9	2.54	0.013	0.015	5.3	5.53	0.23
94234	FOXQ1	3.62	0.0004	0.0009	7.19	10.76	3.58
80333	KCNIP4	14.06	3E-26	8E-25	5.49	6.85	1.36
3205	HOXA9	17.45	2E-33	9E-32	5.47	9.17	3.69
1831	TSC22D3	4.84	0.000004	0.00001	6.46	7.61	1.15
6943	TCF21	21.47	4E-41	3E-39	5.61	11.58	5.97
2104	ESRRG	7.58	1E-11	1E-10	6.26	8.07	1.82

Table S3: This table lists all the TFs from Table S1, which are significantly differentially expressed between 107 hESC samples and 2 fetal kidney samples from the SCM2 expression compendium. We give the Entrez gene ID, the symbol, the regularised t-statistic, its P-value, its adjusted P-value, the average expression level in hESC and tissue and the log fold change.

Entrez GID	Symbol	t	P	P-adj	AvExpESC	AvExpTIS	logFC
430	ASCL2	10.61	2E-18	1E-17	6.54	9.56	3.02
463	ZFHX3	7.34	4E-11	2E-10	6.82	10.8	3.98
467	ATF3	2.55	0.012	0.012	7.57	8.39	0.83
79365	BHLHB3	19.52	2E-37	6E-36	5.64	9	3.37
54897	CASZ1	9.59	3E-16	3E-15	6.69	8.91	2.22
1602	DACH1	3.54	0.0006	0.0008	6.2	7.44	1.24
2034	EPAS1	5.11	0.000001	0.000003	7.64	12.36	4.72
2306	FOXD2	14.02	3E-26	5E-25	5.41	7.77	2.36
27022	FOXD3	-5.26	0.0000007	0.000002	6.73	5.1	-1.63
2294	FOXF1	10.2	1E-17	1E-16	5.76	8.99	3.22
2624	GATA2	12.55	6E-23	8E-22	5.54	9.23	3.69
2625	GATA3	9.12	4E-15	3E-14	5.83	10.47	4.64
9464	HAND2	6.55	0.000000002	0.000000007	5.75	7.26	1.51
3142	HLX	2.12	0.036	0.031	5.95	6.52	0.57
3211	HOXB1	-3.34	0.001	0.002	6.75	5.97	-0.78
3213	HOXB3	5.66	0.0000001	0.0000004	5.37	6.59	1.22
3217	HOXB7	7.94	2E-12	1E-11	5.87	10.26	4.39
3231	HOXD1	22.33	1E-42	6E-41	5.5	9.88	4.37
3238	HOXD12	-3.05	0.003	0.003	7.49	6.75	-0.74
3299	HSF4	3.9	0.0002	0.0003	5.35	5.69	0.34
50805	IRX4	-2.11	0.037	0.032	6.4	5.52	-0.89
3670	ISL1	2.18	0.031	0.028	7.66	9.59	1.93
9314	KLF4	5.14	0.000001	0.000003	6.56	7.96	1.41
26468	LHX6	-3.15	0.002	0.003	7.25	6.44	-0.81
9935	MAFB	3.24	0.002	0.002	7.34	9.19	1.85
4784	NFIX	4.56	0.00001	0.00003	6.95	11.32	4.37
4821	NKX2-2	-2.08	0.04	0.034	5.49	5.23	-0.26
4824	NKX3-1	3.36	0.001	0.001	6.04	7.03	0.99
84504	NKX6-2	-2.55	0.012	0.012	5.64	5.14	-0.5
4861	NPAS1	-6.33	0.000000006	0.00000002	8.87	6.53	-2.34
7026	NR2F2	3.78	0.0003	0.0004	6.3	7.93	1.63
9480	ONECUT2	-3.06	0.003	0.003	5.8	5.4	-0.41
130497	OSR1	10.62	1E-18	1E-17	5.89	11.67	5.78

5241	PGR	2.95	0.004	0.005	5.29	5.69	0.41
5307	PITX1	3.79	0.0002	0.0004	7.23	12.32	5.09
28984	C13orf15	5.72	0.00000009	0.0000003	7.27	11.33	4.06
10736	SIX2	3.34	0.001	0.002	5.28	5.66	0.38
6909	TBX2	6.3	0.000000006	0.00000002	5.73	9.34	3.61
6926	TBX3	6.41	0.000000004	0.00000001	6.22	8.19	1.98
6928	HNF1B	5.92	0.00000004	0.0000001	5.55	6.97	1.42
3196	TLX2	3.31	0.001	0.002	5.25	5.68	0.43
7704	ZBTB16	16.98	2E-32	4E-31	5.46	9.44	3.98
9839	ZEB2	3.69	0.0004	0.0005	6.36	8.3	1.93
22806	IKZF3	2.02	0.046	0.038	5.65	5.85	0.2
8863	PER3	2.98	0.004	0.004	7.55	8.61	1.06
63976	PRDM16	2.09	0.039	0.033	5.35	5.78	0.43
26038	CHD5	-2.66	0.009	0.009	6.86	5.44	-1.43
10475	TRIM38	7.79	4E-12	2E-11	5.98	8.24	2.26
7593	MZF1	3.3	0.001	0.002	7.64	8.14	0.5
2000	ELF4	8.26	4E-13	2E-12	7.81	10.08	2.27
4661	MYT1	-2.87	0.005	0.006	6.49	5.21	-1.28
6939	TCF15	-2.14	0.035	0.03	6.64	6	-0.64
602	BCL3	4.31	0.00004	0.00007	6.98	9.21	2.23
11043	MID2	3.86	0.0002	0.0003	6.52	7.74	1.21
89870	TRIM15	-2.01	0.047	0.039	5.51	5.27	-0.24
3200	HOXA3	10.48	3E-18	3E-17	5.29	6.61	1.33
3206	HOXA10	15.31	5E-29	1E-27	5.39	7.44	2.05
2334	AFF2	-2.91	0.004	0.005	6.32	5.47	-0.85
4793	NFKBIB	-3.33	0.001	0.002	6.78	6.23	-0.55
7539	ZFP37	-2.46	0.016	0.015	8.2	7.32	-0.88
406	ARNTL	2.64	0.01	0.01	6.72	7.27	0.55
2100	ESR2	4.06	0.00009	0.0002	5.35	5.67	0.32
10150	MBNL2	7.79	4E-12	2E-11	6.06	8.79	2.73
23414	ZFPM2	5.67	0.0000001	0.0000003	5.5	6.95	1.45
10608	MXD4	5.96	0.00000003	0.0000001	10.15	11.67	1.51
2099	ESR1	21.74	1E-41	6E-40	5.24	7.8	2.56
9330	GTF3C3	-4.63	0.00001	0.00002	10.18	9.15	-1.04
9112	MTA1	-2.53	0.013	0.013	10.21	9.65	-0.56
4306	NR3C2	9.16	3E-15	2E-14	6.38	8.03	1.65
79890	RIN3	4.39	0.00003	0.00005	5.33	5.99	0.66
55734	ZFP64	-2.19	0.031	0.027	6.92	6.22	-0.69
3229	HOXC13	3.27	0.001	0.002	5.55	6.12	0.57
55663	ZNF446	2.86	0.005	0.006	7.35	8.3	0.95
83463	MXD3	-3.15	0.002	0.003	7.19	6.41	-0.78
116039	OSR2	13.19	2E-24	3E-23	5.39	6.8	1.41
6935	ZEB1	4.97	0.000002	0.000006	5.59	7	1.41
1396	CRIP1	4.39	0.00003	0.00005	7.53	9.94	2.41
7942	TFEB	5.85	0.00000005	0.0000002	5.36	6.12	0.75
27086	FOXP1	3.22	0.002	0.002	6	6.55	0.55
8216	LZTR1	2.37	0.019	0.018	9.97	10.53	0.56
10848	PPP1R13L	4.2	0.00005	0.0001	7.4	8.84	1.44
54796	BNC2	-2.74	0.007	0.008	9.45	8.3	-1.15
79776	ZFHX4	3.76	0.0003	0.0004	6.03	7.75	1.71
3164	NR4A1	4.24	0.00005	0.00008	5.4	6.14	0.74
1674	DES	31.65	4E-57	6E-55	5.49	11.12	5.62
3663	IRF5	6.09	0.00000002	0.00000005	5.41	5.96	0.54
1820	ARID3A	-9.04	6E-15	4E-14	11.23	7.91	-3.32

4066	LYL1	5.07	0.000002	0.000004	6.89	8.72	1.83
6015	RING1	3.87	0.0002	0.0003	10.87	11.78	0.91
80758	PRR7	-4.71	0.000007	0.00002	7.08	5.87	-1.21
29915	HCFC2	6.56	0.000000002	0.000000007	5.89	7.17	1.28
5468	PPARG	22.62	3E-43	2E-41	5.5	9.05	3.55
23493	HEY2	-7.1	1E-10	6E-10	10.53	6.2	-4.33
3659	IRF1	6.78	6E-10	0.000000002	7.73	9.97	2.24
51085	MLXIPL	-2.52	0.013	0.013	6.48	5.69	-0.8
2139	EYA2	4.19	0.00006	0.0001	6.95	9.81	2.86
7799	PRDM2	2.54	0.013	0.013	5.76	6.08	0.32
2118	ETV4	-6.95	3E-10	0.000000001	8.64	5.49	-3.15
2972	BRF1	2.28	0.025	0.023	6.46	7.11	0.65
2113	ETS1	1.99	0.049	0.04	10.47	11.54	1.08
6322	SCML1	4.94	0.000003	0.000007	6.46	7.76	1.29
7067	THRA	4.15	0.00007	0.0001	6.3	7.46	1.16
28999	KLF15	2	0.048	0.04	7.05	7.86	0.81
1879	EBF1	12.41	1E-22	2E-21	5.81	9.13	3.32
3717	JAK2	7.62	1E-11	5E-11	6.39	8.22	1.83
2070	EYA4	11.23	6E-20	6E-19	5.36	6.21	0.85
1870	E2F2	-4.48	0.00002	0.00004	8.85	5.83	-3.02
26040	SETBP1	3.78	0.0003	0.0004	7.78	9.68	1.9
1050	CEBPA	5.75	0.00000008	0.0000002	6.85	10.21	3.36
6595	SMARCA2	5.53	0.0000002	0.0000006	8.99	11.01	2.02
2309	FOXO3	4.45	0.00002	0.00004	10.35	11.67	1.32
56978	PRDM8	8.83	2E-14	1E-13	6.01	8.81	2.8
5914	RARA	3.57	0.0005	0.0008	7.51	8.12	0.6
4005	LMO2	4.36	0.00003	0.00005	6.66	7.96	1.3
23119	HIC2	-7.61	1E-11	5E-11	10.78	8.44	-2.34
2122	EVI1	18.38	2E-35	8E-34	5.97	12.16	6.19
1397	CRIP2	6.77	7E-10	0.000000003	7.09	11.04	3.95
1326	MAP3K8	5.33	0.0000005	0.000001	6.99	9.78	2.79
861	RUNX1	6.79	6E-10	0.000000002	5.55	7.35	1.8
4790	NFKB1	8.2	5E-13	3E-12	9.27	12.57	3.3
2908	NR3C1	3.81	0.0002	0.0004	5.89	6.42	0.53
5990	RFX2	11.24	6E-20	6E-19	5.52	7.98	2.46
7580	ZNF32	2.49	0.014	0.014	7.92	8.36	0.44
687	KLF9	6.06	0.00000002	0.00000006	8.18	12.8	4.62
2273	FHL1	6.49	0.000000003	0.000000009	8.01	10.96	2.95
2274	FHL2	5.07	0.000002	0.000004	9.05	10.01	0.96
2295	FOXF2	8.61	6E-14	4E-13	5.63	7.49	1.86
2296	FOXC1	4.82	0.000005	0.00001	7.05	11.77	4.72
688	KLF5	6.57	0.000000002	0.000000007	5.81	8.64	2.83
1958	EGR1	2.36	0.02	0.019	11.13	12.4	1.27
3131	HLF	15.87	3E-30	7E-29	5.46	7.61	2.15
3201	HOXA4	12.44	1E-22	1E-21	5.25	6.57	1.32
3215	HOXB5	2.03	0.045	0.038	7.04	9.23	2.19
3642	INSM1	-2.81	0.006	0.006	7.08	5.7	-1.37
3726	JUNB	8.76	3E-14	2E-13	6.74	10.72	3.98
3899	AFF3	5.12	0.000001	0.000003	6.23	8.83	2.6
4208	MEF2C	9.09	5E-15	3E-14	5.41	6.88	1.47
4211	MEIS1	4.96	0.000003	0.000006	6.31	9.03	2.72
4212	MEIS2	4.17	0.00006	0.0001	6.26	7.96	1.7
4488	MSX2	4.67	0.000009	0.00002	5.77	7.01	1.23
4862	NPAS2	16.69	6E-32	2E-30	5.89	8.57	2.68

5629	PROX1	8.13	7E-13	4E-12	5.53	7.12	1.58
6776	STAT5A	5.35	0.0000005	0.000001	7.19	9.34	2.15
6839	SUV39H1	-4.16	0.00006	0.0001	10.22	9.01	-1.21
7022	TFAP2C	-2.13	0.035	0.03	9.82	8.94	-0.88
8433	UTF1	-3.55	0.0006	0.0008	7.38	5.24	-2.13
8507	ENC1	3.5	0.0007	0.0009	8.41	10.46	2.05
8553	BHLHB2	3.69	0.0004	0.0005	9.21	12.14	2.93
8804	CREG1	11.36	3E-20	3E-19	9.17	11.42	2.25
8928	FOXH1	-4.32	0.00003	0.00006	6.94	6.01	-0.93
1465	CSRP1	3.49	0.0007	0.001	8.63	12.99	4.36
2018	EMX2	3.3	0.001	0.002	5.81	6.69	0.87
754	PTTG1IP	3.17	0.002	0.003	11.37	11.98	0.61
860	RUNX2	4.09	0.00008	0.0001	5.52	6.29	0.77
864	RUNX3	3.81	0.0002	0.0004	6.95	8.73	1.78
2119	ETV5	-2.07	0.041	0.035	8.19	7.08	-1.11
3169	FOXA1	8.54	8E-14	5E-13	6.26	12.76	6.5
5017	OVOL1	12.63	4E-23	5E-22	5.52	7.93	2.42
648	BMI1	6.65	0.000000001	0.000000004	8.6	11.01	2.41
2353	FOS	2.43	0.017	0.016	10.11	12.54	2.43
2355	FOSL2	3.25	0.002	0.002	5.76	6.49	0.73
2672	GFI1	3.76	0.0003	0.0004	5.35	5.82	0.47
3149	HMGB3	-12.19	4E-22	5E-21	11.33	8.07	-3.27
4783	NFIL3	2.17	0.032	0.029	7.82	8.93	1.11
3207	HOXA11	9.12	4E-15	3E-14	5.31	6.79	1.48
3280	HES1	2.28	0.025	0.023	7.32	8.56	1.24
4782	NFIC	4.72	0.000007	0.00002	5.62	6.34	0.72
6658	SOX3	-7.26	6E-11	3E-10	8.94	5.34	-3.6
7025	NR2F1	5.93	0.00000004	0.0000001	5.81	9.05	3.24
5813	PURA	5.34	0.0000005	0.000001	6.42	7.8	1.38
4209	MEF2D	6.03	0.00000002	0.00000007	8.4	9.88	1.48
4929	NR4A2	4.52	0.00002	0.00003	5.96	8.57	2.61
3090	HIC1	3.32	0.001	0.002	5.97	6.61	0.64
2354	FOSB	3.09	0.003	0.003	9.57	13.59	4.03
3199	HOXA2	3.02	0.003	0.004	5.72	6.8	1.07
6096	RORB	4.94	0.000003	0.000006	5.94	7.51	1.57
6663	SOX10	-2.9	0.004	0.005	6.36	5.45	-0.91
23764	MAFF	2.38	0.019	0.018	6.8	7.57	0.77
23413	FREQ	2.74	0.007	0.008	8.9	9.67	0.77
27287	VENTX	-5.53	0.0000002	0.0000006	8.44	6.18	-2.26
7775	ZNF232	-4.46	0.00002	0.00004	8.66	7.36	-1.29
29842	TFCP2L1	5.57	0.0000002	0.0000005	6.69	8.77	2.08
9792	SERTAD2	5.57	0.0000002	0.0000005	9.92	11.73	1.81
23237	ARC	-2.25	0.027	0.024	6.25	5.43	-0.82
23316	CUX2	-7.05	2E-10	7E-10	7.4	5.39	-2.01
51341	ZBTB7A	2.96	0.004	0.004	5.63	6.18	0.56
7041	TGFB1I1	4.62	0.00001	0.00002	6.45	7.81	1.35
51621	KLF13	6.16	0.00000001	0.00000004	9.3	10.6	1.3
51111	SUV420H1	-2.63	0.01	0.01	8.1	7.7	-0.4
10365	KLF2	6.03	0.00000002	0.00000007	7.4	11.7	4.3
54345	SOX18	3.85	0.0002	0.0003	6.99	9.47	2.47
55888	ZNF167	-3.82	0.0002	0.0004	6.79	5.83	-0.95
3202	HOXA5	7.83	3E-12	2E-11	6.44	12.1	5.66
57336	ZNF287	2.13	0.035	0.031	5.88	6.55	0.68
3237	HOXD11	2.53	0.013	0.013	5.79	6.38	0.58

60468	BACH2	-2.75	0.007	0.007	6.44	5.59	-0.85
8864	PER2	6.14	0.00000001	0.00000004	8.82	10.55	1.73
3203	HOXA6	6.83	5E-10	0.000000002	6.24	8.07	1.83
79943	ZNF696	-3.51	0.0006	0.0009	8.48	7.54	-0.93
94234	FOXQ1	5.61	0.0000002	0.0000004	7.19	12.72	5.53
3205	HOXA9	13.11	3E-24	5E-23	5.47	8.27	2.79
23492	CBX7	8.6	6E-14	4E-13	7.83	10.83	2.99
7275	TUB	-4.31	0.00004	0.00007	8.24	7.07	-1.17
1831	TSC22D3	9.83	1E-16	8E-16	6.46	8.8	2.34
6943	TCF21	13.09	4E-24	5E-23	5.61	9.25	3.64
571	BACH1	-2.1	0.038	0.033	6.06	5.66	-0.41

Table S4: This table lists all the TFs from Table S1, which are significantly differentially expressed between 107 hESC samples and 2 adult bladder samples from the SCM2 expression compendium. We give the Entrez gene ID, the symbol, the regularised t-statistic, its P-value, its adjusted P-value, the average expression level in hESC and tissue and the log fold change.

Entrez GID	Symbol	t	P	P-adj	AvExpESC	AvExpTIS	logFC
429	ASCL1	3.89	2.00E-04	3.00E-04	5.95	7.85	1.9
430	ASCL2	12.6	4.00E-23	8.00E-22	6.54	10.13	3.59
463	ZFHX3	4.79	5.00E-06	1.00E-05	6.82	9.42	2.6
474	ATOH1	5.18	1.00E-06	3.00E-06	5.39	6.07	0.68
579	NKX3-2	4.91	3.00E-06	8.00E-06	5.85	9.48	3.63
8538	BARX2	3.68	4.00E-04	6.00E-04	5.61	6.3	0.69
79365	BHLHB3	3.88	2.00E-04	3.00E-04	5.64	6.37	0.73
54897	CASZ1	6.82	5.00E-10	2.00E-09	6.69	8.43	1.74
1045	CDX2	8.93	1.00E-14	9.00E-14	5.85	10.55	4.69
1602	DACH1	4.24	5.00E-05	9.00E-05	6.2	7.7	1.5
2034	EPAS1	3.78	3.00E-04	4.00E-04	7.64	11.15	3.51
54738	FEV	11.23	6.00E-20	8.00E-19	5.39	6.89	1.5
2306	FOXD2	9.44	8.00E-16	7.00E-15	5.41	7	1.59
27022	FOXD3	-4.35	3.00E-05	6.00E-05	6.73	5.38	-1.35
2294	FOXF1	8.47	1.00E-13	8.00E-13	5.76	8.48	2.71
2626	GATA4	2.71	0.008	0.008	5.64	6.88	1.24
2627	GATA6	2.04	0.044	0.036	5.62	6.06	0.44
9464	HAND2	10.04	3.00E-17	3.00E-16	5.75	8.06	2.31
3142	HLX	7.22	7.00E-11	4.00E-10	5.95	7.9	1.95
3211	HOXB1	-2.13	0.035	0.03	6.75	6.27	-0.49
3212	HOXB2	2.45	0.016	0.016	6.89	9.23	2.34
3213	HOXB3	5.15	1.00E-06	3.00E-06	5.37	6.56	1.19
3217	HOXB7	3.63	4.00E-04	7.00E-04	5.87	7.94	2.07
3221	HOXC4	6.98	2.00E-10	1.00E-09	5.65	7.55	1.9
3231	HOXD1	10.78	6.00E-19	8.00E-18	5.5	7.86	2.36
3238	HOXD12	-2.22	0.029	0.025	7.49	6.96	-0.53

3232	HOXD3	1.99	0.049	0.04	6.63	7.03	0.39
3233	HOXD4	2.45	0.016	0.016	5.62	6.01	0.39
3299	HSF4	4.15	7.00E-05	1.00E-04	5.35	5.75	0.4
50805	IRX4	-2.5	0.014	0.014	6.4	5.35	-1.05
3725	JUN	2.87	0.005	0.006	10.51	12.56	2.04
9314	KLF4	3.97	1.00E-04	2.00E-04	6.56	7.59	1.03
9935	MAFB	3.14	0.002	0.003	7.34	9.13	1.79
4760	NEUROD1	4.11	8.00E-05	1.00E-04	5.37	5.81	0.44
4784	NFIX	4.65	9.00E-06	2.00E-05	6.95	11.41	4.46
4821	NKX2-2	23.43	1.00E-44	9.00E-43	5.49	8.42	2.93
26257	NKX2-8	-1.99	0.049	0.04	5.56	5.16	-0.4
4824	NKX3-1	1.99	0.049	0.04	6.04	6.64	0.59
4861	NPAS1	-8.82	2.00E-14	2.00E-13	8.87	5.6	-3.27
7026	NR2F2	4.22	5.00E-05	1.00E-04	6.3	8.13	1.83
8013	NR4A3	3.29	0.001	0.002	5.46	5.97	0.51
9480	ONECUT2	6.16	1.00E-08	4.00E-08	5.8	6.96	1.16
130497	OSR1	5.5	3.00E-07	8.00E-07	5.89	8.91	3.02
401	PHOX2A	2.32	0.022	0.021	5.36	5.74	0.38
8929	PHOX2B	11.4	2.00E-20	4.00E-19	5.65	8.52	2.86
5307	PITX1	2.23	0.028	0.025	7.23	10.23	3
5453	POU3F1	-2.89	0.005	0.005	6.74	5.32	-1.43
28984	C13orf15	4.16	6.00E-05	1.00E-04	7.27	10.22	2.95
8403	SOX14	-2.08	0.04	0.034	6	5.49	-0.51
6909	TBX2	4.27	4.00E-05	8.00E-05	5.73	8.2	2.46
6926	TBX3	2.94	0.004	0.005	6.22	7.12	0.91
6928	HNF1B	7.74	5.00E-12	3.00E-11	5.55	7.48	1.93
3196	TLX2	5.25	8.00E-07	2.00E-06	5.25	5.93	0.68
7421	VDR	7.09	1.00E-10	7.00E-10	5.29	6.76	1.47
7490	WT1	2.68	0.008	0.009	5.66	5.95	0.29
7704	ZBTB16	7.65	8.00E-12	5.00E-11	5.46	7.79	2.33
9839	ZEB2	4.24	5.00E-05	9.00E-05	6.36	8.63	2.26
63976	PRDM16	11.39	3.00E-20	4.00E-19	5.35	7.76	2.41
10475	TRIM38	5.34	5.00E-07	1.00E-06	5.98	7.53	1.55
7593	MZF1	2.5	0.014	0.014	7.64	8.02	0.38
2000	ELF4	4.2	5.00E-05	1.00E-04	7.81	8.96	1.15
6939	TCF15	-3.26	0.001	0.002	6.64	5.67	-0.98
602	BCL3	3.15	0.002	0.003	6.98	8.59	1.61
347853	TBX10	7.97	2.00E-12	1.00E-11	5.83	7.56	1.73
89870	TRIM15	31.78	3.00E-57	4.00E-55	5.51	9.39	3.88
154796	AMOT	2.3	0.024	0.022	7.99	9.14	1.15
3200	HOXA3	8.27	3.00E-13	2.00E-12	5.29	6.23	0.95
64067	NPAS3	3.02	0.003	0.004	5.45	5.78	0.34
11189	TNRC4	9.96	5.00E-17	5.00E-16	5.34	7.4	2.06
4793	NFKBIB	-4.01	1.00E-04	2.00E-04	6.78	6.12	-0.66
10150	MBNL2	5.69	1.00E-07	3.00E-07	6.06	8.06	1.99
23414	ZFPM2	4.48	2.00E-05	4.00E-05	5.5	6.64	1.14

367	AR	-2.73	0.007	0.008	6.35	5.4	-0.95
10608	MXD4	6.17	1.00E-08	4.00E-08	10.15	11.72	1.57
9330	GTF3C3	-4.19	6.00E-05	1.00E-04	10.18	9.24	-0.94
9112	MTA1	-3.38	0.001	0.001	10.21	9.46	-0.75
4306	NR3C2	8.15	6.00E-13	4.00E-12	6.38	7.86	1.47
4602	MYB	2.36	0.02	0.019	9.02	10.46	1.44
79890	RIN3	4.32	3.00E-05	7.00E-05	5.33	6	0.67
55734	ZFP64	-2.61	0.01	0.011	6.92	6.09	-0.83
3229	HOXC13	-2.06	0.042	0.035	5.55	5.2	-0.35
116039	OSR2	8.97	9.00E-15	7.00E-14	5.39	6.65	1.26
6935	ZEB1	4.56	1.00E-05	3.00E-05	5.59	6.89	1.3
1396	CRIP1	6.17	1.00E-08	4.00E-08	7.53	10.96	3.43
7942	TFEB	8.13	7.00E-13	5.00E-12	5.36	6.45	1.08
79776	ZFHX4	2.51	0.013	0.013	6.03	7.19	1.15
3164	NR4A1	3.93	1.00E-04	3.00E-04	5.4	6.14	0.74
1674	DES	23.63	6.00E-45	4.00E-43	5.49	10.48	4.99
54760	PCSK4	-2.77	0.007	0.007	6.31	5.7	-0.61
3663	IRF5	2.36	0.02	0.019	5.41	5.61	0.2
56270	WDR45L	-2.42	0.017	0.017	9.95	9.29	-0.66
51042	ZNF593	-3.31	0.001	0.002	10.03	9.3	-0.73
4066	LYL1	5.46	3.00E-07	9.00E-07	6.89	8.86	1.97
6015	RING1	2.46	0.015	0.015	10.87	11.45	0.57
80758	PRR7	-4.77	6.00E-06	1.00E-05	7.08	5.86	-1.21
29915	HCFC2	4.61	1.00E-05	2.00E-05	5.89	6.79	0.9
5468	PPARG	8.79	2.00E-14	2.00E-13	5.5	6.94	1.44
23493	HEY2	-5.21	9.00E-07	2.00E-06	10.53	7.35	-3.18
3659	IRF1	7.22	7.00E-11	4.00E-10	7.73	10.06	2.33
51085	MLXIPL	6.48	3.00E-09	1.00E-08	6.48	8.52	2.04
8535	CBX4	3.03	0.003	0.004	6.37	7.39	1.02
2118	ETV4	-5.55	2.00E-07	6.00E-07	8.64	6.11	-2.53
6322	SCML1	5	2.00E-06	5.00E-06	6.46	7.78	1.32
7067	THRA	7.41	3.00E-11	2.00E-10	6.3	8.35	2.05
1879	EBF1	9.38	1.00E-15	9.00E-15	5.81	8.3	2.5
3717	JAK2	4.48	2.00E-05	4.00E-05	6.39	7.46	1.07
6596	HLTF	-2.27	0.025	0.023	9.04	7.92	-1.13
30820	KCNIP1	2.98	0.004	0.004	5.6	6.32	0.72
9219	MTA2	-3.23	0.002	0.002	9.61	8.85	-0.75
26040	SETBP1	4.28	4.00E-05	8.00E-05	7.78	9.92	2.14
1050	CEBPA	6.37	5.00E-09	2.00E-08	6.85	10.63	3.78
6595	SMARCA2	4.99	2.00E-06	6.00E-06	8.99	10.82	1.83
2309	FOXO3	3.3	0.001	0.002	10.35	11.33	0.98
56978	PRDM8	7.62	9.00E-12	5.00E-11	6.01	8.43	2.42
23040	MYT1L	4.75	6.00E-06	1.00E-05	5.31	5.78	0.46
5914	RARA	3.88	2.00E-04	3.00E-04	7.51	8.17	0.66
4005	LMO2	3.69	3.00E-04	5.00E-04	6.66	7.78	1.12
23119	HIC2	-2.62	0.01	0.01	10.78	9.93	-0.86

2122	EVI1	15.2	9.00E-29	2.00E-27	5.97	11.2	5.24
1397	CRIP2	5.58	2.00E-07	5.00E-07	7.09	10.35	3.26
1326	MAP3K8	2.18	0.032	0.028	6.99	8.13	1.14
861	RUNX1	3.87	2.00E-04	3.00E-04	5.55	6.59	1.04
4790	NFKB1	6.01	2.00E-08	9.00E-08	9.27	11.69	2.42
6662	SOX9	4.16	6.00E-05	1.00E-04	7.34	9.6	2.26
5990	RFX2	8.24	4.00E-13	3.00E-12	5.52	7.21	1.69
687	KLF9	3.5	7.00E-04	0.001	8.18	10.93	2.76
2273	FHL1	3.58	5.00E-04	8.00E-04	8.01	9.64	1.63
2295	FOXF2	15.38	3.00E-29	1.00E-27	5.63	9.17	3.54
688	KLF5	3.69	3.00E-04	6.00E-04	5.81	7.38	1.56
1044	CDX1	15.29	5.00E-29	2.00E-27	5.48	11.04	5.56
3131	HLF	2.93	0.004	0.005	5.46	5.86	0.4
3201	HOXA4	30.4	2.00E-55	3.00E-53	5.25	8.5	3.25
3215	HOXB5	3.43	9.00E-04	0.001	7.04	10.76	3.72
3726	JUNB	4.52	2.00E-05	3.00E-05	6.74	8.91	2.17
3899	AFF3	2.55	0.012	0.012	6.23	7.52	1.29
4208	MEF2C	8.85	2.00E-14	1.00E-13	5.41	6.8	1.4
4211	MEIS1	6.11	2.00E-08	6.00E-08	6.31	9.67	3.36
4212	MEIS2	5.17	1.00E-06	3.00E-06	6.26	8.36	2.11
4862	NPAS2	10.32	7.00E-18	8.00E-17	5.89	7.56	1.67
5328	PLAU	-2	0.048	0.039	9.58	8.03	-1.55
5629	PROX1	2.85	0.005	0.006	5.53	6.08	0.55
6776	STAT5A	3.27	0.001	0.002	7.19	8.53	1.33
6839	SUV39H1	-4.23	5.00E-05	9.00E-05	10.22	9	-1.22
7022	TFAP2C	-10.48	3.00E-18	4.00E-17	9.82	5.48	-4.34
8433	UTF1	-3.62	5.00E-04	7.00E-04	7.38	5.2	-2.17
8507	ENC1	3.41	9.00E-04	0.001	8.41	10.42	2.02
8804	CREG1	7.09	1.00E-10	7.00E-10	9.17	10.58	1.41
8928	FOXH1	-4.56	1.00E-05	3.00E-05	6.94	5.96	-0.98
1465	CSRP1	3.06	0.003	0.003	8.63	12.45	3.82
754	PTTG1IP	3.75	3.00E-04	5.00E-04	11.37	12.09	0.72
864	RUNX3	2.52	0.013	0.013	6.95	8.11	1.17
3169	FOXA1	5.76	8.00E-08	3.00E-07	6.26	10.66	4.4
5017	OVOL1	3.61	5.00E-04	7.00E-04	5.52	6.22	0.7
648	BMI1	4.89	4.00E-06	8.00E-06	8.6	10.36	1.77
2672	GFI1	8.79	2.00E-14	2.00E-13	5.35	6.42	1.07
3149	HMGB3	-10.22	1.00E-17	1.00E-16	11.33	8.5	-2.84
4782	NFIC	4.31	4.00E-05	7.00E-05	5.62	6.34	0.72
6658	SOX3	-7.33	4.00E-11	2.00E-10	8.94	5.31	-3.64
5813	PURA	5.61	2.00E-07	5.00E-07	6.42	7.82	1.4
4209	MEF2D	4.62	1.00E-05	2.00E-05	8.4	9.53	1.13
4929	NR4A2	2.37	0.02	0.019	5.96	7.36	1.4
3090	HIC1	4.52	2.00E-05	3.00E-05	5.97	6.87	0.9
3199	HOXA2	5.27	7.00E-07	2.00E-06	5.72	7.65	1.92
27287	VENTX	-6.62	1.00E-09	6.00E-09	8.44	5.74	-2.7

7775	ZNF232	-2.91	0.004	0.005	8.66	7.81	-0.84
8022	LHX3	-2.09	0.039	0.033	5.48	5.2	-0.28
26508	HEYL	4.13	7.00E-05	1.00E-04	7.37	9.47	2.1
9792	SERTAD2	3.09	0.003	0.003	9.92	10.93	1.01
23314	SATB2	2	0.047	0.039	7.63	8.58	0.94
23316	CUX2	-5.35	5.00E-07	1.00E-06	7.4	5.85	-1.55
7041	TGFB1I1	4.69	8.00E-06	2.00E-05	6.45	7.83	1.38
51621	KLF13	3.98	1.00E-04	2.00E-04	9.3	10.14	0.84
51111	SUV420H1	-5.37	4.00E-07	1.00E-06	8.1	7.26	-0.84
51127	TRIM17	-3.27	0.001	0.002	6.27	5.45	-0.83
10365	KLF2	5.68	1.00E-07	3.00E-07	7.4	11.46	4.06
51450	PRRX2	-7.16	1.00E-10	5.00E-10	8.98	6.2	-2.79
55922	NKRF	-2.52	0.013	0.013	8.44	7.77	-0.67
54345	SOX18	5.13	1.00E-06	3.00E-06	6.99	10.28	3.28
55888	ZNF167	-2.5	0.014	0.014	6.79	6.14	-0.65
3202	HOXA5	6.65	1.00E-09	5.00E-09	6.44	11.29	4.85
59336	PRDM13	-2.45	0.016	0.015	5.39	5.11	-0.28
8864	PER2	5.71	1.00E-07	3.00E-07	8.82	10.38	1.56
79943	ZNF696	-2.85	0.005	0.006	8.48	7.71	-0.77
3241	HPCAL1	4.04	1.00E-04	2.00E-04	7.44	8.51	1.07
80333	KCNIP4	13.68	2.00E-25	4.00E-24	5.49	6.81	1.33
3205	HOXA9	3.43	9.00E-04	0.001	5.47	6.25	0.77
23492	CBX7	3.02	0.003	0.004	7.83	8.92	1.09
7275	TUB	-4.63	1.00E-05	2.00E-05	8.24	6.92	-1.32
1831	TSC22D3	6.22	9.00E-09	4.00E-08	6.46	8	1.54
6943	TCF21	12.55	6.00E-23	1.00E-21	5.61	9.3	3.69
2104	ESRRG	4.02	1.00E-04	2.00E-04	6.26	7.22	0.97

Table S5: This table lists all the TFs from Table S1, which are significantly differentially expressed between 107 hESC samples and 2 colon samples (1 fetal + 1 adult) from the SCM2 expression compendium. We give the Entrez gene ID, the symbol, the regularised t-statistic, its P-value, its adjusted P-value, the average expression level in hESC and tissue and the log fold change.

EntrezID	Symbol	t(LSCC)	P(LSCC)	t(LUAD)	P(LUAD)	t(KIRC)	P(KIRC)	t(KIRP)	P(KIRP)	t(BLCA)	P(BLCA)	t(COAD)	P(COAD)
3170	FOXA2	-10.6	7.00E-24	-5.17	3.00E-07	-0.5	0.618	-2.7	0.007	1.44	0.151	8.89	5.00E-17
3087	HHEX	-4.77	2.00E-06	-6.43	3.00E-10	8.37	4.00E-16	-4.12	5.00E-05	-1.9	0.058	-5.87	1.00E-08
26468	LHX6	-6.43	3.00E-10	-8.91	8.00E-18	4.37	1.00E-05	-8.36	2.00E-15	-2.47	0.014	-3.16	0.002
6886	TAL1	-18.74	4.00E-60	-22.1	4.00E-77	5.03	7.00E-07	-9.68	1.00E-19	-4.35	2.00E-05	-7.1	8.00E-12
6899	TBX1	6.15	2.00E-09	-0.54	0.588	-0.62	0.536	-3.76	2.00E-04	1.87	0.062	-5.93	8.00E-09
7080	NKX2-1	-10.54	1.00E-23	-1.71	0.088	1.07	0.283	-2.48	0.013	2.37	0.018	3.4	8.00E-04
7539	ZFP37	-0.27	0.79	-1.88	0.061	-3.15	0.002	-4.83	2.00E-06	-1.89	0.059	-1.13	0.261
7691	ZNF132	-6.49	2.00E-10	-4.87	1.00E-06	-12.2	1.00E-30	-4.23	3.00E-05	-2.11	0.036	-11.31	5.00E-25
23774	BRD1	2.68	0.007	0	0.997	3.07	0.002	-0.41	0.683	-1.91	0.057	-1.06	0.29
56731	SLC2A4RG	5.86	8.00E-09	4.81	2.00E-06	1.23	0.219	0.81	0.417	1.45	0.148	1.3	0.194
652	BMP4	-6.54	1.00E-10	-2.62	0.009	-2.77	0.006	-0.04	0.969	-3.47	6.00E-04	8.03	2.00E-14
8850	KAT2B	-15.76	6.00E-46	-12.86	4.00E-33	-10.67	2.00E-24	-9.6	2.00E-19	-4.4	1.00E-05	-12.88	1.00E-30
2119	ETV5	-5.04	7.00E-07	-9.9	3.00E-21	5.55	4.00E-08	1.64	0.101	0.9	0.37	4.96	1.00E-06
1749	DLX5	18.21	1.00E-57	6.15	2.00E-09	18.68	2.00E-61	0.26	0.795	2.95	0.003	3.37	9.00E-04
2004	ELK3	-5.46	8.00E-08	-1.31	0.192	2.87	0.004	-4.6	6.00E-06	-1.32	0.186	0.6	0.551
10138	YAF2	-0.15	0.88	-1.29	0.196	7.01	7.00E-12	1.72	0.087	-2.03	0.043	5.92	8.00E-09
7745	ZNF192	-1.66	0.097	-1.56	0.12	-2.39	0.017	-2.46	0.014	-2.01	0.045	0.86	0.391
6258	RXRG	-10.35	6.00E-23	-9.84	4.00E-21	-3.96	8.00E-05	-9.79	6.00E-20	-7.21	4.00E-12	-11.17	1.00E-24
9496	TBX4	-15.26	1.00E-43	-13.18	2.00E-34	4.41	1.00E-05	1.61	0.109	-4.27	3.00E-05	3.12	0.002

Table S6: This table lists the differential expression statistics (moderated t-statistic (t) and P-value (P)) of <u>lung-specific TFs</u> in six TCGA data sets, including LUAD (lung adenomacarcinoma), LSCC (lung squamous cell carcinoma), KIRC (kidney renal cell carcinoma), KIRP (kidney renal papillary carcinoma), BLCA (bladder cancer) and COAD (Colon adenoma carcinoma). The lung-specific TFs are those overexpressed in the SCM2 lung samples compared to hESCs, and not overexpressed in the other three tissue types.

EntrezID	Symbol	t(KIRC)	P(KIRC)	t(KIRP)	P(KIRP)	t(LSCC)	P(LSCC)	t(LUAD)	P(LUAD)	t(BLCA)	P(BLCA)	t(COAD)	P(COAD)
4435	CITED1	-3.63	3.00E-04	-3.63	3.00E-04	-3.53	5.00E-04	-4.04	6.00E-05	-0.62	0.537	7.02	1.00E-11
8110	DPF3	3.03	0.003	-5.09	6.00E-07	-5.01	7.00E-07	-1.54	0.125	-3.41	7.00E-04	-11.46	1.00E-25
3218	HOXB8	-14.27	7.00E-40	-2.96	0.003	2.78	0.006	0.74	0.46	2.75	0.006	6.26	1.00E-09
3222	HOXC5	-1.76	0.078	-2.48	0.013	6.22	1.00E-09	3.6	3.00E-04	1.24	0.215	-0.19	0.847
3223	HOXC6	-1.37	0.172	-2.22	0.027	10.58	8.00E-24	4.82	2.00E-06	2.62	0.009	1.33	0.185
3224	HOXC8	0.93	0.354	0.61	0.541	10.76	2.00E-24	4.26	2.00E-05	1.65	0.099	2.1	0.036
3235	HOXD9	-2.44	0.015	-7.15	6.00E-12	6.43	3.00E-10	-0.28	0.776	1.35	0.178	-0.17	0.867
84504	NKX6-2	-19.08	2.00E-63	-10.89	1.00E-23	-3.93	1.00E-04	-4.69	3.00E-06	-2.78	0.006	0.21	0.832
5076	PAX2	-2.25	0.025	-0.13	0.896	3.43	7.00E-04	-0.08	0.935	0.74	0.46	1.7	0.09
6493	SIM2	-29.49	6.00E-118	-12.57	1.00E-29	8.52	2.00E-16	2.01	0.045	5.14	5.00E-07	16.55	2.00E-44

6495	SIX1	14.14	3.00E-39	-0.82	0.412	12.75	1.00E-32	10.21	2.00E-22	5.29	2.00E-07	14.37	3.00E-36
2103	ESRRB	-24.45	1.00E-91	-14.01	4.00E-35	-1.45	0.148	-2.61	0.009	-0.2	0.84	-1.65	0.099
7020	TFAP2A	-15.1	1.00E-43	-10.76	3.00E-23	27.83	6.00E-105	14.97	2.00E-42	4.8	2.00E-06	7.67	2.00E-13
7021	TFAP2B	-19.19	4.00E-64	-15.11	3.00E-39	3.76	2.00E-04	-2.83	0.005	-1.78	0.077	-7.89	5.00E-14
3236	HOXD10	-10.54	6.00E-24	-11.55	5.00E-26	10.68	3.00E-24	2.16	0.031	-0.18	0.854	-3.25	0.001
2016	EMX1	-13.02	3.00E-34	-5.3	2.00E-07	10.76	2.00E-24	4.98	9.00E-07	4.76	3.00E-06	-3.09	0.002
6492	SIM1	-10.31	5.00E-23	-5.83	1.00E-08	2.63	0.009	4.48	9.00E-06	0.03	0.979	-3.35	9.00E-04
2303	FOXC2	3.74	2.00E-04	-7.56	4.00E-13	-2.43	0.015	-5.26	2.00E-07	-1.71	0.088	-0.31	0.755
3975	LHX1	-15.28	1.00E-44	-9.01	2.00E-17	5.84	9.00E-09	5.18	3.00E-07	4.07	6.00E-05	3.7	3.00E-04
7746	ZNF193	6.76	3.00E-11	4.36	2.00E-05	0.3	0.762	5.85	8.00E-09	1.95	0.053	2.53	0.012
6772	STAT1	2.09	0.037	2.25	0.025	2.08	0.038	4.85	2.00E-06	1.82	0.07	2.1	0.036
22882	ZHX2	7.43	4.00E-13	3.68	3.00E-04	-2.97	0.003	-6.04	3.00E-09	-5.5	7.00E-08	1.54	0.125
23316	CUX2	-0.45	0.651	-2.52	0.012	-2.51	0.012	-0.27	0.786	-3.14	0.002	-7.87	6.00E-14
3226	HOXC10	-1.43	0.152	-1.4	0.164	10.39	4.00E-23	5.77	1.00E-08	-1.05	0.293	0.09	0.927
3219	HOXB9	-18.4	5.00E-60	-4.24	3.00E-05	9.78	8.00E-21	7.55	2.00E-13	3.56	4.00E-04	0.8	0.422

Table S7: This table lists the differential expression statistics (moderated t-statistic (t) and P-value (P)) of <u>kidney-specific TFs</u> in six TCGA data sets, including LUAD (lung adenomacarcinoma), LSCC (lung squamous cell carcinoma), KIRC (kidney renal cell carcinoma), KIRP (kidney renal papillary carcinoma), BLCA (bladder cancer) and COAD (Colon adenoma carcinoma). The kidney-specific TFs are those overexpressed in the SCM2 kidney samples compared to hESCs, and not overexpressed in the other three tissue types.

EntrezID	Symbol	t(BLCA)	P(BLCA)	t(LSCC)	P(LSCC)	t(LUAD)	P(LUAD)	t(KIRC)	P(KIRC)	t(KIRP)	P(KIRP)	t(COAD)	P(COAD)
467	ATF3	-8.1	1.00E-14	-8.68	5.00E-17	-10.01	1.00E-21	-6.16	1.00E-09	-6.99	2.00E-11	-0.49	0.622
3670	ISL1	-4.19	4.00E-05	3.7	2.00E-04	2.3	0.022	-1.43	0.152	0.24	0.813	-2.29	0.023
22806	IKZF3	0.89	0.376	-3.87	1.00E-04	1.76	0.079	7.88	2.00E-14	-0.44	0.659	-5.5	8.00E-08
8863	PER3	-6.3	9.00E-10	-5.42	9.00E-08	-5.09	5.00E-07	-0.28	0.777	-5.22	3.00E-07	-8.49	9.00E-16
406	ARNTL	-1.72	0.087	-0.93	0.354	-1.84	0.066	4.27	2.00E-05	1.58	0.115	-8.99	2.00E-17
2100	ESR2	0.32	0.753	2.36	0.018	0.97	0.334	-4.97	9.00E-07	-2.49	0.013	-6.3	1.00E-09
2099	ESR1	-6.23	1.00E-09	-6.34	5.00E-10	-0.38	0.707	-5.91	6.00E-09	-5.27	3.00E-07	-6.56	2.00E-10
3229	HOXC13	4.09	5.00E-05	24.42	3.00E-88	7.92	1.00E-14	11.47	1.00E-27	3.74	2.00E-04	3.75	2.00E-04
27086	FOXP1	-4.96	1.00E-06	-12.34	7.00E-31	-8.49	2.00E-16	-3.59	4.00E-04	-1.86	0.063	-3.89	1.00E-04
10848	PPP1R13L	5.32	2.00E-07	4.61	5.00E-06	1.3	0.194	13.3	2.00E-35	3.83	2.00E-04	8.8	1.00E-16
28999	KLF15	-4.27	3.00E-05	-11.36	8.00E-27	-9.1	2.00E-18	-6.17	1.00E-09	-3.58	4.00E-04	-5.52	7.00E-08
2274	FHL2	-1.56	0.119	6.69	6.00E-11	11.02	1.00E-25	-0.69	0.489	1.02	0.309	2.6	0.01
1958	EGR1	-9.52	3.00E-19	-9.45	1.00E-19	-9.92	2.00E-21	-5.25	2.00E-07	-6.8	5.00E-11	-1.12	0.262
4488	MSX2	-1.37	0.173	3.47	6.00E-04	2.29	0.022	0	1	5.2	4.00E-07	15.18	2.00E-39
8553	BHLHB2	-2.93	0.004	-2.87	0.004	-0.75	0.456	2.67	0.008	-1.49	0.137	10.92	1.00E-23
860	RUNX2	3.35	9.00E-04	6.59	1.00E-10	10.74	2.00E-24	11.68	2.00E-28	5.23	3.00E-07	-0.68	0.499
2353	FOS	-8.64	2.00E-16	-12.05	1.00E-29	-9.31	3.00E-19	-4.79	2.00E-06	-7.28	3.00E-12	-3.63	3.00E-04
2355	FOSL2	-4.4	1.00E-05	1.38	0.168	-3.44	6.00E-04	-5.23	2.00E-07	-3.49	5.00E-04	-7.43	1.00E-12
4783	NFIL3	-6.44	4.00E-10	-3.38	8.00E-04	-7.04	6.00E-12	4.82	2.00E-06	-1.49	0.138	-1.8	0.073

2354	FOSB	-8.51	6.00E-16	-14.85	8.00E-42	-11.88	5.00E-29	-2.07	0.039	-7.64	3.00E-13	-2.79	0.006
6096	RORB	-4.36	2.00E-05	-6.55	1.00E-10	-4.83	2.00E-06	-9.29	3.00E-19	-7.48	7.00E-13	-5.91	9.00E-09
23764	MAFF	-6.65	1.00E-10	-8.48	2.00E-16	-11.06	1.00E-25	0.8	0.421	-3.7	3.00E-04	3.12	0.002
23413	FREQ	-7.3	2.00E-12	12.22	2.00E-30	4.63	5.00E-06	-11.46	1.00E-27	-2.57	0.011	-1.89	0.06
51341	ZBTB7A	-3.22	0.001	-2.52	0.012	-5.8	1.00E-08	1.04	0.3	0.59	0.557	-9.13	9.00E-18

Table S8: This table lists the differential expression statistics (moderated t-statistic (t) and P-value (P)) of bladder-specific TFs in six TCGA data sets, including LUAD (lung adenomacarcinoma), LSCC (lung squamous cell carcinoma), KIRC (kidney renal cell carcinoma), KIRP (kidney renal papillary carcinoma), BLCA (bladder cancer) and COAD (Colon adenoma carcinoma). The bladder-specific TFs are those overexpressed in the SCM2 bladder samples compared to hESCs, and not overexpressed in the other three tissue types.

EntrezID	Symbol	t(COAD)	P(COAD)	t(LSCC)	P(LSCC)	t(LUAD)	P(LUAD)	t(KIRC)	P(KIRC)	t(KIRP)	P(KIRP)	t(BLCA)	P(BLCA)
474	ATOH1	-5.85	1.00E-08	2.57	0.011	2.62	0.009	-0.2	0.842	1.01	0.313	1.16	0.248
8538	BARX2	-8.28	4.00E-15	6.96	1.00E-11	10.64	4.00E-24	11.9	2.00E-29	-2.31	0.021	4.08	6.00E-05
1045	CDX2	-3.24	0.001	4.61	5.00E-06	6.38	4.00E-10	0.61	0.544	-0.74	0.462	2.14	0.033
54738	FEV	-12.19	3.00E-28	-1.85	0.065	-1.48	0.139	1.2	0.229	-2.64	0.009	-3.96	9.00E-05
2626	GATA4	-1.04	0.298	1.07	0.285	-1.39	0.165	1.82	0.069	1.11	0.268	-0.44	0.66
3725	JUN	1.62	0.105	-8.28	1.00E-15	-8.93	7.00E-18	1.92	0.055	-4.4	1.00E-05	-7.53	5.00E-13
4760	NEUROD1	-10.35	9.00E-22	-4.54	7.00E-06	0.43	0.665	-6.64	7.00E-11	-10.29	1.00E-21	0.53	0.599
8013	NR4A3	-2.07	0.04	-12.96	2.00E-33	-10.93	3.00E-25	-6.03	3.00E-09	-11.67	2.00E-26	-9.25	2.00E-18
9480	ONECUT2	7.68	2.00E-13	9.95	2.00E-21	8.29	9.00E-16	-4.41	1.00E-05	5.97	6.00E-09	4.12	5.00E-05
401	PHOX2A	-10.43	5.00E-22	2.84	0.005	3.07	0.002	4.57	6.00E-06	-1.02	0.309	0.66	0.511
7421	VDR	-9.28	3.00E-18	-1.5	0.135	4	7.00E-05	-12.46	9.00E-32	-7.84	7.00E-14	2.57	0.011
347853	TBX10	-5.95	7.00E-09	4.21	3.00E-05	5.37	1.00E-07	1.43	0.154	0.33	0.743	1.38	0.168
89870	TRIM15	-4.11	5.00E-05	5.5	6.00E-08	7.7	7.00E-14	4.48	9.00E-06	0.56	0.577	3.01	0.003
11189	TNRC4	-4.99	1.00E-06	-2.36	0.018	2.18	0.03	-5.42	9.00E-08	-6.65	1.00E-10	-1.43	0.154
4602	MYB	2.75	0.006	1.8	0.073	-2	0.046	15.83	3.00E-47	8.9	4.00E-17	3.53	5.00E-04
51085	MLXIPL	11.89	4.00E-27	-2.66	0.008	-5.23	2.00E-07	2.94	0.003	0.4	0.692	-0.24	0.814
23040	MYT1L	-11.25	7.00E-25	-5.78	1.00E-08	-4.09	5.00E-05	1.7	0.089	0.36	0.716	-6.34	7.00E-10
1044	CDX1	-4.41	1.00E-05	7.83	3.00E-14	7.71	6.00E-14	1.74	0.083	-3.84	1.00E-04	0.85	0.397

Table S9: This table lists the differential expression statistics (moderated t-statistic (t) and P-value (P)) of colon-specific TFs in six TCGA data sets, including LUAD (lung adenomacarcinoma), LSCC (lung squamous cell carcinoma), KIRC (kidney renal cell carcinoma), KIRP (kidney renal papillary carcinoma), BLCA (bladder cancer) and COAD (Colon adenoma carcinoma). The colon-specific TFs are those overexpressed in the SCM2 colon samples compared to hESCs, and not overexpressed in the other three tissue types.

	Relative to Random		Relative to Biv/PRC2 TFs	
	OR	P	OR	P
**LSCC**	3.92	5e-6	0.78	0.54
**LUAD**	4.36	2e-6	0.86	0.68
**KIRC**	7.3	1e-6	1.44	0.63
**KIRP**	4.89	3e-6	0.97	1
**BLCA**	4.95	2e-6	0.98	1
**COAD**	3.65	1e-5	0.72	0.43

Table S10: This table lists the Odds Ratios (OR) and associated Fisher-test P-values (P) that the TFs silenced in the given cancer type have a promoter mapping to a CpG island (using the UCSC genome browser definition of CpG islands), compared to a random set of over 1000 genes (Random) or compared to the initial starting list of the 403 bivalently/PRC2 marked TFs.

References

1. Lee, T.I. *et al.* Control of developmental regulators by Polycomb in human embryonic stem cells. *Cell* **125**, 301-13 (2006).
2. Bernstein, B.E. *et al.* A bivalent chromatin structure marks key developmental genes in embryonic stem cells. *Cell* **125**, 315-26 (2006).
3. Nazor, K.L. *et al.* Recurrent variations in DNA methylation in human pluripotent stem cells and their differentiated derivatives. *Cell Stem Cell* **10**, 620-34 (2012).
4. Muller, F.J. *et al.* Regulatory networks define phenotypic classes of human stem cell lines. *Nature* **455**, 401-5 (2008).
5. Nejman, D. *et al.* Molecular rules governing de novo methylation in cancer. *Cancer Res* **74**, 1475-83 (2014).
6. Smyth, G.K. Linear models and empirical bayes methods for assessing differential expression in microarray experiments. *Stat Appl Genet Mol Biol* **3**, Article3 (2004).
7. Wettenhall, J.M. & Smyth, G.K. limmaGUI: a graphical user interface for linear modeling of microarray data. *Bioinformatics* **20**, 3705-6 (2004).
8. Cancer Genome Atlas Research, N. Comprehensive molecular profiling of lung adenocarcinoma. *Nature* **511**, 543-50 (2014).
9. Cancer Genome Atlas Research, N. Comprehensive genomic characterization of squamous cell lung cancers. *Nature* **489**, 519-25 (2012).
10. Cancer Genome Atlas Research, N. Comprehensive molecular characterization of clear cell renal cell carcinoma. *Nature* **499**, 43-9 (2013).
11. Cancer Genome Atlas Research, N. Comprehensive molecular characterization of urothelial bladder carcinoma. *Nature* **507**, 315-22 (2014).

12. Cancer Genome Atlas Research, N. Comprehensive molecular characterization of gastric adenocarcinoma. *Nature* **513**, 202-9 (2014).
13. Teschendorff, A.E. *et al.* A beta-mixture quantile normalization method for correcting probe design bias in Illumina Infinium 450 k DNA methylation data. *Bioinformatics* **29**, 189-96 (2013).
14. Teschendorff, A. *Computational and Statistical Epigenomics*, 217 (Springer, 2015).
15. Jiao, Y., Widschwendter, M. & Teschendorff, A.E. A systems-level integrative framework for genome-wide DNA methylation and gene expression data identifies differential gene expression modules under epigenetic control. *Bioinformatics* (2014).
